# Recent Advances in Cation-Engineered A_3_BX_6_ Metal Halide Perovskite for Enhanced Radiative Transition

**DOI:** 10.34133/research.1281

**Published:** 2026-05-18

**Authors:** Xin Zhou, Hang Yin, Qinhua Wei, Da Chen, Dongfeng Xue, Tönu Pullerits, Junsheng Chen, Laishun Qin

**Affiliations:** ^1^ College of Materials and Chemistry, China Jiliang University, Hangzhou 310018, China.; ^2^ Chemical Physics and NanoLund, Lund University, Lund 22100, Sweden.; ^3^ Nano-Science Center and Department of Chemistry, University of Copenhagen, Copenhagen 2100, Denmark.; ^4^ Shenzhen Institute for Advanced Study, University of Electronic Science and Technology of China, Shenzhen 518110, China.

## Abstract

A_3_BX_6_ perovskites, a family of vacancy-ordered structures, exhibit diverse luminescence behaviors upon photon, electron, and high-energy excitation, primarily originating from intrinsic self-trapped excitons or dopant-induced electronic transitions. Upon B-site cation engineering, ns^2^ cations tune intrinsic luminescence, whereas transition-metal and rare-earth dopants activate characteristic d–d, d–f, and f–f transitions, enriching A_3_BX_6_ optical diversity. The tunable crystal structure and electronic configuration endow A_3_BX_6_ perovskites with exceptional versatility for photoluminescence, electroluminescence, and scintillation applications. This review systematically elucidates how B-site chemistry modulates the structure–property–application relationships in this material family. P-block B-site A_3_BX_6_ perovskites exhibit high photoluminescence efficiency, broadband emission, and strong ultraviolet absorption, enabling applications in high-sensitivity photodetectors (1.23 × 10^12^ Jones), information encryption, and white light-emitting diodes. In comparison, rare-earth-based A_3_BX_6_ perovskites enable high-efficiency electroluminescent devices, featuring deep-blue light-emitting diode with an external quantum efficiency of 7.9%. Moreover, they exhibit superior scintillation performance, including high x-ray light yield (88,800 ph/MeV), low x-ray detection limit (63 nGy/s), and notable γ-ray response under ^137^Cs excitation (47,000 ph/MeV; 4.0% energy resolution). These insights highlight the pivotal role of B-site cation engineering in tailoring luminescence mechanisms and enabling multifunctional A_3_BX_6_ perovskites for photonic and radiation applications.

## Introduction

Radiative transitions, as the core mechanism of energy release in quantum theory, not only elucidate light–matter interactions but also enable breakthroughs in optoelectronic conversion, high-energy scintillation imaging, and quantum light sources, bridging photophysical mechanisms and practical applications [1]. Both scintillation and luminescence possess cross-domain similarities in photophysical mechanisms, particularly in excitation modalities, radiative decay pathways, fluorescent spectral range (ultraviolet [UV] or visible), energy conversion and migration process, and charge carrier dynamics [2,3]. More recently, metal halide perovskites (MHPs) have emerged as potential luminescent scintillators, leveraging their superior photoelectric properties for applications spanning photoluminescence (PL), electroluminescence (EL), and radioluminescence (RL) [4–7]. For example, the applied research for CsPbX_3_ centered on the properties of PL and EL prior to 2015, while a breakthrough emerged in 2018 with its RL application in x-ray and γ-ray detection as efficient scintillators [8,9]. Following these breakthroughs, it has been increasingly recognized that high-energy scintillation performance is highly sensitive to compositional tunability, where cation substitution directly influences crystal structure, electronic configuration, and defect landscape, thereby modulating light yield (L.Y.), energy resolution (E.R.), and time response [10]. Thus, the spotlight has gradually connected application demonstrations with cation-engineered strategies, which is crucial for tailoring the electronic structure, regulating radiative transitions, and ultimately improving the scintillation performance of MHPs.

**Fig. 1.**
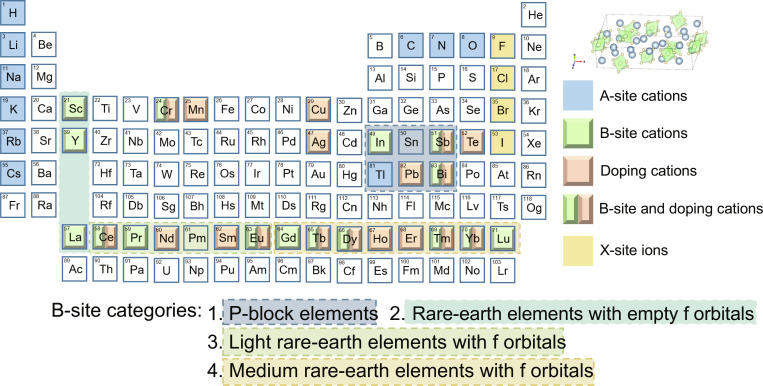
A-site cations, B-site cations, and X-site halogen anions of A_3_BX_6_ metal halide perovskites (MHPs) are illustrated in the periodic table. (C, H, O, and N form organic groups occupying the A-site). The blue–gray dashed box denotes P-block elements; the teal–gray dashed box represents rare-earth elements with empty f orbitals; the light-olive dashed box indicates light rare-earth elements with f orbitals; and the sand-yellow dashed box corresponds to heavy rare-earth elements with f orbitals.

To mitigate the toxicity of Pb^2+^, as well as the radiation sensitivity and poor stability of CsPbX_3_ [11], lead-free A_3_BX_6_ MHPs (strictly speaking, A_3_BX_6_ compounds are perovskite-derived structures [12]; however, they are discussed here within the framework of MHPs) are priorities for potential applications(Fig. S1) in anticounterfeiting, light-emitting diodes (LEDs) [13,14], photodetectors [15], and x-ray or γ-ray detection [16,17], due to their tunable electronic structure [16,18], highly tolerant crystallographic lattices [19,20], low phonon energies [21,22], and functional luminescence [19,23]. Recent advancements in A_3_BX_6_ systems have expanded the elemental scope across the periodic table, encompassing A-site cations (in blue), B-site cations (in green), halide anions (in yellow), and dopant ions (in espresso), as systematically summarized in Fig. 1. Importantly, the selection of B-site cations governs the electronic and structural characteristics, serving as a pivotal determinant for tuning luminescent behavior and functional performance through strategic doping engineering. According to the periodic table (Fig. 1), chemically accessible B-site cations in A_3_BX_6_ systems are classified; the B-site host engineering can be grouped into 3 categories: P-block elements, rare-earth (RE) elements with empty f orbitals (RE^3+^: Sc^3+^, Y^3+^, and La^3+^. It should be noted that La^3+^ has empty 4f orbitals, whereas Sc^3+^ and Y^3+^ lack f orbitals. For consistency, both cases are collectively classified as “empty-f” systems in this work.), and trivalent lanthanides (Ln^3+^, which are divided into light REs and heavy REs) with f orbitals. Accordingly, this review highlights the B-site cation-centered categorization of A_3_BX_6_ MHPs and explores their functional applications.

## The Luminescence Mechanism of Host and Dopant Engineering

### Intrinsic luminescence

Substituting Pb^2+^ with lead-free cations to form A_3_BX_6_ reduces the Goldschmidt tolerance factor, driving a structural transition from a 3-dimensional ABX_3_ to a zero-dimensional (0D) A_3_BX_6_. This dimensional change results in distinct electronic structures and offers opportunities for tuning structure–property relationships, particularly via symmetry control and orbital interactions. The crystal structure of A_3_BX_6_ with the *C*2/*c* space group features alternating octahedra with 2 distinct tilt angles, due to the different 2 independent site occupations of A cation [24]. The low charge symmetry in 0D A_3_BX_6_ MHPs favors the charge localization in both the ground and excited states, further affecting the formation of self-trapped excitons (STEs) and second-order Jahn–Teller distortion [25]. In general, this distortion in A_3_BX_6_ is derived from lone-pair electrons of ns^2^ cations, where the antibonding orbitals formed between B-site cation and X-site anion can hybridize with the unoccupied p orbitals of the cations.

**Fig. 2.**
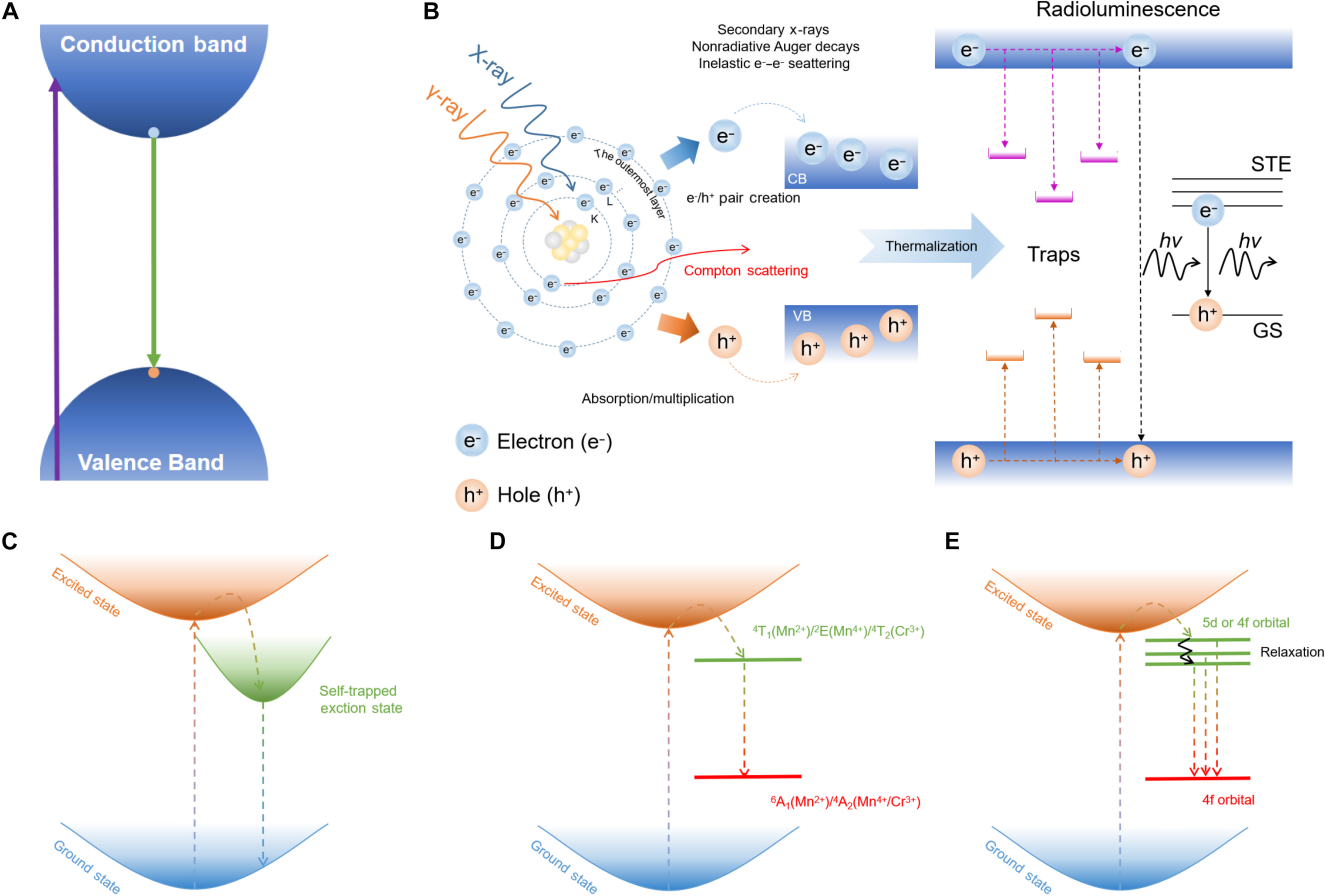
(A) The band-edge luminescence mechanism diagram of CsPbBr_3_. (B) The luminescence process triggered by different excitation sources. GS, ground state. (C) The luminescence mechanism of self-trapped exciton (STE) (including intrinsic STE and STE caused by doping). (D) The luminescence mechanism of transition-metal-doped luminescent ions (Mn^2+^, Mn^4+^, and Cr^3+^). (E) The d–f/f–f transition mechanism of rare earth.

Unlike CsPbX_3_ perovskites, where intrinsic luminescence primarily arises from band-edge exciton recombination (Fig. 2A) [26], A_3_BX_6_ MHPs crucially exhibit intrinsic STE emission leading to large Stokes shifts and broad emission spectra [27]. According to the self-trapping criterion, which is expressed as Eq. (1) [25]:

$$E_{\mathrm{st}}=\frac{W}{2}$$ (1)

where *W* represents the bandgap width and *E*_st_ is the self-trapping energy, which can be calculated using Eq. (2) [25]:

$$E_{\mathrm{st}}=E_{\text{free}}-E_{\text{trap}}$$ (2)

where *E*_free_ represents the free exciton energy and *E*_trap_ represents the STE state energy. Compared with 3D ABX_3_, the 0D A_3_BX_6_ structure exhibits more pronounced local lattice distortion and stronger lattice relaxation, which lowers the energy of the *E*_trap_. As a result, *E*_st_ increases and more readily satisfies Eq. (1), thereby facilitating the formation of STEs. In addition, the emission energy can also be described by Eq. (3) [28]:

$$E_{\mathrm{PL}}=W-E_{b}-E_{\mathrm{st}}-E_{d}$$ (3)

where *E*_b_ represents the exciton binding energy and *E*_d_ represents the lattice distortion energy. Compared with APbX_3_, A_3_BX_6_ exhibits larger *E*_st_ and *E*_d_, which results in *E*_PL_ < *E*_g_. Consequently, this leads to a larger Stokes shift and effectively suppresses self-absorption.

Under high-energy excitation such as x-rays and γ-rays, energy deposition occurs through multiple processes (Fig. 2B) including the photoelectric effect, Compton scattering, and subsequent electron–hole pair generation [29]. Different types of high-energy radiation interact with materials through distinct mechanisms, resulting in different energy deposition characteristics. X-rays in the kiloelectronvolt range primarily deposit energy via the photoelectric effect, whereas higher-energy γ-rays mainly undergo Compton scattering, often accompanied by photoelectric absorption or pair production, leading to more spatially distributed energy deposition and differences in carrier dynamics and scintillation behavior. High-energy radiation generates ions and core-excited electrons, giving rise to a cascade of secondary electrons. It is particularly noteworthy that the lower-energy segment of these secondary electrons is most efficient at producing the low-lying excited states responsible for luminescence [30]. As a result, the scintillation superiority of A_3_BX_6_ MHPs is characterized by low self-absorption, high photon output efficiency, and broad spectral response compatible with photomultiplier tube or silicon photomultiplier [31,32]. However, the isolated octahedra in 0D A_3_BX_6_ MHPs are susceptible to point defects or heterogeneous interactions, causing energy disorder that suppresses emission [33,34]. Hence, the STE emission could be improved by dopant-related luminescence.

### Dopant-related luminescence

To enhance the photoelectric properties of A_3_BX_6_ MHPs, B-site dopant engineering has emerged as an effective strategy for band structure modulation, which can be divided into 4 categories (Fig. 2C to E):

1. ns^2^ cations as B-site dopant. Incorporation of ns^2^ cations into the A_3_BX_6_ host induces the modified lattice distortion, via size mismatch (ionic radius disparities causing local strain), lone-pair effects (asymmetric coordination distorting [BX_6_]^3−^ octahedron), and charge redistribution (polarizable ns^2^ electrons altering electron density and bond lengths), thereby promoting STE formation and efficient broadband emission [35]. Most ns^2^ cations used as dopant in A_3_BX_6_ are summarized in Fig. 1, highlighting P-block elements (e.g., In, Sb, and Bi) into yellow dashed borders and Ln with empty f orbitals (e.g., Sc, Y, and La) into green dashed borders.
2. Transition-metal cations as B-site dopant. Incorporated transition-metal cations in A_3_BX_6_ can be summarized in dual luminescence regimes: One is as B-site luminescent center ions (e.g., atomic-like ionic d–d transitions) with sharp emission lines, and the other is STE emission (Fig. 2C). Herein, Mn^2+^ (3d^5^), Mn^4+^ (3d^3^), and Cr^3+^ (3d^3^) exhibit distinct d–d transitions governed by their crystal-field environments [36–38]. The Mn^2+^ typically dominates an orange–red broadband emission (550 to 650 nm) originating from ^4^T_1_ → ^6^A_1_ transition (Fig. 2D), which is enhanced by electron–phonon coupling in weak octahedral fields. In contrast, Mn^4+^ (^2^E → ^4^A_2_) and Cr^3+^ (^4^T_2_ → ^4^A_2_) emit narrow deep-red (630 to 650 nm) and tunable red to near-infrared (NIR) luminescence (650 to 800 nm), respectively, owing to spin-orbit or vibronic coupling within rigid and intermediate octahedral sites [39–41]. In addition, the incorporation of Ag^+^, Cu^+^, and Pt^4+^ can enhance the lattice distortion to facilitate the formation of STEs, further improving the efficiencies of PL and RL [42–45].
3. Ln^3+^ with f orbitals as B-site dopant. The incorporation of Ln^3+^ into the A_3_BX_6_ lattice (A_3_BX_6_:Ln^3+^) is facilitated by their ionic charge compatibility with the host B-site cation, enabling isovalent substitution without introducing extrinsic charge defects [46]. In addition, the superiority of A_3_BX_6_:Ln^3+^ arises from the unique shielding effect of the 5s^2^5p^6^ subshell in Ln^3+^, which confines f–f or d–f transitions within the large bandgap of the host matrix, thereby enabling tunable luminescence pathways via energy-level modulation [47]. This strategy not only preserves the intrinsic electrostatic balance in A_3_BX_6_ but also modulates the luminescence dynamics based on the localized 4f electrons of Ln^3+^. As illustrated in Fig. 2E, such intrabandgap engineering with different Ln^3+^ as B-site luminescent center enables characteristic f–f transitions (e.g., Tb^3+^) [48] or d–f charge–transfer transitions (e.g., Ce^3+^) [49]. The energy transfer (ET) efficiency between these pathways can be optimized by controlling the Ln^3+^ doping concentration and local coordination environment. Specifically, ET or defect-state coupling activates dual-mode emission in A_3_BX_6_:Ln^3+^, coexisting with broadband STE emission [50]. By tailoring the B-site dopant composition and concentration, the dual-mode emission can be further tuned to match the spectral sensitivity of photodetectors (e.g., photomultiplier tube or silicon photomultiplier), further improving the scintillation performance, such as L.Y., E.R., and time response. The distinct combination of tunable luminescence, structural stability, and radiation responsiveness positions A_3_BX_6_ MHPs as highly versatile materials for optoelectronic and scintillation applications. Recent work focusing on B-site scalable synthesis, dopant–host matching, and interface engineering will be essential for advancing their practical implementations.

4. ET processes induced by single or codoping. Upon incorporation of luminescent ions into the A_3_BX_6_ lattice, their emission can arise not only from direct excitation of dopant-specific energy levels but also from ET from intrinsic emissive states of the host lattice. Such ET processes can be broadly classified into 3 categories: STE to Ln^3+^, STE to transition metal, and Ln^3+^–Ln^3+^. In vacancy-ordered A_3_BX_6_ MHPs featuring isolated [BX_6_]^3−^ octahedra, ET between intrinsic emission and dopant activators can be consistently rationalized within a Förster–Dexter framework [51,52]. The key criterion for distinguishing Förster resonance ET (FRET) from Dexter ET lies in their characteristic transfer distances. FRET originates from long-range Coulombic dipole–dipole interactions and can operate over distances of approximately 1 to 10 nm without requiring wavefunction overlap, whereas Dexter ET is a short-range electron exchange process that relies on direct orbital overlap and therefore becomes effective only at subnanometer distances (<1 nm) [53]. In A_3_BX_6_ systems, the isolated arrangement of [BX_6_]^3−^ octahedra leads to increased effective donor–acceptor separations compared to more strongly connected structures. As a result, STE–Ln^3+^ and Ln^3+^–Ln^3+^ ET are predominantly governed by FRET, which is predominantly mediated by dipole–quadrupole and quadrupole–quadrupole interactions, while dipole–dipole coupling is typically much weaker. In contrast, STE–transition metal ET is more likely dominated by Dexter exchange interactions because transition-metal d–d excited states are spin- and parity-forbidden, efficient sensitization typically requires short-range exchange coupling at subnanometer distances, as evidenced in 0D isolated octahedra halides, where ET occurs exclusively via Dexter interactions between adjacent donor–acceptor pairs [54]. Thus, the isolated [BX_6_]^3−^ octahedral geometry in A_3_BX_6_ directly governs the effective donor–acceptor separation and, consequently, the energy transfer efficiency.

In addition to the forward energy transfer pathways, thermal back-transfer represents a ubiquitous quenching channel in A_3_BX_6_ systems [55]. When the excited-state energy levels of dopant activators are energetically close to those of the intrinsic emissive states (e.g., STEs or defect-related states), reverse ET from the dopant back to the host lattice can be thermally activated [56]. This process is typically governed by an Arrhenius-type behavior and becomes increasingly significant at elevated temperatures or under sustained excitation, leading to reduced emission intensity, shortened lifetimes, and compromised operational stability [56]. Owing to the strong localization of excitons and the discrete energy landscape imposed by isolated [BX_6_]^3−^ octahedra, A_3_BX_6_ materials are particularly susceptible to such thermally assisted back-transfer processes, which may coexist with nonradiative relaxation and defect trapping to collectively limit luminescence efficiency [57]. Consequently, suppressing thermal back-transfer through energy-level engineering and structural optimization is crucial for achieving high-efficiency and stable emission in A_3_BX_6_-based optoelectronic and scintillation applications.

### Concentration quenching effect

In doped A_3_BX_6_ systems, the emission intensity typically increases with dopant concentration at low levels and decreases rapidly after reaching the critical quenching concentration (*q_c_*). Compared with 3D ABX_3_ and double-perovskite A_2_B(I)B(III)X_6_ systems, A_3_BX_6_ generally exhibits a higher *q_c_*, typically around ~5 to 15 mol% for Ln^3+^ or transition-metal dopants, indicating a stronger tolerance toward heavy doping [32,58,59], which is higher than ABX_3_ and A_2_B(I)B(III)X_6_ [60,61]. Time-resolved PL measurements further elucidate the underlying dynamics. When the dopant concentration is below *q_c_*, the emission lifetime remains nearly unchanged, indicating that radiative recombination dominates the emission process. In contrast, once the dopant concentration exceeds *q_c_*, the average lifetime decreases markedly, suggesting the emergence of dopant-related nonradiative energy-loss pathways [62]. The concentration-quenching behavior in A_3_BX_6_ can be analyzed in terms of possible quenching mechanisms. Exchange interactions are generally negligible because of the relatively large separation between dopant ions, while the limited spectral overlap between emission and absorption bands excludes reabsorption as the dominant mechanism. Therefore, multipolar-interaction-assisted energy migration is considered the primary origin of quenching [63].

This process can be well described by the Yokota–Tanimoto model, which assumes that excitation energy migrates among luminescent centers and is eventually trapped by nonradiative quenching sites, leading to the reduction of emission intensity and lifetime [64]. The isolated [BX_6_]^3−^ octahedra in the vacancy-ordered A_3_BX_6_ framework further suppress long-range energy migration, accounting for the relatively delayed concentration quenching observed in this system [65]. In contrast, the continuously connected octahedral networks in ABX_3_ and A_2_B(I)B(III)X_6_ are more favorable for rapid energy migration, making concentration quenching more likely to occur at much lower dopant concentrations [65,66]. It is precisely this synergistic effect of structural isolation and strong exciton localization that enables A_3_BX_6_ to maintain relatively high radiative efficiency at elevated dopant levels and to exhibit markedly delayed concentration quenching behavior.

## The Potential Application of A_3_BX_6_ MHPs

### Stability and application potential

To evaluate the practical potential of A_3_BX_6_ materials, their stability under various environments, including moisture, high temperature, and high-energy irradiation, has been systematically examined. Under humid conditions, some A_3_BX_6_ compounds undergo structural phase transitions; however, unlike the typically irreversible transformations observed in ABX_3_ perovskites, these transitions are often reversible and can be restored by treatment with polar solvents. The associated changes in PL enable potential applications in moisture sensing, anticounterfeiting, and information encryption.

Owing to their relatively rigid lattices, A_3_BX_6_ compounds generally exhibit excellent resistance to thermal quenching. For instance, Rb_3_InCl_6_:Sb^3+^ maintains stable emission up to 500 K [67], while Cs_3_GdCl_6_:Sb^3+^ retains 82.4% of its room temperature (RT) emission intensity at 150 °C [22]. This behavior originates from the 0D isolated [BX_6_]^3−^ octahedral framework, in which thermal expansion is mainly accommodated by interoctahedral spaces and the relatively soft A-site sublattice, thereby preserving the local coordination environment of the emissive centers. Moreover, relatively high phonon energies and thermal activation barriers (e.g., 33.0-meV phonon energy and 51.3-meV activation energy for Cs_3_GdCl_6_ [22]) further suppress nonradiative relaxation processes.

In addition, the isolated octahedral framework effectively limits the diffusion of irradiation-induced defects, while the rigid lattice and localized excitonic emission enhance defect tolerance. As a result, A_3_BX_6_ materials display remarkable radiation stability. For example, Rb_3_InCl_6_:Sb^3+^ shows nearly unchanged RL intensity up to a cumulative dose of 561.6 Gy [68], whereas Cs_2_NaLuCl_6_/Cs_3_LuCl_6_:Sb^3+^ maintains stable RL output even at doses as high as 1,000 Gy [69], highlighting their robustness in high-energy radiation environments.

### Applications of photoexcitation and electrical excitation

Building upon the abovementioned optical and electronic advantages, such as reversible phase transitions, tunable luminescence, and B-site elemental diversity, A_3_BX_6_ MHPs exhibit great potential in a range of PL and EL applications. In addition, the structural isolation of A_3_BX_6_ enhances exciton binding energy, effectively suppressing exciton dissociation and free carrier generation at RT [70]. Moreover, the flat conduction band (CB) and valence band (VB) for A_3_BX_6_ MHPs lead to large carrier effective mass, further promoting exciton self-trapping and broadband luminescence efficiency [71–73]. These features render A_3_BX_6_ MHPs particularly suitable for use as phosphors and active layer in thin-film LED devices.

Under UV excitation, the photogenerated carriers formed in A_3_BX_6_ MHPs can be driven by an external electric field to convert optical signals into electrical signals, enabling their application in photodetectors. For photodetectors, detectivity is a key performance parameter (Table 1), which is expressed as Eq. (4) [15]:

$$D=\frac{R}{\sqrt{2{\mathrm{qI}}_{\text{dark}}}}$$ (4)

where *D* represents the detectivity, *R* represents the responsivity, *q* represents the elementary charge, and *I*_dark_ represents the dark current per unit area. Currently, the highest sensitivity of detectors based on A_3_BX_6_ MHPs has reached 1.48 × 10^13^ Jones [15].

**Table 1.** The key performance metrics of A_3_BX_6_ MHPs for photodetectors

Compound	Detectivity (Jones)	Responsivity	Dynamic range (dB)	Rise time	Fall time	Ref.
Cs_3_BiBr_6_	0.8 × 10^9^	25 mA/W	–	0 ms	60 ms	[123]
Cs_3_BiBr_6_/Cs_3_BiBr_9_	8.5 × 10^10^ (360 nm)	5.6 mA/W (360 nm)	>36.9 (360 nm)	1.4 μs	2.0 μs	[127]
	1.0 × 10^9^ (450 nm)	0.6 mA/W (450 nm)	>34.2 (450 nm)			
Cs_3_BiCl_6_/GaN	1.23 × 10^12^	0.26 A/W	123	28 μs	190 μs	[128]
Cs_3_CeBr_6_	1.48 × 10^13^	2.05 A/W	99.64	93 ms	345 ms	[15]
Cs_3_CeCl_6_:Gd^3+^	7.938 × 10^11^	0.195 A/W	–	0.30 s	0.35 s	[155]

MHPs, metal halide perovskites; Ref., reference.

Furthermore, the photogenerated carriers yield an emission wavelength spanning from 300 to 1,000 nm without the application of an external electric field. PL quantum yield (PLQY) is a key parameter for evaluating emissive efficiency, as shown in Eq. (5):

$$\text{PLQY}=\frac{N_{\mathrm{em}}}{N_{\mathrm{abs}}}$$ (5)

where *N*_em_ and *N*_abs_ represent the number of emitted and absorbed photons, respectively. Although visible-emissive A_3_BX_6_ MHPs can achieve PLQY approaching 100% [74] and even reach 93.2% in nanocrystals (NCs) [50]. The PLQY of NIR-emissive A_3_BX_6_ can reach 87.4%.

Relatively, the reversible hydration/dehydration between A_3_BX_6_ and A_2_BX_5_·H_2_O has been actively explored for application in NIR emission, information storage, and anticounterfeiting. Moreover, the phase transitions in A_3_BX_6_ compounds often involve changes in crystal symmetry and optical properties, enabling tunable emission color or intensity. Notably, the NIR-emissive PLQY of A_3_BX_6_ MHPs after phase transitions has been reported to exceed 75% [75].

In contrast, EL differs in that it generates photons through the injection and recombination of electric-field-driven electron–hole pairs. In EL devices, external quantum efficiency (EQE) is a critical parameter (Table 2), given by Eq. (6) [76]:

$$\mathrm{EQE}=\eta_{\text{out}}\cdot \eta_{r}\cdot \gamma \cdot \text{PLQY}$$ (6)

where *η*_out_ is the light outcoupling efficiency, the *η*_r_ is radiative recombination efficiency, and *γ* is exciton formation efficiency. As the active layers in thin-film LEDs, A_3_BX_6_ MHPs have demonstrated that a maximum EQE can reach 7.9%, which is the highest efficiency among metal halide deep-blue LEDs so far [13,14].

**Table 2.** The key performance parameters of A_3_BX_6_ MHPs for LEDs, including EL peak, Commission Internationale de l’Éclairage (CIE) coordinates, EQE, maximum luminance (*L*_max_), and half-lifetime (*T*_50_)

Device structure	EL peak (nm)	CIE coordinates	EQE (%)	*L*_max_ (cd/m^2^)	*T*_50_	Ref.
Al/TPBi/EHC-Cs_3_LaCl_6_/PEDO/ITO	442	(0.210, 0.171)	0.53	127	–	[150]
ITO/ZnO/Cs_3_La_0.17_Nd_0.83_Br_6_/TCTA/MoO_3_/Al	1,070, 1,350	–	3.49	–	488 min	[144]
ITO/PEDOT/PSS/PVK/Cs_3_CeBr_6_/SPPO13/LiF/Al	392, 430	(0.158, 0.02)	0.44	–	–	[185]
ITO/ZnO/Al_2_O_3_/Cs_3_CeBr_6_/TCTA/TAPC/HAT-CN/Al	391, 421	–	0.46	–	–	[73]
ITO/ZnO:PEI/Al_2_O_3_/Cs_3_CeI_6_/MCP/TAPC/TAPC:HAT-CN/HAT-CN/Al	430, 470	(0.15, 0.04)	3.5	470	45 min	[13]
ITO/ZnO/Al_2_O_3_/Rb_3_CeI_6_/MCP/TAPC/HAT-CN/Al	427, 468	(0.15, 0.06)	0.67	98	–	[186]
ITO/ZnO:PEI/Al_2_O_3_/Cs_3_CeI_6_/MCP/TAPC/TAPC:HAT-CN/HAT-CN/Al	435, 480	(0.145, 0.057)	7.9	1,075	72 min	[14]
Al/TPBi/EHC-Cs_3_EuCl_6_/PEDO/ITO	616	(0.648, 0.338)	5.17	370	440 h	[150]
Al/TPBi/EHC-Cs_3_TbCl_6_/PEDO/ITO	545	(0.267, 0.418)	1.58	2,373	–	[150]
ITO/ZnO/LiF/Cs_3_DyI_6_:Er^3+^/TPPO/TCTA/MoO_3_/Ag	1,540	(0.25, 0.35)	2.76	–	345 min	[187]

MHPs, metal halide perovskites; LED, light-emitting diode; EQE, external quantum efficiency; EL, electroluminescence.

### Application of x-ray excitation

In scintillators, the majority of light emission originates from electronic transitions induced by energetic electron impact, which, in some respects, resembles the electrical excitation processes in LEDs [30]. Consequently, high-performance x-ray detector materials must simultaneously possess a high effective atomic number (*Z*_eff_) and mass attenuation coefficient (*μ*/*ρ*) to ensure efficient x-ray absorption within limited thicknesses [77]. The total *μ*/*ρ* can be described by Eq. (7) [78]:

$$\mu /\rho =\tau /\rho +\sigma /\rho +\kappa /\rho +\sigma_{R}/\rho$$ (7)

where *τ/ρ* is the contribution of the photoelectric effect, σ*/ρ* is that of the Compton effect, *κ/ρ* is that of pair production, and *σ*_R_/*ρ* is that of Rayleigh scattering.

In the photoelectric effect regime (<100 keV), the mass attenuation coefficient approximately scales as Eq. (8) [79]:

$$\mu /\rho \propto \frac{Z_{\mathrm{eff}}^{n}}{E^{3}}$$ (8)

where *n* ranges from 3 to 4, indicating a strong dependence on atomic composition and photon energy. A_3_BX_6_ MHPs inherently possess the high *Z*_eff_ and *μ/ρ* due to the presence of heavy elements at both A- and X-sites, as well as their tunable composition through B-site doping.

In addition, 3 key performance metrics for x-ray detectors include L.Y., limit of detection (LoD), and spatial resolution (*f*) (Table 3). The L.Y. is defined as Eq. (9) [80]:

$$L.Y.=\frac{\eta \cdot E_{\mathrm{dep}}}{W}$$ (9)

where *η* is the energy conversion efficiency, *E*_dep_ is the energy deposited by x-rays, and *W* is the average energy required to generate one photon. Recently, the reported A_3_BX_6_ have demonstrated a value of L.Y. as high as 88,800 ph/MeV under 50-keV x-ray excitation [31], significantly outperforming conventional scintillators, such as NaI:Tl (~41,000 ph/MeV) and CsI:Tl (~60,000 ph/MeV) [81].

**Table 3.** The key performance metrics of A_3_BX_6_ MHPs for x-ray imaging

Compound	Sample	RL peak (nm)	Tube voltage (kV)	L.Y. (ph/MeV)	LoD (μGy/s)	Film thickness (μm)	*f* (lp/mm)	MTF	Ref.
Rb_3_InCl_6_:Sb^3+^	NC	510	30	15,500	5.20 × 10^3^	200	18.5	0.2	[68]
Cs_3_YCl_6_:Sb^3+^	Powder	550	70	16,000	1.12	500	9	–	[72]
Cs_3_CeCl_6_	Film	375, 410	35	7,533	18.3	7.4	10.2	0.2	[157]
Cs_3_CeBr_6_	Film	390, 425	35	28,510	1.1	7.1	26.4	0.2	[157]
Cs_3_EuCl_6_	Powder	592, 614	50	12,877	643.1 × 10^−3^	300	9.17	0.2	[158]
Rb_3_TbCl_6_	Polycrystal	548	80	88,800	115.38 × 10^−3^	260	3.9	0.2	[31]
Cs_3_TbCl_6_	Polycrystal	548	80	56,800	149.65 × 10^−3^	260	3.3	0.2	[31]
Cs_3_TbCl_6_:Sb^3+^	NC	548	50	20,300	212 × 10^−3^	–	9.6	0.2	[16]
Cs_3_TbCl_6_	Powder	547	70	51,800	63 × 10^−3^	1,000	12	–	[83]
Bzmim_3_TbCl_6_	Glass	547	40	4,101	308.30 × 10^−3^	1,000	25	–	[172]
Bzmim_3_EuCl_6_	Glass	592, 611	40	1,419	1.56	1,000	–	–	[172]
BuTPP_3_TbCl_6_	Glass	547	50	8,100	0.93	–	26.8	0.2	[173]
Cs_3_LuCl_6_/Cs_2_NaLuCl_6_:Sb^3+^	NC	–	50	–	140 × 10^−3^	200	14	0.2	[69]
Cs_3_LuCl_6_:Sb^3+^	NC	531	50	7,300	0.4	110	11.5	0.2	[167]
Cs_3_LuCl_6_:Ce^3+^	Powder	380, 420		8,120	57.9 × 10^−3^	100	8.38	0.2	[188]
Cs_3_LuCl_6_:Tb^3+^	Powder	548	50	21,000	–	200	11 (423 K)	0.2	[189]
Cs_3_LuCl_6_:Ce^3+^, Sm^3+^	Powder	604	50	7,600	–	200	8 (423 K)	0.2	[189]

MHPs, metal halide perovskites; NC, nanocrystal; RL, radioluminescence; MTF, modulation transfer function; L.Y., light yield.

A high L.Y. enhances signal output under low x-ray dose, improving the signal-to-noise ratio (SNR) and contributing to superior detection sensitivity. For estimating the practical sensitivity, LoD is defined as Eq. (10) [82]:

$$\mathrm{LoD}=\frac{3\times \sigma_{\mathrm{bg}}}{S}$$ (10)

where *σ*_bg_ is the standard deviation of background noise and *S* is the slope of the signal response to x-ray dose. As is known, LoD as low as 63 nGy/s has been reported, highlighting the excellent detection sensitivity of A_3_BX_6_ scintillators [83].

In general, a combination of high L.Y. and low LoD is highly desirable for low-dose x-ray imaging applications. As for spatial resolution, it is commonly evaluated using the modulation transfer function (MTF), defined as Eq. (11) [84]:

$$\mathrm{MTF}\left(f\right)=\frac{M_{\text{output}}\left(f\right)}{M_{\text{input}}\left(f\right)}$$ (11)

where *M*_output_(*f*) and *M*_input_(*f*) represent the contrast of the output and input signals, respectively, at a given spatial frequency. All inorganic A_3_BX_6_ MHPs have demonstrated the spatial frequency of 26.4 lp/mm at an MTF of 0.2. This recorded *f* value of A_3_BX_6_ MHP demonstrates greater potential than that of current commercial scintillators [68].

### The application of γ-ray excitation

Beyond x-ray applications, A_3_BX_6_ MHPs also demonstrate considerable potential in γ-ray detection. The total *μ*/*ρ* for γ-ray photons is described by the same physical formalism as that for x-ray photons. Relative to x-rays with photoelectric effect, Compton scattering and pair production become increasingly dominant at γ-ray energies (>100 keV). Therefore, as candidates of γ-ray scintillators, the modified A_3_BX_6_ MHPs require not only high ρ and *Z*_eff_ but also high L.Y., fast response, and superior E.R. (Table 4).

**Table 4.** The key metrics of A_3_BX_6_ MHPs single crystals (SCs) for γ-ray scintillator

Compound	Crystal size	Density (g/cm^3^)	γ-Ray source	L.Y. (ph/MeV)	Decay time (ns)	E.R. (%)	Ref.
Cs_3_BiCl_6_	–	–	^137^Cs	800	0.61, 9.4	–	[125]
Cs_3_ScCl_6_:0.5% Ce^3+^	Φ ~ 6 × 2 mm	3.32	^137^Cs	9,200	3.6, 54, 963	10	[135]
Li_3_YCl_6_:Ce^3+^	Φ ~ 4 mm	2.45	^137^Cs	6,185	250, 2,300	–	[146]
Cs_3_LaCl_6_:20% Ce^3+^	1 × 2 × 2 mm^3^	3.27	^137^Cs	2,000	56, 380	–	[34]
Cs_3_LaCl_6_:40% Ce^3+^	1 × 2 × 2 mm^3^	3.27	^137^Cs	–	54, 314	9	[34]
Cs_3_LaBr_6_:10% Ce^3+^	1 × 2 × 2 mm^3^	3.99	^137^Cs	35,000	71, 437	–	[34]
Cs_3_LaBr_6_:40% Ce^3+^	1 × 2 × 2 mm^3^	3.99	^137^Cs	–	37, 287	8	[34]
Cs_3_LaCl_6_:8% Ce^3+^	3 × 3 × 1 mm^3^	3.36	^137^Cs	16,000	79, 291	8.6	[58]
Cs_3_LaBr_6_:15% Ce^3+^	3 × 3 × 1 mm^3^	3.82	^137^Cs	32,500	44, 124	4.9	[58]
Cs_3_PrCl_6_	3.0 × 3.0 × 1.0 mm	–	^137^Cs	5,900	1.5, 11.7, 72	–	[163]
Cs_3_CeCl_6_	>1 cm^3^	3.4	^137^Cs	21,000	50, 300	7.6	[160]
Cs_3_CeCl_6_	Φ 10 × 30 mm	3.4	^137^Cs	19,000	50, 300	8.4	[17]
Cs_3_GdCl_6_:Ce^3+^	4–10 mm^3^	3.56	^137^Cs	24,500	39, 129	4.5	[32]
Cs_3_GdBr_6_:Ce^3+^	4–10 mm^3^	4.14	^137^Cs	47,000	72, 270	4	[32]
(Gua)_3_SbCl_6_	8 × 5 × 3 mm^3^	–	^57^Co	2,000	2,500	–	[121]

MHPs, metal halide perovskites; L.Y., light yield; E.R., energy resolution.

The E.R. is a critical parameter for evaluating a scintillator’s ability to discriminate between γ-ray energies, and its value is defined as Eq. (12):

$$E.R.=\frac{\text{FWHM}}{E_{\gamma }}\times 100\%$$ (12)

where FWHM is the full width at half maximum of the full-energy peak and *E*_γ_ is the energy of the incident γ-ray. A smaller E.R. value corresponds to superior detector performance and enables more accurate identification of γ-ray sources [85]. Up to now, the maximum L.Y. of A_3_BX_6_ scintillators has achieved 47,000 ph/MeV under ^137^Cs γ-ray excitation with a good E.R. of 4.0% [32]. Compared to commercial scintillators (Table 5), their performance still differs from that of commercial scintillators.

**Table 5.** The key performance parameters of commercial scintillators [LYSO = (Lu, Y)_2_SiO_5_]

Compound	Crystal size	L.Y. (ph/MeV)	E.R. (%)	Decay time (ns)	Proportionality	Afterglow	Hygroscopicity	Radiation Hardness	Ref.
NaI:Tl^+^	Φ150 × 400 mm	38,000	6–7	230	Medium	Noticeable slow component	Hygroscopic	Poor–moderate	[190,191]
LaBr_3_:Ce^3+^	89 × 203 mm	61,000	2.7–3.2	16–25	Good	Very low	Highly hygroscopic	Moderate–good	[192,193]
SrI_2_:Eu^2+^	1–2 in	90,000	2.6–3.0	1–1.2 × 10^3^	Excellent	Low	Highly hygroscopic	Moderate	[194,195]
LYSO:Ce^3+^	64 × 220 mm	39,900	8.2	40–42	Moderate	Low	Nonhygroscopic	Excellent	[196,197]

L.Y., light yield; E.R., energy resolution.

In general, A_3_BX_6_ MHPs feature flexible B-site composition, tunable luminescence, and good scintillation performance under high-energy excitation. These attributes endow them with great advantages for multifunctional applications in PL/EL devices, as well as x-ray or γ-ray detections, highlighting their potential as a new generation of luminescent scintillators enabled by B-site compositional engineering.

## The Categories of Cation-Engineered A_3_BX_6_ MHPs

By engineering host and dopant cation, the radiative transition mechanisms of A_3_BX_6_ MHPs can be tailored, enabling diverse carrier dynamics and performance to meet the demands of potential applications in PL, EL, and high-energy photon detection. In this section, we focus on 6 categories of A_3_BX_6_ MHPs classified on the basis of B-site cation engineering and highlight their corresponding luminescence mechanisms and applications.

### A_3_InX_6_ type MHPs

**Fig. 3.**
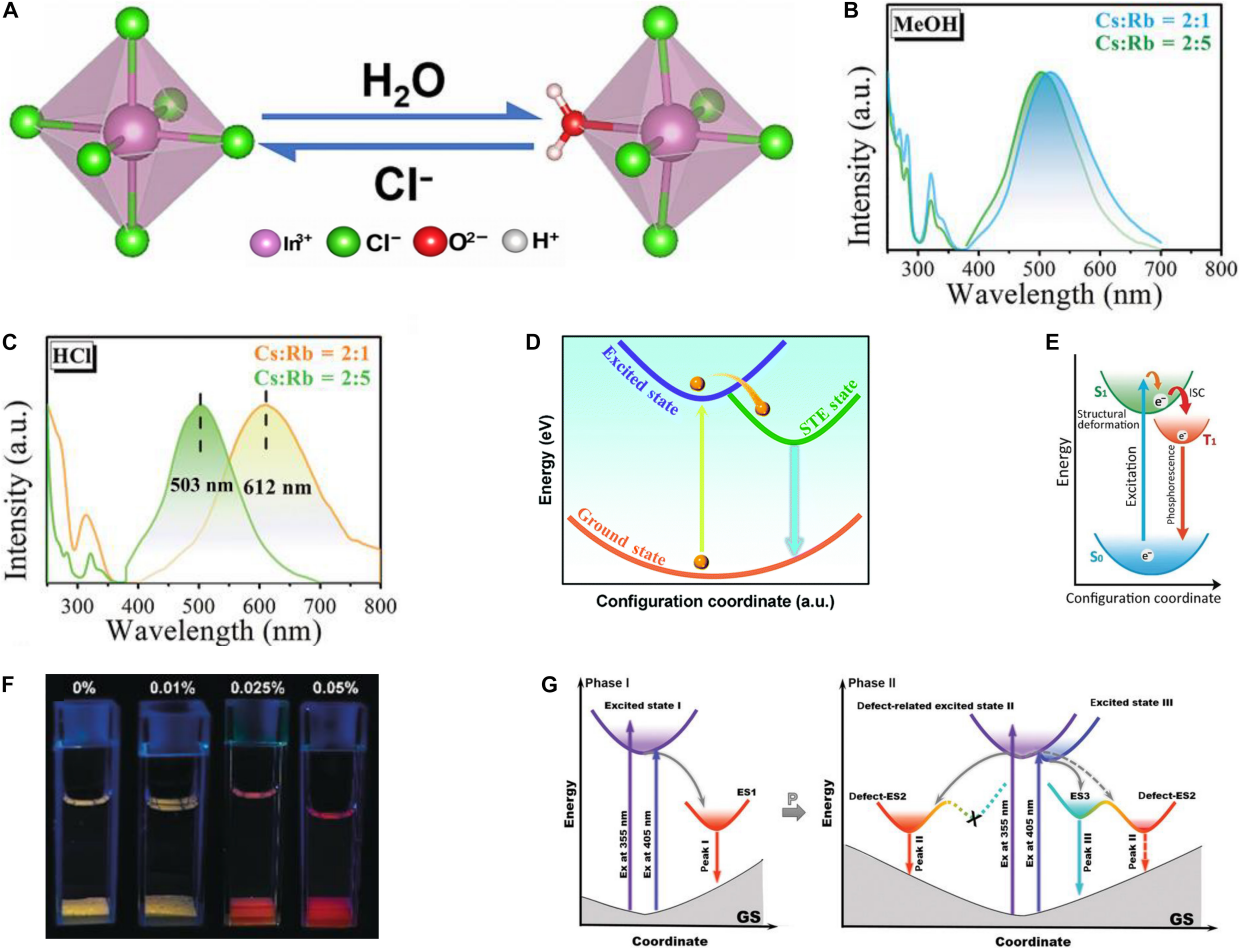
(A) The transformation between a regular octahedron [InCl_6_]^3−^ and distorted octahedron [InX_5_OH_2_]^2−^. Adapted with permission [87]. Copyright 2020, published by Wiley-VCH. (B) The photoluminescence (PL) and PL excitation (PLE) of pure A_3_InCl_6_ synthesized from methanol. MeOH, methanol. Adapted with permission [89]. Copyright 2021, published by Wiley-VCH. (C) The PL and PLE of the coexisting samples of A_3_InCl_6_ and A_2_InX_5_·H_2_O were synthesized using hydrochloric acid. Adapted with permission [89]. Copyright 2021, published by Wiley-VCH. (D) Schematic of the photophysical processes in Cs_3_InBr_6_. Adapted with permission [71]. Copyright 2021, published by Royal Society of Chemistry. (E) Configurational coordinate diagram illustrating the origin of PL in Cs_2_InBr_5_·H_2_O. ISC, intersystem crossing. Adapted with permission [78]. Copyright 2019, published by Wiley-VCH. (F) The dehydrated mixture is contained in the tetrahydrofuran solution. The luminescence phenomenon of different water volumes (0 to 0.05%, v/v). Adapted with permission [78]. Copyright 2019, published by Wiley-VCH. (G) Schematic illustrations of the relaxation mechanisms of the self-trapped excitons (STEs) in phase I (left) and phase II (right). Ex, excitation; GS, ground state. Adapted with permission [23]. Copyright 2021, published by Wiley-VCH.

As one of the P-block cations, In^3+^ has emerged as a promising candidate for B-site host composition in A_3_BX_6_ (i.e., A_3_InX_6_) MHPs because In^3+^ exhibits good chemical stability and structural compatibility, a tunable electronic structure, and unique luminescent properties (such as intrinsic luminescence, water-driven luminescence, and pressure-driven luminescence) [23,71,86]. The A-site cations in the majority of A_3_InX_6_ MHPs are predominantly composed of the inorganic alkali metals, such as Cs^+^, K^+^, and Rb^+^ (Table 6). Notably, a water-driven strategy effectively induces intrinsic luminescence by incorporating H_2_O into the lattice, converting A_3_InX_6_ into A_2_InX_5_·H_2_O (Fig. 3A) [21,87,88]. However, such a phase transition may lead to structural instability and disrupt the electronic band structure, ultimately resulting in degraded device performance or even device failure. To manage the structural transformation of A_3_InX_6_ MHPs, synthesis conditions must be carefully controlled in humid environments with a polar solvent. For instance, using methanol favors the formation of pure A_3_InX_6_, whereas hydrochloric acid promotes the coexistence of A_3_InX_6_ and A_2_InX_5_·H_2_O (Fig. 3B and C) [89]. Upon hydration, the [InX_6_]^3−^ octahedral framework evolves into low-symmetry [InX_5_OH_2_]^2−^ dramatically modifying carrier recombination and intrinsic luminescence [87]. For example, Cs_3_InBr_6_, which exhibits STE emission (Fig. 3D) [71,90], transforms to Cs_2_InBr_5_·H_2_O with RT phosphorescence (Fig. 3E), allowing reversible water sensing: The dehydrated powder exhibits yellow luminescence when dispersed in tetrahydrofuran, with its emission undergoing a distinct chromatic transition to red upon reaching a water content of 0.025% (v/v) (Fig. 3F) [78]. Under high pressure, the lower-symmetry [InBr_5_OH_2_]^2−^ octahedra undergo a secondary phase transition that activates mechanoluminescence (Fig. 3G) [23]. Together, the water- and pressure-driven effects in A_3_InX_6_ MHPs endow their intrinsic luminescence with multifunctionality.

**Table 6.** The key PL properties of A_3_InX_6_ (including PL peak, FWHM, Stokes shift, decay time and PLQY)

Compound	Sample	PL peak (nm)	FWHM (nm)	Stokes shift (nm)	Decay time	PLQY at *λ*_ex_	Ref.
Cs_3_InCl_6_	SC	434	88	154	6.85 μs	8% (*λ*_ex_ = 280 nm)	[59]
Cs_3_InBr_6_	NC	450	–	75	8.25 ns	22.3% (*λ*_ex_ = 375 nm)	[90]
					55.59 ns		
Cs_3_InCl_6_	NC	397	98	107	–	24% (*λ*_ex_ = 370 nm)	[71]
Cs_3_In(Cl_0.5_Br_0.5_)_6_	NC	424	89	113	–	6% (*λ*_ex_ = 370 nm)	[71]
Cs_3_InBr_6_	NC	440	80	70	–	46% (*λ*_ex_ = 370 nm)	[71]
Cs_3_In(Br_0.5_I_0.5_)_6_	NC	507	87	103	–	2% (*λ*_ex_ = 370 nm)	[71]
Cs_3_InI_6_	NC	535	87	75	–	16% (*λ*_ex_ = 370 nm)	[71]
Rb_3_InCl_6_:Sb^3+^	SC	497	–	127	288.5 ns	95% (*λ*_ex_ = 324 nm)	[87]
					1.82 μs		
Cs_3_InCl_6_:Sb^3+^	NC	510	–	170	1.98 μs	93.2% (*λ*_ex_ = 340 nm)	[50]
Rb_3_InCl_6_:Sb^3+^	NC	510	107	230	1.65 μs	68.01% (*λ*_ex_ = 320 nm)	[68]
K_3_InCl_6_:Sb^3+^	SC	505	–	163	4.12 μs (100 K)	–	[97]
					4.46 μs (200 K)		
					4.17 μs (400 K)		
K_3_InCl_6_:Sb^3+^	SC	555	–	213	4.69 μs (100 K)	–	[97]
					4.52 μs (400 K)		
Rb_3_InCl_6_:Sb^3+^	SC	521	112	146	12.5 μs	>90% (*λ*_ex_ = 375 nm)	[67]
Cs_3_InCl_6_:Sb^3+^	NC	512		172	2.07 μs	52.3% (*λ*_ex_ = 322 nm)	[21]
Cs_3_InCl_6_:Cu^+^	SC	398	54	118	13.95 μs	95% (*λ*_ex_ = 280 nm)	[59]
Cs_3_InCl_6_:Sb^3+^/Mn^2+^	NC	530, 620	–	195, 295	1.61 μs	51.38% (*λ*_ex_ = 325 nm)	[102]

PL, photoluminescence; SC, single crystal; NC, nanocrystal; PLQY, PL quantum yield; FWHM, full width at half maximum.

**Fig. 4.**
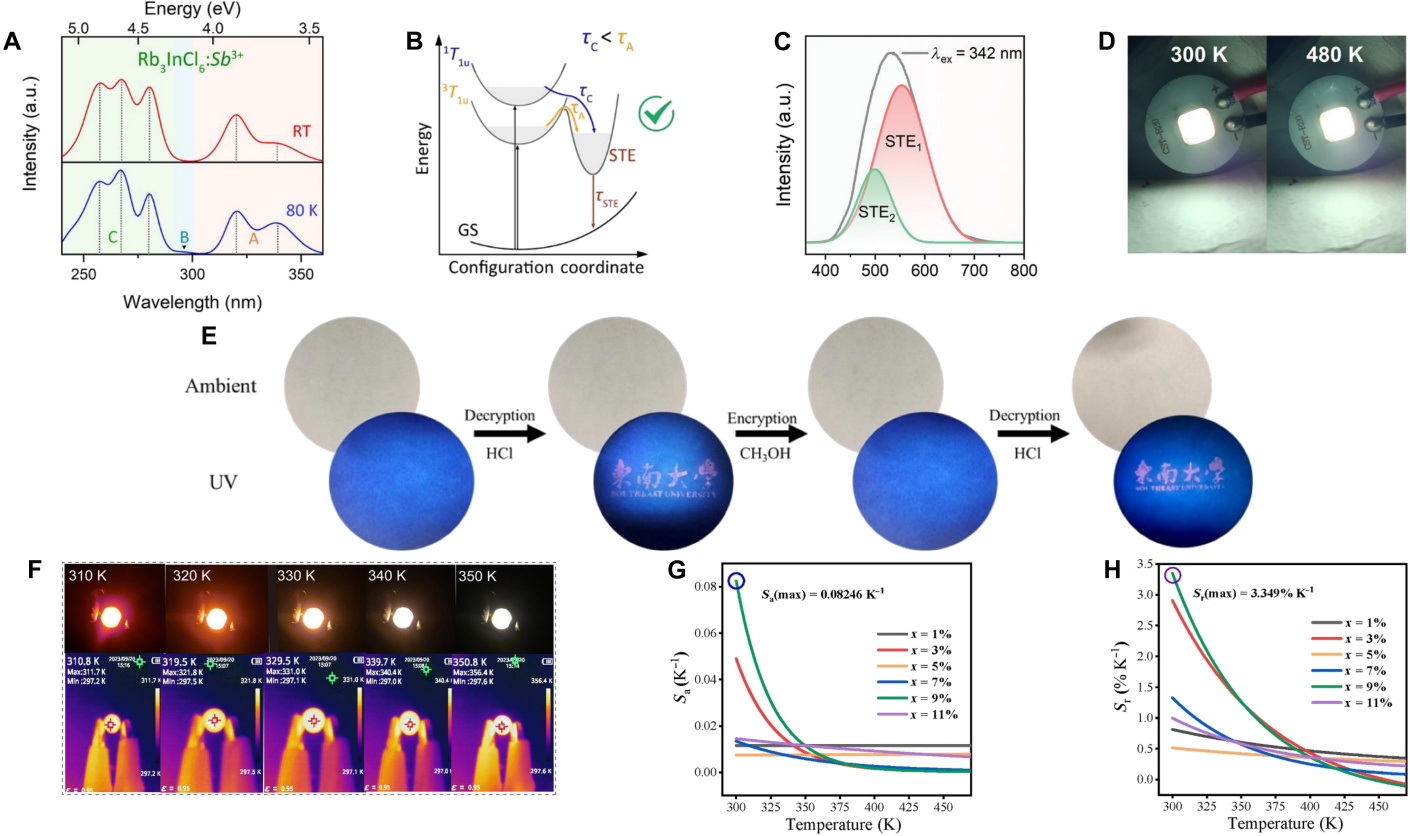
(A) Photoluminescence (PL) excitation (PLE) spectra of Rb_3_InCl_6_:Sb^3+^ collected at room temperature (RT) and 80 K. Adapted with permission [96]. Copyright 2022, published by American Chemical Society. (B) The photophysical process of Rb_3_InCl_6_:Sb^3+^. GS, ground state. Adapted with permission [96]. Copyright 2022, published by American Chemical Society. (C) Peak-fitted PL spectra of K_3_InCl_6_:Sb^3+^. Adapted with permission [97]. Copyright 2024, published by Wiley-VCH. (D) Photographs of the white light-emitting diode (LED) based on Rb_3_InCl_6_:Sb^3+^ at low current (left) and high current (right). Adapted with permission [67]. Copyright 2024, published by American Chemical Society. (E) Reversible fluorescence switching between Cs_2_InCl_5_·H_2_O:Te^4+^ and Cs_3_InCl_6_:Te^4+^. Adapted with permission [88]. Copyright 2023, published by Academic Press Inc. (F) The LED color changes as the temperature is monitored from 300 to 350 K using a temperature gun. Adapted with permission [92]. Copyright 2025, published by Royal Society of Chemistry. (G) Corresponding absolute sensitivity (*S*_a_) and (H) relative sensitivity (*S*_r_) values of Cs_2_InCl_5_·H_2_O:xBi^3+^. Adapted with permission [93]. Copyright 2024, published by Elsevier.

Although the intrinsic luminescence for A_3_InX_6_ MHPs supports good PL applications, their PLQY remains low, primarily due to the parity-forbidden transition [91]. To improve this, as described in the “Dopant-related luminescence” section, researchers have incorporated ns^2^-typed dopants into A_3_InX_6_ hosts. Most efforts have focused on Sb^3+^, Bi^3+^, and Te^4+^, which introduce new electronic states that break the forbidden transition and enhance PL properties [87,92,93]. Upon Sb^3+^ incorporation, A_3_InX_6_:Sb^3+^ exhibits 3 different absorption bands: the ^1^S_0_ → ^3^P_1_, ^1^S_0_ → ^3^P_2_, and ^1^S_0_ → ^1^P_1_ (Fig. 4A) [94,95]. The emission for Cs_3_InCl_6_:Sb^3+^ has been ascribed to the STE state based on the excited-state absorption of Sb^3+^ from ^3^P_1_ to higher-energy states by femtosecond transient absorption spectra [21]. Although the ^1^S_0_ → ^3^P_1_ and ^1^S_0_ → ^1^P_1_ transitions exhibit distinct excitation energies, the excitons generated by both processes ultimately relax into the same STE state, resulting in emission (Fig. 4B) [96]. According to the similar luminescence mechanism, K_3_InCl_6_:Sb^3+^ displays dual emission peaks at 505 and 555 nm, with distinct decay lifetimes observed for these 2 emission bands, indicating the existence of 2 STE emission (Fig. 4C) [97]. In addition, Sb^3+^ imparts high resistance to thermal quenching to the material, and the Rb_3_InCl_6_:Sb^3+^ is applied for high-temperature white LED with exceptional thermal quenching resistance up to 500 K (Fig. 4D) [67].

In contrast to the bright emission observed in A_3_InX_6_:Sb^3+^ MHPs, Cs_3_InCl_6_:Te^4+^ remains negligible PL at RT [88]. However, the Te^4+^ doping strategy of water-driven phase transition induces bright-yellow luminescence in A_2_InCl_5_·H_2_O (A = Cs^+^ and Rb^+^) [88,92,98,99]. One application is a promising platform for anticounterfeiting with ethanol-sensitive structural transformation between Cs_2_InCl_5_·H_2_O:Te^4+^ and Cs_3_InCl_6_:Te^4+^ (Fig. 4E) [88]. The other application is visual temperature monitoring through the color-changing temperature indicator light in ratio mode, with luminescence color transition from orange–red to white (Fig. 4F) [92]. However, PL vanishes from RT to 480 K due to phase transition to Rb_3_In_2_Cl_9_, limiting its applicable temperature range [98]. The Bi^3+^ doping strategy in Cs_2_InCl_5_·H_2_O induces a phase transition similar to that observed with Te^4+^ doping, which extends the luminescent temperature window to 300 to 460 K (Fig. 4G and H) [93].

**Fig. 5.**
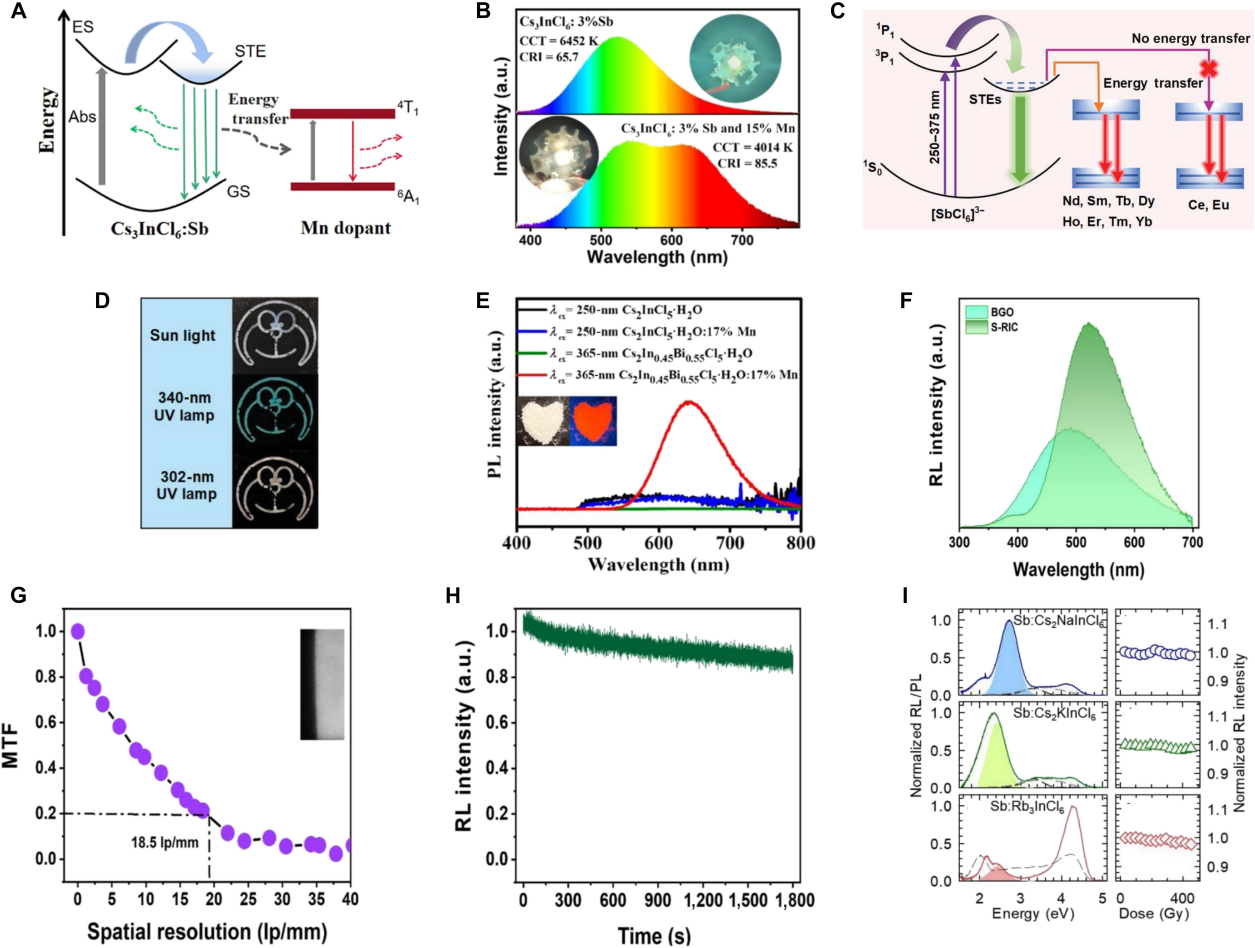
(A) Proposed photoluminescence (PL) mechanism of Sb^3+^/Mn^2+^-codoped Cs_3_InCl_6_ NCs. Adapted with permission [102]. GS, ground state; ES, excited state; Abs, absorption. Copyright 2024, published by American Chemical Society. (B) Emission spectra of Cs_3_InCl_6_:3% Sb^3+^ and Cs_3_InCl_6_:3% Sb^3+^, 15% Mn^2+^-based light-emitting diodes (LEDs) under 317-nm chip excitation. Adapted with permission [102]. Copyright 2024, published by American Chemical Society. (C) Schematic diagram of Cs_3_InCl_6_:Sb^3+^,Ln^3+^ (Ln^3+^ = Nd^3+^, Sm^3+^, Tb^3+^, Dy^3+^, Ho^3+^, Er^3+^, Tm^3+^, and Yb^3+^). Adapted with permission [50]. Copyright 2023, published by Wiley-VCH. (D) Images of security patterns made of Cs_3_InCl_6_:1% Sb^3+^,50% Eu^3+^ nanocrystals (NCs). Adapted with permission [50]. Copyright 2023, published by Wiley-VCH. (E) PL spectra and patterns for the Cs_2_InCl_5_·H_2_O:Bi^3+^,Mn^2+^. Adapted with permission [103]. Copyright 2023, published by American Chemical Society. (F) Radioluminescence (RL) spectra of Rb_3_InCl_6_:Sb^3+^ and Bi_4_Ge_3_O_12_ (BGO) under x-ray excitation. Adapted with permission [68]. Copyright 2025, published by Elsevier. (G) Modulation transfer function (MTF) curves of the film of Rb_3_InCl_6_:Sb^3+^ NC and polysulfone. Adapted with permission [68]. Copyright 2025, published by Elsevier. (H) RL intensity of Rb_3_InCl_6_:Sb^3+^ under continuous x-ray irradiation. Adapted with permission [68]. Copyright 2025, published by Elsevier. (I) The RL spectra of Rb_3_InCl_6_:Sb^3+^, Cs_2_KInCl_6_:Sb^3+^, and Cs_2_NaInCl_6_:Sb^3+^ with increasing cumulative dose up to 500 Gy. Adapted with permission [65]. Copyright 2021, published by American Chemical Society.

Beyond ns^2^-typed dopants act as sensitizers, transition-metal cations and Ln^3+^ are regarded as effective alternatives through ET process [20], the STE–transition metal ET is typically governed by Dexter exchange interaction [100], whereas STE–Ln^3+^ transfer mainly occurs via dipole–dipole interaction [101]. For example, in Cs_3_InCl_6_:Sb^3+^,Mn^2+^, ET from STEs to Mn^2+^ facilitates simultaneous green STE emission and Mn^2+^-derived red emission (Fig. 5A). The white LED with Cs_3_InCl_6_:Sb^3+^, Mn^2+^ achieves higher color rendering index (CRI = 85.5) and correlated color temperature (CCT = 6,452 K) than that of Cs_3_InCl_6_:Sb^3+^ (Fig. 5B) [102]. Similarly, Cs_3_InCl_6_:Sb^3+^,Ln^3+^ (Ln^3+^ = Nd^3+^, Sm^3+^, Tb^3+^, Dy^3+^, Ho^3+^, Er^3+^, Tm^3+^, and Yb^3+^) leverages ET from STE to Ln^3+^ to achieve NIR luminescence (Fig. 5C). Notably, Cs_3_InCl_6_:Sb^3+^,Nd^3+^ exhibits both green and NIR emissions, while Cs_3_InCl_6_:Sb^3+^,Eu^3+^ exhibits excitation-wavelength-dependent luminescence due to the absence of ET between STEs and Eu^3+^, enabling anticounterfeiting applications (Fig. 5D) [50]. In addition, introducing an ET pathway between Bi^3+^ and Mn^2+^ in Cs_2_InCl_5_·H_2_O:Bi^3+^,Mn^2+^ enhances its originally weak emission by 60-fold (Fig. 5E) [103]. These strategies highlight the synergistic potential of codoping and structural engineering to modulate luminescence in A_3_InX_6_ MHPs for multifunctional optoelectronic applications.

Apart from their application of PL and EL, A_3_InX_6_ MHPs have also been explored for scintillation applications. The emerging studies highlight their immense potential in this field. For instance, the L.Y. of Rb_3_InCl_6_:Sb^3+^ NCs exhibit 1.65 times higher than that of Bi_4_Ge_3_O_12_ (BGO) under x-ray excitation, reaching 15,500 ph/MeV (Fig. 5F). Composite films fabricated with polysulfone and Rb_3_InCl_6_:Sb^3+^ achieve a spatial resolution of 18.5 lp/mm at an MTF of 0.2, showcasing promise for bioimaging applications (Fig. 5G). Radiation resistance is another critical parameter for scintillators [68]. Rb_3_InCl_6_:Sb^3+^ retains 85% of its initial RL intensity after 30-min exposure to a dose rate of 5.2 mGy/s and maintains its initial RL intensity even at cumulative doses of 500 Gy_air_ under a high dose rate of 0.5 Gy/s (Fig. 5H and I) [65,68]. These results underscore the outstanding radiation hardness of In^3+^-based MHPs and their potential for x-ray imaging. However, their performance under γ-ray irradiation remains unexplored, necessitating further investigation to unlock their full capabilities in high-energy radiation detection. This pioneering work positions In^3+^-based MHPs as promising candidates for next-generation scintillators, bridging the gap between optical excellence and functional radiation detection.

### A_3_SbX_6_-type MHPs

**Fig. 6.**
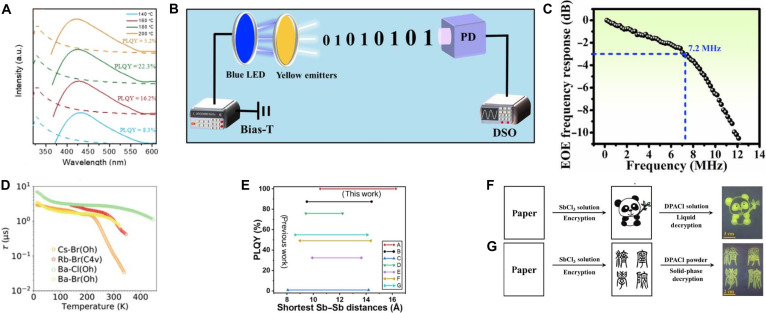
(A) Absorption and photoluminescence (PL) spectra of K_3_SbCl_6_ nanocrystals (NCs) at different synthesized temperatures. Adapted with permission [104]. Copyright 2021, published by Elsevier. (B) Electrical–optical–electrical (EOE) frequency response of the [Na(DMSO)_2_]_3_SbBr_6_-based optical converter [109]. DSO, digital storage oscilloscope; PD, photodetector. Copyright 2022, published by Wiley-VCH. (C) The EOE frequency response. Adapted with permission [109]. Copyright 2022, published by Wiley-VCH. (D) Temperature-dependent decay time for [C@Cs]_2_CsSbBr_6_ and its derivatives. Adapted with permission [110]. Copyright 2020, published by American Chemical Society. (E) Correlation between shortest Sb–Sb distances and PL quantum yield (PLQY) for 0D Sb^3+^-based halides. (Among them, A is (gua-DPG)_3_SbCl_6_ [74], B is [Bzmim]_3_SbCl_6_ [118], C is (PMA)_3_SbBr_6_ [112] , D is [DMPZ]_2_SbCl_6_·Cl·(H_2_O)_2_ [182], E is [Prmim]_3_SbCl_6_ [113], F is [Hmim]_3_SbCl_6_ [113], and G is H_3_SbBr_6_(L)_6_ [183].) Adapted with permission. Copyright 2023, published by American Chemical Society. The information encryption and decryption process for (F) panda pattern and (G) Chinese character, respectively. Adapted with permission [116]. Copyright 2022, published by Elsevier.

P-block metal ion Sb^3+^ can not only act as dopant in enhancing the luminescence in A_3_InX_6_ MHPs but also serve as the B-site host composition to construct A_3_SbX_6_ MHPs. However, all-inorganic A_3_SbX_6_ faces the challenge of low PL efficiency, primarily due to crystal defects, which act as multiple nonradiative recombination centers [104,105]. For instance, K_3_SbCl_6_ NCs exhibit a broad STE emission centered at 440 nm (FWHM = 102 nm); through the optimization of the synthesis temperature, the maximum PLQY reached 22.3% (Fig. 6A) [104]. However, the presence of defects leads to poor optical properties in materials synthesized at RT, resulting in limited research on all-inorganic A_3_SbX_6_ compounds.

Compared to all-inorganic A_3_SbX_6_ MHPs, implementation of the metal complexes as the A-site to construct A_3_SbX_6_ organic–inorganic hybrid perovskites (OIHPs) can substantially strengthen perovskite framework robustness and lattice coherence, thereby enhancing charge carrier mobility through optimized defect configurations and radiation hardness [106–108]. For example, [Na(DMSO)_2_]_3_SbBr_6_ demonstrates a broadband emission with a remarkable Stokes shift of 200 nm and a PLQY of 60%. The measured electrical–optical–electrical (EOE) frequency response yields a 7.2-MHz bandwidth, which enables an 81.6-Mbps data transmission rate using orthogonal frequency-division multiplexing (Fig. 6B and C) [109]. Similarly, [C@Cs]_2_CsSbBr_6_ (C = 18-crown-6) exhibits a broad orange emission (1.3 eV; FWHM = 0.35 eV) under 365-nm excitation. Its temperature-dependent PL lifetime yields a thermal sensitivity coefficient (*α*) of 0.04 °C^−1^, significantly surpassing conventional thermoluminescent materials (*α* = 0.005 to 0.03 °C^−1^) (Fig. 6D), highlighting its potential for high-sensitivity thermal imaging applications [110].

In addition, the incorporation of organic cations imparts structural flexibility and dynamic characteristics to the crystal lattice, providing enhanced freedom and pathways for exciton migration and recombination, thereby improving luminescence efficiency [111]. Notably, Sb^3+^-based OIHPs achieve a record PLQY of up to 100% (Fig. 6E) [74]. Relative to their inorganic Sb^3+^-based MHPs counterparts, Sb^3+^-based OIHPs exhibit broader Stokes shifts. For instance, [C_5_mim][Mim]_2_SbCl_6_ ([C_5_mim]^+^ = 1-pentyl-3-methylimidazolium; [Mim]^+^ = *N*-methylimidazolium) demonstrates a Stokes shift of 335 nm, significantly larger than those of other Sb^3+^-based OIHPs such as [primim]_3_SbCl_6_, [PMA]_3_SbBr_6_, and [DphgH]_3_SbCl_6_ ([primim] = 1-propyl-3-methylimidazolium, [PMA] = phenylmethylammonium, [Dphg] = *N*,*N′*-diphenylguanidine) [112–114]. This phenomenon arises from the disruption of supramolecular interactions within imidazolium-based groups, which triggers pronounced anionic lattice distortions [115]. Intriguingly, certain OIHPs exhibit stimulus-responsive luminescence, enabling advanced applications in information encryption. For example, [DPA]_3_SbCl_6_ (DPA = dipropylamine) enables a dual-mode encryption–decryption process mediated by ethanol exposure: Encrypted information remains invisible under ambient and UV light due to the inherent transparency of SbCl_3_, while decryption is achieved by spraying with a [DPA]Cl ethanol solution to generate fluorescent [DPA]_3_SbCl_6_ under UV illumination. Subsequent methanol treatment quenches the emission for re-encryption, followed by dissolution recrystallization to restore fluorescence for secondary decryption (Fig. 6F and G) [116].

**Fig. 7.**
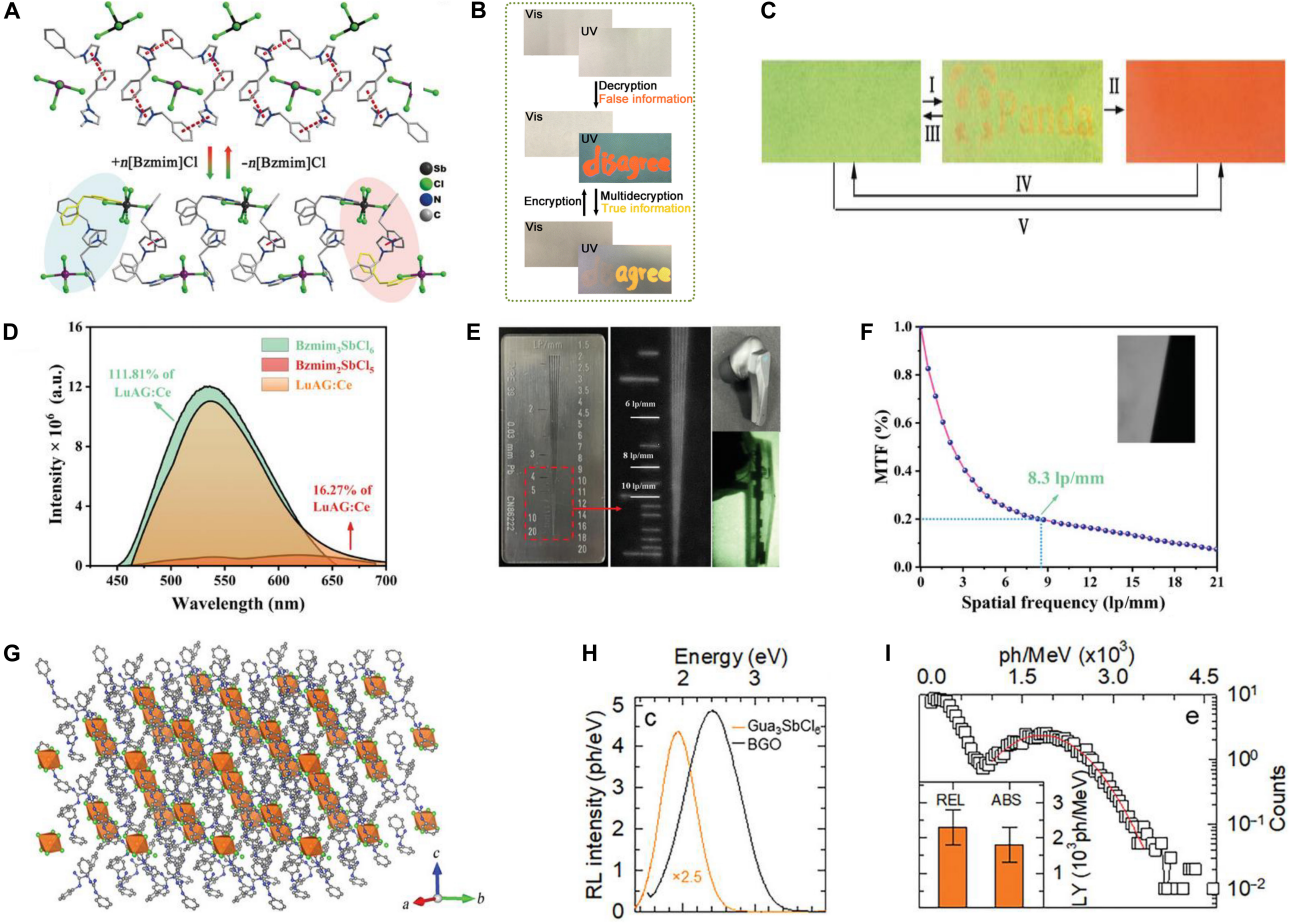
(A) Structural transformation between [Bzmim]_3_SbCl_6_ and [Bzmim]_2_SbCl_5_. Adapted with permission [118]. Copyright 2019, published by Wiley-VCH. (B) Digital photographs of the paper before and after heating under 390-nm ultraviolet (UV) light, with words written using [Bzmim]Cl and SbCl_3_ inks. Vis, visible. Adapted with permission [117]. Copyright 2025, published by Royal Society of Chemistry. (C) Schematic of the laser-based encryption/decryption process: (I) Printing the paper with a laser engraver; (II) blacking out in a moist environment; (III) aging at 353 K for initialization; (IV) heating at 353 K; and (V) keeping in moisture for 6 h [118]. Copyright 2019, published by Wiley-VCH. (D) The radioluminescence (RL) and light yield (L.Y.) of Bzmim_3_SbCl_6_. Adapted with permission [119]. Copyright 2024, published by Wiley-VCH. (E) The x-ray images of a standard resolution pattern plate and an earphone based on [Bzmim]_3_SbCl_6_@PMMA as the scintillator. Adapted with permission [119]. Copyright 2024, published by Wiley-VCH. (F) Modulation transfer function (MTF) curve of the [Bzmim]_3_SbCl_6_@PMMA scintillator film. Adapted with permission [119]. Copyright 2024, published by Wiley-VCH. (G) Configuration of Gua_3_SbCl_6_ supercell. Adapted with permission [121]. Copyright 2023, published by Wiley-VCH. (H) RL spectra of Gua_3_SbCl_6_ and Bi_4_Ge_3_O_12_ (BGO). Adapted with permission [121]. Copyright 2023, published by Wiley-VCH. (I) L.Y. of Gua_3_SbCl_6_ under ^57^Co γ-ray excitation. Adapted with permission [121]. REL, relative emission intensity; ABS, absorption. Copyright 2023, published by Wiley-VCH.

While most organic A-site cations primarily enhance luminescence efficiency by introducing structural flexibility and modulating carrier recombination without altering the intrinsic luminescence mechanism, certain organic cations, such as Bzmim^+^ (Bzmim = 1-benzyl-3-methylimidazolium), fundamentally modify the optoelectronic behavior. When Bzmim^+^ occupies the A-site to form [Bzmim]_3_SbCl_6_, it undergoes phase transitions analogous to those observed in In^3+^-based MHPs [21,87], albeit through distinct pathways. In In^3+^-based MHPs (e.g., Cs_3_InCl_6_), phase transitions involve the replacement of a CsCl unit by H_2_O under humid conditions, accompanied by O^2−^ substituting Cl^−^ in the [InCl_6_]^3−^ octahedral framework [21]. In contrast, the phase transition in [Bzmim]_3_SbCl_6_ is driven by the extraction and reinsertion of [Bzmim]Cl within the [Bzmim]_*n*−3_SbCl*_n_* structure (Fig. 7A) [117]. This structural reorganization induces tunable luminescence: The emission difference between [Bzmim]_3_SbCl_6_ (*λ*_em_ = 525 nm) and [Bzmim]_2_SbCl_5_ (*λ*_em_ = 600 nm) arises from a coordination-induced redshift in the halide–metal charge transfer within the [SbCl*_n_*]^3−*n*^ complexes [118]. This structural reorganization-induced color change can be applied to information encryption. For example, the word “disagree” written on paper was encrypted and required decryption using specific [Bzmim]Cl solution concentrations, thermal treatment, and UV observation (Fig. 7B) [117]. In addition, leveraging the low melting point and thermally responsive luminescence of [Bzmim]_3_SbCl₆, direct laser writing on paper coated with this material enabled reversible information writing and erasure through localized melting and luminescence modulation (Fig. 7C) [118]. A_3_SbX_6_ MHPs also show promising application in scintillation. Abovementioned [Bzmim]_3_SbCl_6_ generates a single radiative center at 540 nm with an L.Y. of 24,600 ph/MeV (Fig. 7D). When integrated into [Bzmim]_3_SbCl_6_@PMMA film scintillator fabricated through this strategy enables noninvasive internal imaging of earphones, demonstrating a high spatial resolution of 8.3 lp/mm (Fig. 7E and F) [119]. In addition to the reported 0D A_3_SbX_6_ MHPs, [SbI_6_]^3−^ isolated octahedra tend to form higher-dimensional architectures (3D and 2D) when constructing OIHPs, while also finding applications in scintillation detection. X-ray detectors fabricated using 2D FPEA_3_SbI_6_ (FPEA = 4-fluorophenethylammonium) exhibit a LoD of 0.123 μGy/s and a spatial resolution of 1.59 lp/mm for imaging applications [120]. Similarly, 3D [Mor]_2_RbSbI_6_ (Mor = morpholinium)-based detectors achieve exceptional sensitivity (1.09 × 10^4^ μC·Gy^−1^·cm^−2^) and an ultralow detection limit of 3.1 nGy/s [105]. In contrast, the 0D Gua_3_SbCl_6_ (Fig. 7G) demonstrates an L.Y. of 2,300 ± 500 ph/MeV under x-ray excitation (Fig. 7H). Furthermore, Sb^3+^-based OIHPs exhibit responsiveness to higher-energy γ-rays: Gua_3_SbCl_6_ achieves an L.Y. of 1,800 ± 500 ph/MeV under 120-keV ^60^Co γ-ray irradiation (Fig. 7I) [121].

### A_3_BiX_6_-type MHPs

As another P-block cation, Bi^3+^ is also a promising B-site constituent for constructing A_3_BiX_6_ MHPs [122]. The greater atomic mass of Bi^3+^ relative to Sb^3+^ reduces lattice vibrational frequencies, thereby enhancing carrier mobility and improving the suitability of A_3_BiX_6_ for photodetector applications [123,124]. The intrinsic luminescence of Cs_3_BiCl_6_ single crystal at 390 nm arises from the parity-forbidden ^3^T_1u_ → ^1^A_1g_ transition of Bi^3+^, while the dual-band absorption at 220 and 330 nm originates from the ^1^A_1g_ → ^1^T_1u_ and ^1^A_1g_ → ^3^T_1u_ transitions, respectively [125]. Similarly, Cs_3_BiBr_6_ NCs display blue emission at RT with a PLQY of merely 6.03%, which could increase to 22% upon oleic acid passivation [126]. The poor luminescence efficiency of Cs_3_BiX_6_ due to strong exciton–phonon coupling and severe nonradiative recombination has shifted research focus toward its potential for UV dual-band photodetection.

**Fig. 8.**
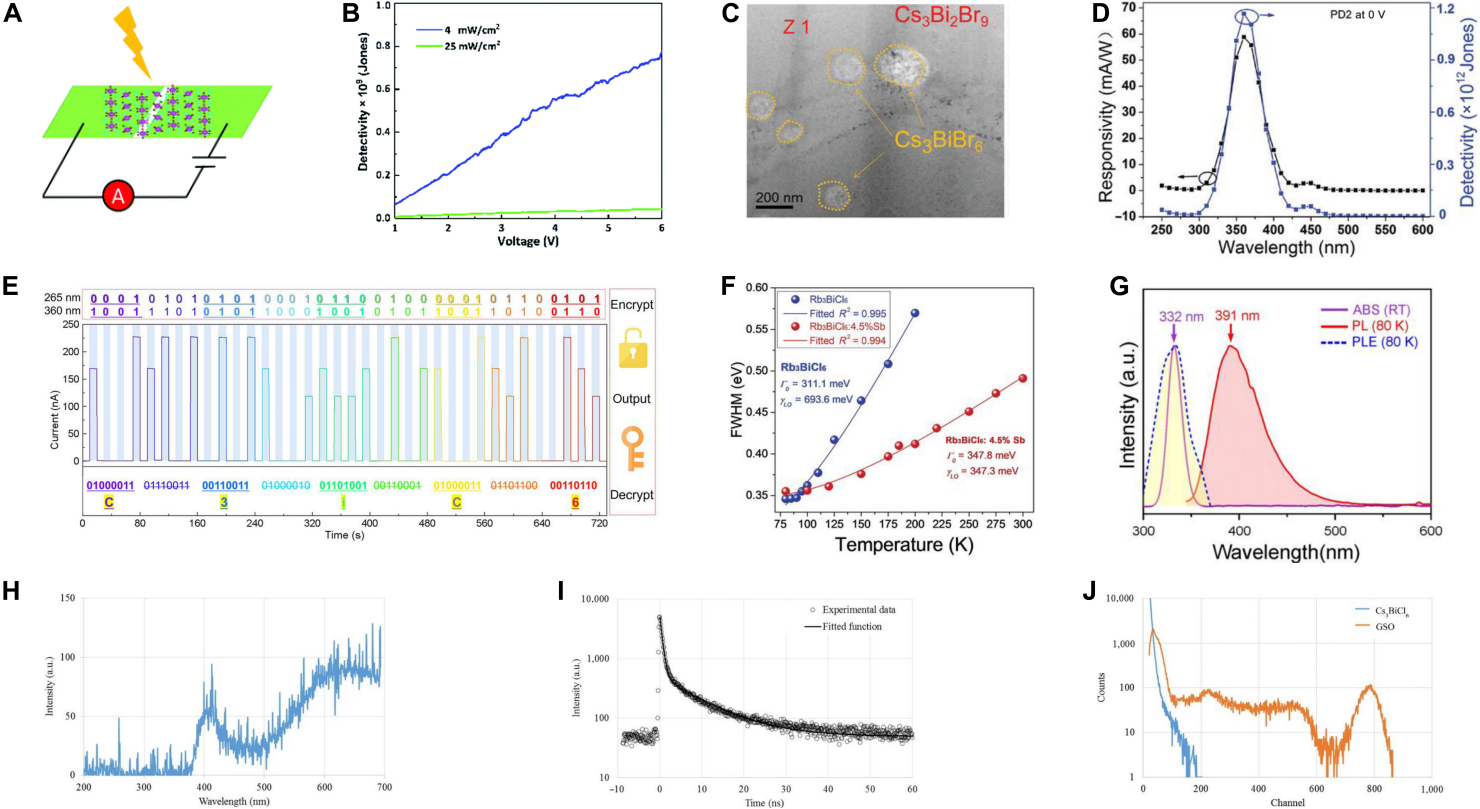
(A) Schematic diagram of the photodetector based on Cs_3_BiBr_6_. Adapted with permission [123]. Copyright 2019, published by Royal Society of Chemistry. (B) Comparison of the detectivity at 2 different light densities. Adapted with permission [123]. Copyright 2019, published by Royal Society of Chemistry. (C) Transmission electron microscopy image for Cs_3_BiBr_6_ and Cs_3_Bi_2_Br_9_ interface. Adapted with permission [127]. Copyright 2022, published by Wiley-VCH. (D) Responsivity and detectivity spectra of Cs_3_BiBr_6_/Cs_3_Bi_2_Br_9_ bulk. Adapted with permission [124]. Copyright 2022, published by Wiley-VCH. (E) Schematic diagram and effective digital data for encrypted photocommunication based on Cs_3_BiCl_6_/GaN heterojunction. Adapted with permission [128]. Copyright 2025, published by Wiley-VCH. (F) Fitting results of temperature-dependent full width at half maximum (FWHM) for Rb_3_BiCl_6_ and Rb_3_BiCl_6_:4.5% Sb^3+^. Adapted with permission [33]. Copyright 2021, published by Wiley-VCH. (G) Absorption, photoluminescence (PL), and PL excitation (PLE) spectra of Cs_3_BiCl_6_ nanocrystals (NCs). Adapted with permission [122]. Copyright 2020, published by Springer. (H) X-ray induced radioluminescence (RL) of Cs_3_BiCl_6_ single crystal. Adapted with permission [125]. Copyright 2016, published by Elsevier. (I) Pulsed x-ray induced decay profile of Cs_3_BiCl_6_ single crystal. Adapted with permission [125]. Copyright 2016, published by Elsevier. (J) Pulse-height spectra of Gd_2_SiO_5_:Ce^3+^ (GSO) and Cs_3_BiCl_6_ under ^137^Cs γ-ray excitation. Adapted with permission [125]. Copyright 2016, published by Elsevier.

The fabricated photodetectors based on Cs_3_BiBr_6_ single crystals show an impressive detectivity of 8 × 10^8^ Jones and a low dark current under 400-nm illumination (Fig. 8A and B) [123]. Given that Cs_3_BiBr_6_ and Cs_3_Bi_2_Br_9_ are interconvertible, Cs_3_BiBr_6_ can undergo phase transformation to form Cs_3_Bi_2_Br_9_ during synthesis using identical precursors [18,122]. Fang’s group [127] successfully constructed a Cs_3_BiBr_6_/Cs_3_Bi_2_Br_9_ bulk heterojunction applying with dual-band photodetection (Fig. 8C). In this regard, the self-powered ITO (ITO = indium tin oxide)/Cs_3_BiBr_6_/Cs_3_Bi_2_Br_9_/Au photodetector achieves responsivities of 59.4 mA/W at 360 nm and 3.09 mA/W at 450 nm (Fig. 8D) [124].

To overcome the limitation of inherent challenges in photodetectors, such as narrow optical absorption range and low carrier mobility, the favorable band alignment between Cs_3_BiCl_6_ and GaN enables the formation of a built-in electric field at their heterojunction interface [128]. The interfacial electric field suppresses carrier recombination and separates photogenerated electron–hole pairs, significantly enhancing responsivity and detection sensitivity. The Cs_3_BiCl_6_/GaN heterojunction photodetector achieves exceptional performance: strong specific detectivity (1.23 × 10^12^ Jones) and ultrafast response (*τ*_r_/*τ*_f_ = 28/190 μs). Its intrinsic UV dual-band selectivity enables applications in logic operations and encrypted optical communication systems (Fig. 8E).

On the one hand, the A_3_BiX_6_ MHPs show strong application into photodetection. On the other hand, the absence of PL in A_3_BiCl_6_ (A = Rb and Cs) at RT is attributed to the strong exciton–phonon coupling with the coupling constant reaching 693 meV, which facilitates severe nonradiative recombination [33], structural and vibrational studies indicate that the RT luminescence quenching of A_3_BiX_6_ is closely related to pronounced local distortion of isolated [BiX_6_]^3−^ octahedra and strong lattice anharmonicity [129]. To enhance the efficiency of radiative recombination, ns^2^-typed Sb^3+^ was incorporated into Rb_3_BiCl_6_ (i.e., Rb_3_BiCl_6_:Sb^3+^), resulting in a bright STE emission with a reduced coupling constant of 347 meV (Fig. 8F), the PLQY increased to 33% [33]. The strategy of structural modifications further improved the PLQY, achieving a maximum of 45% in Rb_2_CsBiCl_6_:Sb^3+^ (Table 7) [130–132]. Beyond ns^2^-type dopants, transition-metal cation Mn^2+^ is also regarded as effective dopant. Due to the primarily participation of Mn d orbitals in the conduction band minimum (CBM), both (Cs_2_K)BiCl_6_:Mn^2+^ and Cs_3_BiCl_6_:Mn^2+^ can exhibit a narrow bandgap and an allowed ^4^T_1_ → ^6^A_1_ transition [18,133]. Direct Mn^2+^ doping yields weak NIR emission, while Sb^3+^, Cr^3+^ codoping enables efficient ET (PLQY = 11.03%). Sb^3+^-induced quasi-2D domains further enhance PLQY to 30.68%, enabling multifunctional NIR LEDs for night vision, penetration imaging, and vein visualization [134].

**Table 7.** The key PL properties of A_3_BiX_6_ (including PL peak, FWHM, Stokes shift, decay time and PLQY)

Compound	Sample	PL peak (nm)	FWHM (nm)	Stokes shift (nm)	Decay time (μs)	PLQY at *λ*_ex_	Ref.
Cs_3_BiCl_6_	NC	391 (80 K)	60	59	–	–	[122]
Cs_3_BiBr_6_	NC	435	–	–	2.77 × 10^−3^ (40.19%)	6.03% (*λ*_ex_ = 372 nm)	[198]
					12.16 × 10^−3^ (26.35%)		
Rb_2_CsBiCl_6_:Sb^3+^	SC	560	133	182	452 × 10^−3^; 1.29	45% (*λ*_ex_ = 378 nm)	[130]
Rb_3_BiCl_6_:Sb^3+^	SC	625	170	245	4.1	33.6% (*λ*_ex_ = 380 nm)	[33]
Cs_3_BiCl_6_:Sb^3+^	SC	555	132	175	0.54; 1.45	15.85% (*λ*_ex_ = 380 nm)	[131]
Cs_3_BiCl_6_:Sb^3+^	SC	885	250		72.08 (765 nm)	30.68% (*λ*_ex_ = 532 nm)	[134]
					329.75 × 10^−3^ (885 nm)		
(Cs_2_K)BiCl_6_:Mn^2+^	SC	605	–	235	60	–	[133]
Cs_3_BiCl_6_:Mn^2+^	NC	607	80	174	20	2.5% (*λ*_ex_ = 430 nm)	[18]

PL, photoluminescence; SC, single crystal; NC, nanocrystal; PLQY, PL quantum yield; FWHM, full width at half maximum.

Although Cs_3_BiCl_6_ exhibits negligible emission at RT, its PL intensity significantly increases when the temperature falls below 80 K, as shown in Fig. 8G [122,125]. This behavior suggests that radiative recombination becomes more favorable at low temperatures under UV excitation, due to the suppression of thermally activated nonradiative processes. Under high-energy radiation excitation, a large number of high-energy electron–hole pairs are generated, creating dense excitation conditions that allow radiative recombination pathways to effectively compete with nonradiative processes. As a result, Cs_3_BiCl_6_ exhibits dual emission bands at 400 and 600 to 700 nm under x-ray excitation, accompanied by fast decay kinetics (*τ*_1_ = 0.61 ns [43%] and *τ*_2_ = 9.4 ns [57%]) (Fig. 8H and I). Notably, an L.Y. of approximately 800 ph/MeV has been achieved under ^137^Cs γ-ray irradiation (Fig. 8J), highlighting the potential of Cs_3_BiCl_6_ for radiation detection applications [125].

### A_3_REX_6_-type MHPs with empty f orbitals

**Fig. 9.**
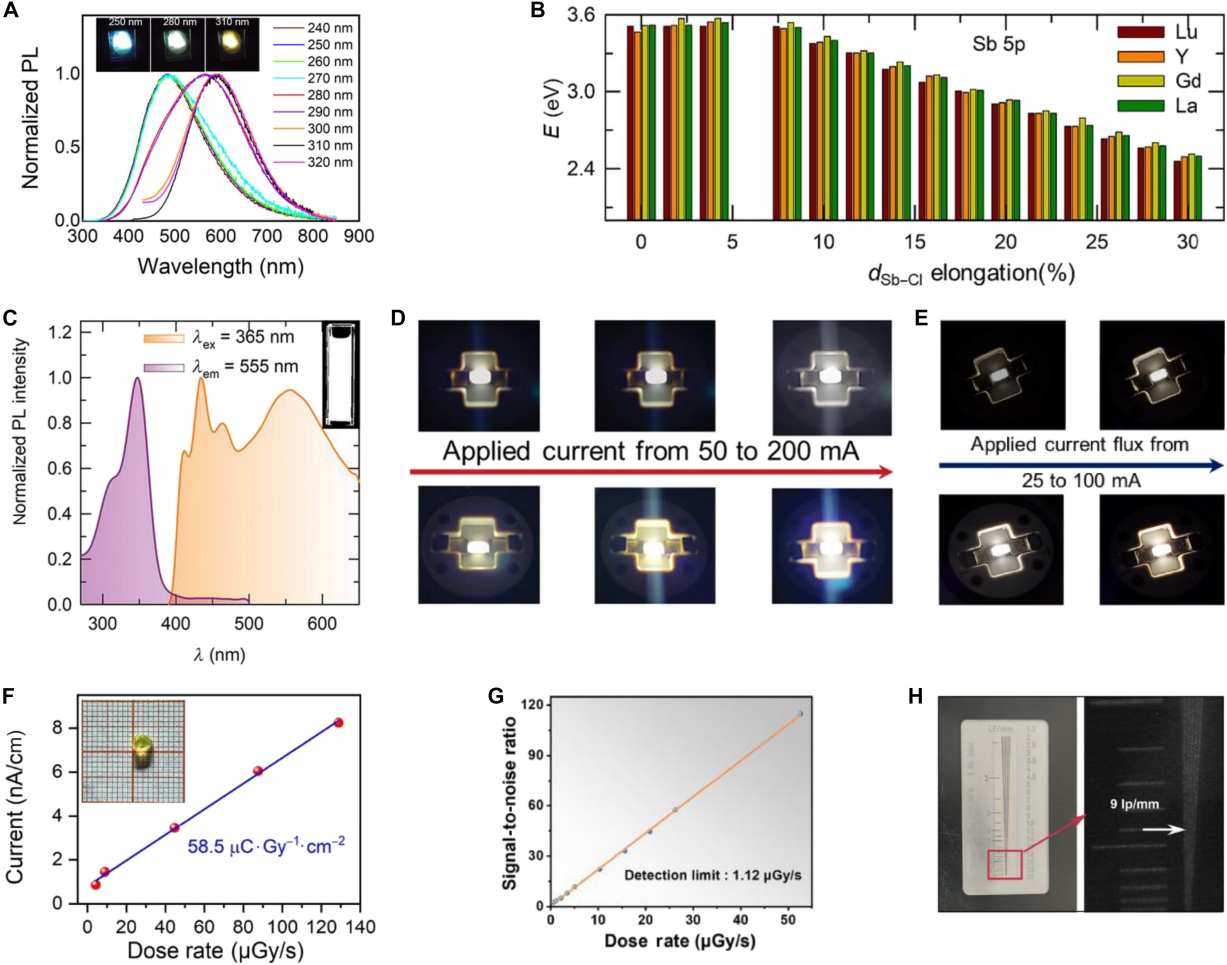
(A) Photoluminescence (PL) spectra of Cs_2_ScCl_5_·H_2_O at room temperature with different excitation wavelengths. Adapted with permission [140]. Copyright 2022, published by Wiley-VCH. (B) The frontier molecular orbital energies of the lowest electronic transitions involving the [SbCl_6_]^3−^ octahedron upon symmetric percentage of elongation of Sb–Cl. Adapted with permission [142]. Copyright 2024, published by Elsevier. (C) Excitation and emission spectra of Cs_3_ScCl_6_:Sb^3+^ nanocrystals (NCs). Adapted with permission [143]. Copyright 2023, published by Wiley-VCH. (D) Photographic images of white light-emitting diode (LED) of Cs_3_ScCl_6_:Sb^3+^ at different applied currents. Adapted with permission [143]. Copyright 2023, published by Wiley-VCH. (E) Photographic images of fabricated white LED device of Cs_3_LaCl_6_:Sb^3+^ NCs at different applied current fluxes. Adapted with permission [142]. Copyright 2024, published by Elsevier. (F) Photocurrent generated by x-ray irradiation at various dose rates under 30-V bias. Adapted with permission [145]. Copyright 2023, published by American Chemical Society. (G) Linear relationship between signal-to-noise ratio value and x-ray dose rate. Adapted with permission [72]. Copyright 2025, published by Springer. (H) The photograph of the standard line-pair card under the visible light (left) and the corresponding x-ray image of the standard line-pair card (right). Adapted with permission [72]. Copyright 2025, published by Springer.

As for the A_3_REX_6_ MHPs, RE^3+^ with empty f orbitals (since La^3+^ has empty f orbitals and Sc^3+^ and Y^3+^ lack f orbitals, these ions are collectively treated as RE^3+^ with empty f orbitals in this work) is similar to the P-block cation as B-site composition, including the intrinsic luminescence mechanism and water-driven phase transition. The intrinsic luminescence for Cs_3_ScCl_6_, Cs_3_YCl_6_, and Cs_3_LaCl_6_ single crystals is ascribed to STE emission [72,135,136], albeit with relatively low efficiencies, as in the case of Cs_3_YCl_6_, the PLQY is merely 3.1% [72]. Relatively, their NCs could achieve better PL properties due to the surface modifications by oleic acid ligands [137,138]. Based on this strategy, the PLQY of Cs_3_YCl_6_ NCs achieves up to 89.3%, while its single crystal is still limited to the modest PLQY (<10%) [139]. The water-driven strategy is also effective for incorporating H_2_O into the lattice, converting A_3_REX_6_ into A_2_REX_5_·H_2_O. The phase-transformed Cs_3_ScX_6_ and Cs_2_ScCl_5_·H_2_O demonstrate excitation wavelength-dependent luminescence at RT (Fig. 9A). The femtosecond transient absorption, temperature-dependent PL, and time-resolved PL studies reveal that this tunable emission originates from 2 distinct STE states. Selective excitation to different STE states through wavelength modulation enables emission color tuning from cyan through white to yellow [140].

Although Cs_2_ScCl_5_·H_2_O exhibits excitation wavelength-dependent PL with low efficiency, using Sb^3+^ as a ns^2^-typed dopant enables direct ET to yellow-emitting STE states, boosting the PLQY to nearly 100% [140]. Similarly, Sb^3+^ doping in Cs_3_ScCl_6_ also enhances the luminescence efficiency. A comparative study of Sb^3+^-doped A_3_REX_6_ MHPs revealed that structural evolution results in tunable emission (Table 8): Cs_3_ScCl_6_:Sb^3+^ emits cyan (500 nm) [141], Cs_3_YCl_6_:Sb^3+^ emits green (545 to 550 nm) [72,142], and Cs_3_LaCl_6_:Sb^3+^ emits yellow (570 nm) [142]. This PL tunability may originate from the local distortion of the [SbCl_6_]^3−^ octahedral in the host lattices (Fig. 9B), which alters the Sb–Cl bond lengths and consequently modulates the energy levels of the frontier molecular orbitals. Intriguingly, Cs_3_ScCl_6_:Sb^3+^ NCs exhibit white emission under 365-nm excitation (Fig. 9C), attributed to the partial transformation into carbon dots during synthesis. The resulting synergy between broad STE emission and blue emission from carbon dots enables full-spectrum coverage, i.e., white light [143]. Similarly, the white LED based on Cs_3_ScCl_6_:Sb^3+^ maintains stable white emission under 200 mA, achieving a high CRI (=90) and a luminous efficacy of 23 lm/W (Fig. 9D) [143]. Likewise, the white LED fabricated from Cs_3_LaCl_6_:Sb^3+^ reaches a CRI of 91 at 25 mA, with brightness further increased at higher currents (Fig. 9E) [142]. Ln^3+^ incorporation enables efficient STE–Ln^3+^ ET for NIR emission; Br^–^ substitution renders the ET efficiency of Cs_3_La_0.5_Nd_0.5_Br_6_ nearly unity and boosts the PLQY of Cs_3_La_0.17_Nd_0.83_Br_6_ NCs to 64.65% (after APTN treatment), enabling NIR LEDs with 3.49% EQE and a 488-min *T*_50_ lifetime [144].

**Table 8.** The key PL properties of A_3_REX_6_ (including PL peak, decay time, and PLQY)

Compound	Sample	PL peak (nm)	Decay time (μs)	PLQY at *λ*_ex_	Ref.
Cs_3_ScCl_6_:Sb^3+^	NC	545	3.6	32% (*λ*_ex_ = 345 nm)	[143]
Cs_3_ScCl_6_:Sb^3+^	NC	555	–	48% (*λ*_ex_ = 365 nm)	[143]
Cs_3_YCl_6_:Sb^3+^	Powder	550	2.37	93.5% (*λ*_ex_ = 345 nm)	[72]
Cs_3_YCl_6_:Sb^3+^	NC	545	3.9	26% (*λ*_ex_ = 320 nm)	[142]
Cs_3_LaCl_6_:Sb^3+^	NC	570	5.4	30% (*λ*_ex_ = 320 nm)	[142]
Cs_3_La_0.17_Nd_0.83_Br_6_	NC	1,070, 1,350	290	64.65% (*λ*_ex_ = 405 nm, NIR)	[144]
Cs_3_LaI_6_:Er^3+^	NC	1,540	6,670	7.8% (*λ*_ex_ = 370 nm)	[187]

PL, photoluminescence; NC, nanocrystal; PLQY, PL quantum yield; NIR, near infrared.

Furthermore, Rb_2_ScCl_5_·H_2_O:Sb^3+^ exhibits x-ray absorption coefficients comparable to CsI and α-Se, with a radiation sensitivity of 58.5 μC·Gy^−1^·cm^−2^ (2.9 times higher than commercial α-Se) (Fig. 9F) [145]. Cs_3_YCl_6_:Sb^3+^ exhibits identical STE emission under x-ray (6.6 mGy/s) as UV excitation, achieving an L.Y. of 16,000 ph/MeV. At an SNR of 3, its detection limit reaches 1.12 μGy/s, far below medical diagnostic thresholds (Fig. 9G). Flexible films of Cs_3_YCl_6_:Sb^3+^ embedded in polydimethylsiloxane (PDMS) enable high-resolution x-ray imaging, resolving internal structures of adapters, earphones, and circuit boards at dose rate of 1.2 mGy/s, with a spatial resolution up to 9 lp/mm (Fig. 9H) [72].

Compared to ns^2^-typed dopants, Ln^3+^ as dopant shows better compatibility with A_3_REX_6_ MHPs for enhancing PL and RL performance. Replacing Y^3+^ with La^3+^ enlarges the [LnCl_6_]^3−^ polyhedra, increasing electron density and inducing greater lattice distortion [142]. This effect is observed in both Ln^3+^ with empty f orbital and Ln^3+^ with f orbital, such as Gd^3+^ and Lu^3+^ [142]. The distortion caused by different Ln^3+^ alters the electronic structure of A_3_REX_6_, directly impacting its luminescent properties [142,143].

**Fig. 10.**
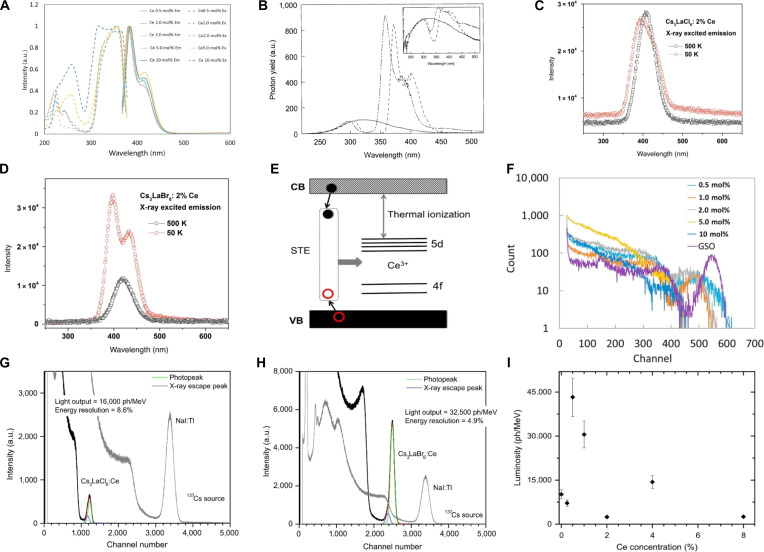
(A) Photoluminescence (PL) and PL excitation (PLE) spectra of Cs_3_ScCl_6_:Ce^3+^ crystals. Em, emission; Ex, excitation. Adapted with permission [135]. Copyright 2020, published by Elsevier. (B) X-ray excited emission spectra of pure Cs_2_LiYCl_6_ (solid line), Cs_2_LiYCl_6_:Ce^3+^ (dashed line), and Li_3_YCl_6_:Ce^3+^ (dashed–dotted line), recorded at room temperature. The inset displays the same spectra plotted with a logarithmic *Y* axis. The emission intensity of the 3 crystals was normalized at 300 nm. Adapted with permission [146]. Copyright 1999, published by Elsevier. (C) Radioluminescence (RL) spectra comparison of Cs_3_LaCl_6_:2% Ce at 50 and 500 K. Adapted with permission [136]. Copyright 2015, published by Elsevier. (D) RL spectra comparison of Cs_3_LaBr_6_:2% Ce at 50 and 500 K. Adapted with permission [136]. Copyright 2015, published by Elsevier. (E) Scheme of Ce^3+^ emission and excitation. Adapted with permission [136]. Copyright 2015, published by Elsevier. (F) Pulse-height spectra of Gd_2_SiO_5_:Ce^3+^ (GSO) and Cs_3_ScCl_6_:Ce^3+^ under ^137^Cs γ-ray excitation. Adapted with permission [135]. Copyright 2020, published by Elsevier. Pulse height spectrum measured on (G) Cs_3_LaCl_6_:Ce^3+^ and (H) Cs_3_LaBr_6_:Ce^3+^ single crystal under ^137^Cs excitation, respectively. A commercial NaI:Tl crystal is used as the reference. Adapted with permission [58]. Copyright 2014, published by Elsevier. (I) Pulsed x-ray measurements of luminosities for Cs_3_LaI_6_ containing different Ce concentrations. Adapted with permission [58]. Copyright 2014, published by Elsevier.

When Ce^3+^ is incorporated as a luminescent center in A_3_REX_6_ host such as Cs_3_ScCl_6_, Li_3_YCl_6_, and Cs_3_LaX_6_ (X = Cl, Br, and I), dual-shoulder characteristics are observed in the emission peaks of PL and RL spectra within low doping levels, accompanied by rapid decay times (Fig. 10A and B) [58,135,146]. In addition, Cs_3_LaCl_6_:Ce^3+^ and Cs_3_LaBr_6_:Ce^3+^ exhibit remarkable thermal quenching resistance, with PL and RL intensities decreasing by less than 25% from 40 to 500 K (Fig. 10C and D). This stability arises from enhanced ET from host-derived STEs to Ce^3+^ ions at elevated temperatures (Fig. 10E) [136]. A similar ET is observed in Cs_3_ScCl_6_:Ce^3+^ [135], underscoring the critical role of STE-to-activator energy migration in thermal stability.

Notably, Ce^3+^-doped A_3_REX_6_ MHPs primarily focus on high-energy scintillation. For instance, Cs_3_ScCl_6_ achieves optimal scintillation properties at 0.5% Ce^3+^ doping, delivering an L.Y. of 9,200 ph/MeV under 662-keV ^137^Cs γ-ray excitation (Fig. 10F) [135]. Similarly, Li_3_YCl_6_ with 1% Ce^3+^ exhibits an L.Y. of 6,185 ph/MeV under identical conditions [146]. High L.Y. and reduced E.R. are observed in Cs_3_LaCl_6_ and Cs_3_LaBr_6_ synthesized via the multiple alternating direction melt-mixing and cooling method at elevated doping concentrations. For example, Cs_3_LaCl_6_:10% Ce^3+^ achieves an L.Y. of 20,000 ph/MeV under 662-keV ^137^Cs γ-ray excitation, with an E.R. of 9% at 40% Ce^3+^. Cs_3_LaBr_6_:10% Ce^3+^ demonstrates even higher performance, reaching 35,000 ph/MeV and 8% E.R. at 40% Ce^3+^ doping [34]. However, crystal quality significantly impacts scintillation metrics. Bridgman-grown Cs_3_LaCl_6_:8% Ce^3+^ and Cs_3_LaBr_6_:15% Ce^3+^ show reduced L.Y. (16,000 and 32,500 ph/MeV, respectively) but improved E.R. (8.6% and 4.9%, respectively) (Fig. 10G and H). Remarkably, Bridgman-synthesized Cs_3_LaI_6_:0.5% Ce^3+^ achieves an exceptional L.Y. of 43,000 ph/MeV (Fig. 10I) [58].

### A_3_LnX_6_ MHPs with f orbitals based on light Ln^3+^

Light lanthanides (Ln^3+^ = La^3+^, Ce^3+^, Pr^3+^, Nd^3+^, Sm^3+^, and Eu^3+^) with f orbitals, when incorporated B-site luminescent center ions of A_3_LnX_6_ MHPs, exhibit fundamentally different luminescence and scintillation properties relative to A_3_REX_6_ MHPs lacking f orbitals. This difference originates from the unfilled 4f*^n^* electron configuration of Ln^3+^ ions, which gives rise to characteristic 4f–4f or 5d–4f transitions. In particular, Eu^3+^ and Ce^3+^ serve as representative Ln^3+^ emitters (Table 9), whose bright luminescence stems from the strong shielding of the 4f orbitals by the outer 5s^2^5p^6^ orbitals.

**Table 9.** The key PL properties of A_3_LnX_6_ (including PL peak, decay time, and PLQY)

Compound	Sample	PL peak (nm)	Decay time (ns)	PLQY at *λ*_ex_	Ref.
Cs_3_CeCl_6_	SC	380, 410	13.61 (2.93%)	99.89% (*λ*_ex_ = 365 nm)	[199]
			40.75 (97.07%)		
Cs_3_CeBr_6_	Powder	392, 421	29.1	80% (*λ*_ex_ = 365 nm)	[185]
Cs_3_CeBr_6_	Powder	390, 421	28.33 (390 nm)	89.94% (*λ*_ex_ = 365 nm)	[15]
			28.67 (421)		
Cs_3_CeBr_6_	Powder	391, 421	28.7 (391 nm)	92.5% (*λ*_ex_ = 350 nm)	[73]
			27.1 (425 nm)		
Cs_3_CeBr_6_	SC	395, 425	8.06 (3.06%)	92.57% (*λ*_ex_ = 365 nm)	[199]
			43.53 (96.94%)		
Cs_3_CeI_6_	SC	427, 470	45.44	7.53% (*λ*_ex_ = 365 nm)	[199]
Cs_3_CeI_6_	Powder	430, 470	26.1	76.2% (*λ*_ex_ = 365 nm)	[13]
Rb_3_CeI_6_	Film	427, 468	22.3 (472 nm)	51% (*λ*_ex_ = 280 nm)	[186]
			25 (468 nm)		
Cs_3_CeCl_6_:Gd^3+^	NC	376, 408	42.8	96% (*λ*_ex_ = 365 nm)	[155]
Cs_3_EuCl_6_	Powder	592, 614	4.35 × 10^6^ (*λ*_ex_ = 334 nm)	92.4% (*λ*_ex_ = 365 nm)	[158]
			4.11 × 10^6^ (*λ*_ex_ = 465 nm)		
Cs_3_EuCl_6_	NC	590, 611, 652, 700	3.99 × 10^6^	48.78% (*λ*_ex_ = 280 nm)	[147]
Cs_3_EuCl_6_	NC	430	25.79	5.38% (*λ*_ex_ = 350 nm)	[147]
EHC–Cs_3_EuCl_6_	NC	617	3.09 × 10^6^	92.4% (*λ*_ex_ = 365 nm)	[150]

PL, photoluminescence; SC, single crystal; NC, nanocrystal; PLQY, PL quantum yield.

**Fig. 11.**
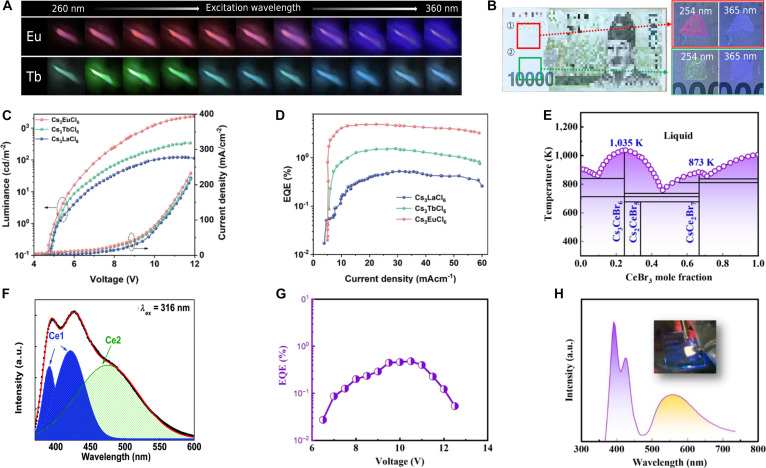
(A) Photoluminescence (PL) Photographs of Cs_3_EuCl_6_ and Cs_3_TbCl_6_ nanocrystal (NC) films under excitation wavelengths from 260 to 360 nm with a 10-nm interval. Adapted with permission [147]. Copyright 2023, published by Royal Society of Chemistry. (B) Photographs of anticounterfeiting marks formed by transfer printing under ambient, 254-nm, and 365-nm ultraviolet (UV) lights. Adapted with permission [147]. Copyright 2023, published by Royal Society of Chemistry. (C) Current density–voltage and luminance–voltage curves. Adapted with permission [150]. Copyright 2024, published by Wiley-VCH. (D) external quantum efficiency (EQE)–current density curves of Cs_3_LaCl_6_, Cs_3_EuCl_6_, and Cs_3_TbCl_6_ light-emitting diode (LEDs). Adapted with permission [150]. Copyright 2024, published by Wiley-VCH. (E) The phase diagrams of CeBr_3_–CsBr binary system. Adapted with permission [73]. Copyright 2021, published by Royal Society of Chemistry. (F) PL spectra of CsCe_2_Br_7_ single crystals at 38 K. Adapted with permission [151]. Copyright 2015, published by American Chemical Society. (G) The EQE–voltage curve of Cs_3_CeBr_6_ LED. Adapted with permission [73]. Copyright 2021, published by American Chemical Society. (H) Spectrum of the white LED under forwarding voltage. The inset plot shows the photograph of the white LED device. Adapted with permission [73]. Copyright 2021, published by American Chemical Society.

As Eu^3+^ is incorporated as B-site host composition, it tends to undergo partial reduction to Eu^2+^, resulting in the coexistence of both dual oxidation states within distinct luminescence characteristics. Specifically, Eu^3+^ contributes red emission via f–f transitions, while Eu^2+^ emits blue light through the 4f^7^ → 4f^6^5d^1^ transition. Exploiting this duality, Cs_3_EuCl_6_ NCs exhibit excitation-wavelength-dependent multicolor emission, ranging from red to deep blue [147]. A similar photochromic effect is also observed in Cs_3_TbCl_6_ (Fig. 11A); both Cs_3_EuCl_6_ and Cs_3_TbCl_6_ NCs have been utilized in anticounterfeiting inks, enabling dynamic and multicolor pattern encoding (Fig. 11B) [147]. To further enhance PL properties of Cs_3_EuCl_6_, constructing ET channels using organic ligands represents another effective strategy [148,149]. For instance, the small molecule ethyl 7-hydroxycoumarin-3-carboxylate (EHC) coordinates with undercoordinated Ln^3+^ through its carbonyl group, passivating surface defects. Furthermore, its singlet excited state exhibits strong spectral overlap with Ln^3+^’s absorption, significantly boosting the overall ET efficiency [150]. Based on this strategy, EHC-passivated Cs_3_EuCl_6_ achieves PLQY of 92.4%. The LED devices based on EHC-Cs_3_EuCl_6_ achieved an EQE of 5.17% and an *L*_max_ of 370 cd/m^2^ (Fig. 11C and D). Notably, the red LED based on EHC-Cs_3_EuCl_6_ NCs achieved a *T*_50_ of 440 h at an initial luminance of 100 cd/m^2^ [150], outperforming most reported MHP-based LEDs. However, the *T*_50_ of the EHC-Cs_3_EuCl_6_ LED remains inferior to commercial LEDs due to defect accumulation, halide migration, interfacial degradation, and thermally activated instability under continuous operation.

**Fig. 12.**
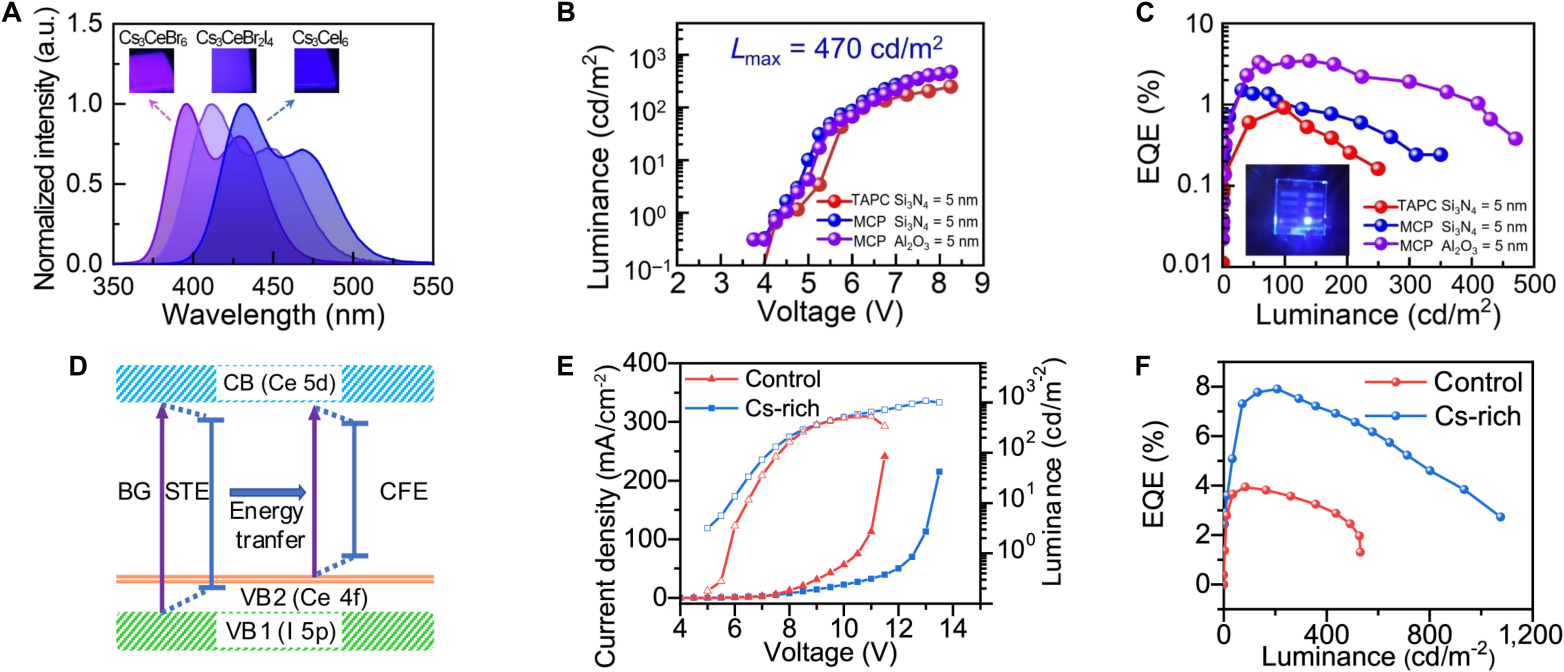
(A) Photoluminescence (PL) spectra of Cs_3_CeBr_6_, Cs_3_CeBr_2_I_4_, and Cs_3_CeI_6_, with the inset showing the corresponding photographs under ultraviolet (UV) light radiation. Adapted with permission [13]. Copyright 2022, published by American Association for the Advancement of Science. (B) Voltage–luminance curves of Cs_3_CeI_6_ light-emitting diode (LEDs). Adapted with permission [13]. Copyright 2022, published by American Association for the Advancement of Science. (C) External quantum efficiency (EQE)–luminance curves of Cs_3_CeI_6_ LEDs. Adapted with permission [13]. Copyright 2022, published by American Association for the Advancement of Science. (D) Schematic diagram of Cs_3_CeI_6_ energy band structure and 2 types of excitons. Adapted with permission [14]. Copyright 2024, published by Springer Nature. (E) EQE–luminance curve of Cs_3_CeI_6_ control and Cs-rich devices. Adapted with permission [14]. Copyright 2024, published by Springer Nature. (F) Current density–voltage–luminance curve of Cs_3_CeI_6_ control and Cs-rich devices. Adapted with permission [14]. Copyright 2024, published by Springer Nature.

The emission of A_3_CeX_6_ also originates from the parity-allowed 5d–4f transition, resulting in a fully permitted emission in the UV or blue spectral regions with nanosecond-scaled lifetime [73]. According to the CsBr–CeBr_3_ phase diagram in Fig. 11E, the stable phases are limited to Cs_3_CeBr_6_ and CsCe_2_Br_7_ [73]. As for the PL application, Cs_3_CeBr_6_ featuring a single Ce^3+^ emissive center, good stability, and few defects compared with CsCe_2_Br_7_ with the dual Ce^3+^ emissive centers (Fig. 11F) [151] exhibits a high PLQY of ~90% and a fast decay time of ~28 ns. Tang et al. fabricated an LED device with Cs_3_CeBr_6_ as the active layer via a dual-source coevaporation method, achieving an *L*_max_ of 25 cd/m^2^ and an EQE of 0.46% (Fig. 11G), and obtained a white LED by combining it with a yellow Y_3_Al_5_O_12_:Ce phosphor down-converter (Fig. 11H) [73]. Compared to Cs_3_CeBr_6_, Cs_3_CeI_6_ enables spectral tuning by shifting the emission from the UV to deep-blue region (Fig. 12A), which can result in a narrower spectral emission and enhanced color purity in LEDs [13,14]. Density functional theory (DFT) calculations further reveal that Cs_3_CeI_6_ possesses a higher exciton binding energy (225 meV) than Cs_3_CeBr_6_ (135 meV) [73,152]. Furthermore, the film fabrication process was optimized; the high-quality, dense, and uniform Cs_3_CeI_6_ thin films were fabricated using a 5-nm CsCl seed layer and dual-source coevaporation; and the resulting deep-blue LED device (ITO/ZnO/Al_2_O_3_/Cs_3_CeI_6_/MCP/TAPC/HAT-CN/Al) achieved an *L*_max_ of 470 cd/m^2^ and an EQE_max_ of 3.5% (Fig. 12B and C) [13]. The luminescence mechanism of Cs_3_CeI_6_ is driven by ET from STEs of I_2_^−^ species to Ce^3+^-based Frenkel excitons (CFEs) (Fig. 12D), and increasing STE-CFE spectral overlap with excess CsI could improve ET process, resulting in a deep-blue LED with high *L*_max_ of 1,075 cd/m^2^ and an EQE_max_ of 7.9% (Fig. 12E and F) [14].

**Fig. 13.**
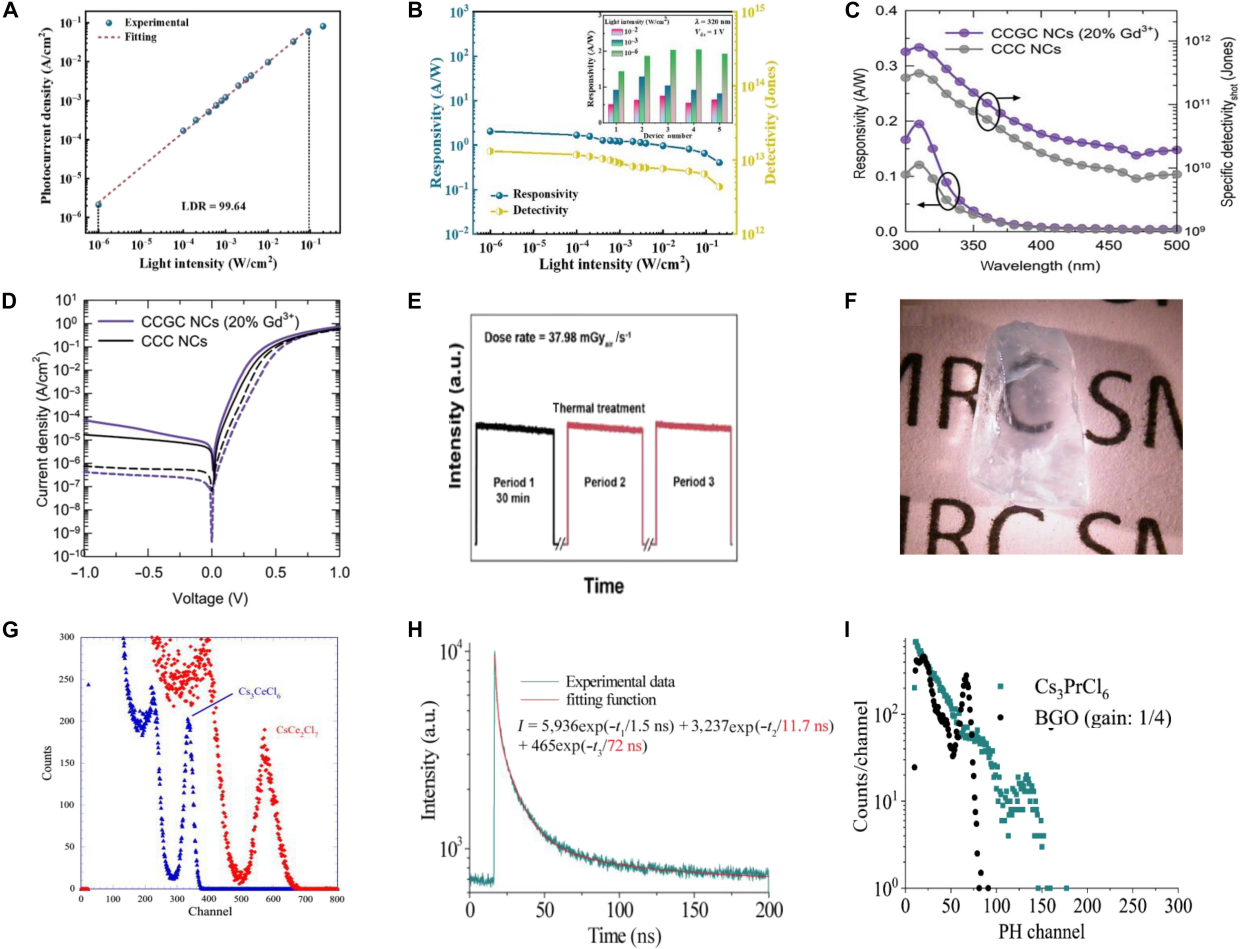
(A) The logarithmic curve of light-intensity-dependent photocurrent density and corresponding power law fit curve under 320-nm light illumination. LDR, linear dynamic range. Adapted with permission [15]. Copyright 2024, published by Elsevier. (B) Responsivity and detectivity of the optimum photodetector versus the light intensity. Adapted with permission [15]. Copyright 2024, published by Elsevier. (C) Spectral responsivity and specific detectivity of 0% and 20% Gd^3+^-alloyed CCGC photodetector. Adapted with permission [155]. Copyright 2024, published by Wiley-VCH. (D) *J*–*V* curves of 0% and 20% Gd^3+^-alloyed CCGC photodetector in illumination (310 nm) in solid line and dark conditions in dashed line. Adapted with permission [155]. Copyright 2024, published by Wiley-VCH. (E) Radioluminescence intensity of the (MTP)_3_EuCl_6_ glass after high-dose irradiation, followed by heating at 150 °C for 1 h to restore scintillation performance. Adapted with permission [159]. Copyright 2025, published by Wiley-VCH. (F) Image of a polished slice of Cs_3_CeCl_6_ showing good transparency needed for efficient light emission. Adapted with permission [160]. Copyright 2013, published by Institute of Electrical and Electronics Engineers. (G) Pulse-height spectra of Cs_3_CeCl_6_ single crystals under excitation from 662-keV photons from a ^137^Cs source. Adapted with permission [160]. Copyright 2013, published by Institute of Electrical and Electronics Engineers. (H) Pulsed x-ray-excited scintillation decay time profile of Cs_3_PrCl_6_. Adapted with permission [163]. Copyright 2022, published by M Y U Scientific Publishing Division. (I) ^137^Cs γ-ray induced scintillation pulse height spectra of the Cs_3_PrCl_6_ and a reference Bi_4_Ge_3_O_12_ (BGO) crystal. Adapted with permission [163]. PH, pulse height. Copyright 2022, published by M Y U Scientific Publishing Division.

The narrowband absorption of Cs_3_CeBr_6_ enables solar-blind UV detection without requiring additional optical filters, making it highly suitable for UV photodetector applications [15]. In general, Cs_3_CeBr_6_ films prepared by spin-coating, suffer from high trap-state densities and relatively low carrier mobility, limiting their utility in UV photodetectors. Seok et al. through a postannealing optimization process reduced the trap state density in Cs_3_CeBr_6_ films to approximately 8 × 10^12^ cm^−2^·eV^−1^ while simultaneously increasing the field-effect carrier mobility to about 10 cm^2^·V^−1^·s^−1^. The UV photodetectors fabricated from the optimized films demonstrated a high responsivity of 2.05 A/W, a detectivity of 1.48 × 10^13^ Jones (Fig. 13A), and a wide linear dynamic range of 99.64 dB (Fig. 13B). The optimized Cs_3_CeBr_6_ films as the UV photodetector also exhibited fast response times (rise time, 93 ms; fall time, 345 ms), excellent spectral selectivity, and remarkable stability, retaining ~91% of their initial photocurrent even after 60 d of storage without encapsulation [15]. Similar to Cs_3_CeBr_6_, Cs_3_CeCl_6_ features effectively UV absorption and convert it into electrical signals, making its potential for UV photodetectors. While Cl^−^ substitution of Br^−^ improves intrinsic thermal stability, the strong polarity of the Ce–Cl bond renders the material prone to oxidation and hydrolysis reactions [153,154]. To address this, Gd^3+^ is introduced into the crystal lattice of Cs_3_CeCl_6_. On the one hand, the incorporation of Gd^3+^ enhances material stability and improves film compactness, thereby boosting charge transport efficiency. On the other hand, the partial substitution of Ce^3+^ sites by Gd^3+^ increases the interionic distance between Ce^3+^, suppressing concentration quenching effects, reducing radiative reabsorption, and elevating the PLQY to 96%, which enhances detector sensitivity. Ultimately, the UV photodetector fabricated based on Gd-doped Cs_3_CeCl_6_:Gd^3+^ thin films demonstrates high detectivity (7.938 × 10^11^ Jones), high responsivity (0.195 A/W) and the dark current densities at 0- and −0.1-V bias voltages are as low as 4.4 × 10^−10^ and 1.9 × 10^−7^ A/cm^2^, respectively (Fig. 13C and D) [155].

Owing to the excellent x-ray response of Ce^3+^ and Eu^3+^, A_3_CeX_6_ and A_3_EuX_6_ have been explored for x-ray imaging applications [156]. The Cs_3_CeCl_6_ film achieved a spatial resolution of 11 lp/mm [157], while the Cs_3_EuCl_6_@PDMS composite film realized a spatial resolution of 9 lp/mm [158]. In addition, (MTP)_3_EuCl_6_ (MTP = methyl(triphenyl)-phosphonium) glass exhibited a spatial resolution of 14.3 lp/mm together with recoverable radiation stability (Fig. 13E) [159].

Beyond its applications in photodetectors, electroluminescent LEDs, and x-ray imaging, the utility of A_3_CeX_6_ extends to γ-ray scintillation. Under ^137^Cs γ-ray irradiation, Cs_3_CeCl_6_ demonstrates an L.Y. of 19,000 ph/MeV and an E.R. of 8.4% [17]. Slow cooling to reduce light-scattering defect concentrations enabled the growth of a large single crystal (Φ10 mm × 30 mm, Fig. 13F), thereby enhancing the L.Y. to 21,000 ph/MeV and lowering the E.R. to 7.6% (Fig. 13G) [160]. However, challenges such as internal stress, insufficient release of crystallization latent heat, and by-product formation persist in the Bridgman method. In contrast, mechanical grinding emerges as an eco-friendly alternative, enhancing luminescent performance and thermal stability while minimizing by-products and chemical waste. Mechanically ground Cs_3_CeCl_6_, Cs_3_TbCl_6_, and Cs_3_EuCl_6_ powders exhibit x-ray-induced quantum tunneling effects, where high-energy ionizing radiation facilitates electron tunneling between trap states and the conduction band [161]. The hydrated phase Cs_3_CeCl_6_·3H_2_O was also synthesized, which preserves the excellent scintillation performance of Cs_3_CeCl_6_ while benefiting enhanced properties due to water incorporation. Under x-ray irradiation, it achieves a high L.Y. of 31,900 ph/MeV, surpassing commercial LuAG:Ce. It exhibits a LoD as low as 108 nGy/s and a spatial resolution of 12 lp/mm, making it suitable for high-resolution imaging applications [162]. As similar as nanosecond-scaled decay time with Cs_3_CeCl_6_, Cs_3_PrCl_6_ exhibits the 4f^1^5d^1^ → 4f^2^ transition with fast scintillating decay time (11.7 ns [53%] and 72 ns [47%]) (Fig. 13H) and L.Y. of 5,900 ph/MeV under ^137^Cs γ-ray excitation (Fig. 13I) [163]. As expected, Cs_3_CeCl_6_ and Cs_3_PrCl_6_ are promising scintillators as radiation detectors in time-of-flight positron emission tomography.

### A_3_LnX_6_ MHPs with f orbitals based on heavy Ln^3+^

**Fig. 14.**
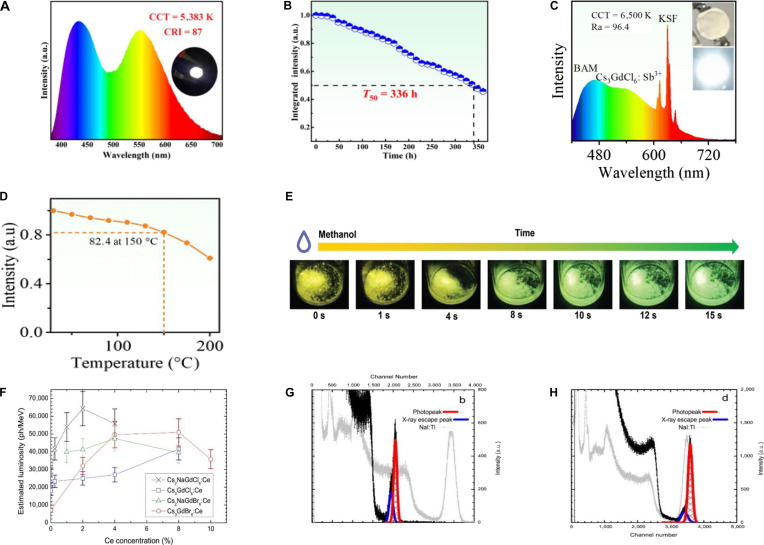
(A) Photoluminescence (PL) spectrum of the fabricated white light-emitting diode (LED) prepared by Cs_3_TmCl_6_. Adapted with permission [27]. Copyright 2024, published by American Chemical Society. (B) Long-term running test showing the *T*_50_ of the white LED. Adapted with permission [27]. Copyright 2024, published by American Chemical Society. (C) Emission spectrum of the white LED device fabricated by blending the yellowish-emitting Cs_3_GdCl_6_:1.0% Sb^3+^ powder with the commercial BaMgAl_10_O_17_:Eu^2+^ and K_2_SiF_6_:Mn^4+^ on a 365-nm chip. Adapted with permission [22]. Copyright 2024, published by Royal Society of Chemistry. (D) Integrated PL intensities of the Cs_3_GdCl_6_:Sb^3+^ MCs as a function of temperature. Adapted with permission [22]. Copyright 2024, published by Royal Society of Chemistry. (E) The in situ dynamic emission color transformation of Cs_2_YbCl_5_·H_2_O:Sb^3+^ to Cs_3_YbCl_6_:Sb^3+^ by methanol. Adapted with permission [165]. Copyright 2024, published by Wiley-VCH. (F) Estimated luminosity of polycrystalline samples (Cs_2_NaGdCl_6_:Ce^3+^, Cs_3_GdCl_6_:Ce^3+^, Cs_2_NaGdBr_6_:Ce^3+^, and Cs_3_GdBr_6_:Ce^3+^) under x-ray irradiation versus Ce concentration. Adapted with permission [32]. Copyright 2014, published by Elsevier. (G and H) γ-Ray pulse height spectra of Cs_3_GdCl_6_:Ce^3+^ and Cs_3_GdBr_6_:Ce^3+^, respectively. Adapted with permission [32]. Copyright 2014, published by Elsevier.

Although some medium-heavy Ln^3+^ possess electrons in their f orbitals, A_3_LnX_6_ MHPs still exhibit both intrinsic luminescence and characteristic f–f or d–f transition. In general, Gd^3+^, Tm^3+^, and Yb^3+^ are utilized as B-site host composition, the PLQY of Cs_3_GdCl_6_ NCs, Cs_3_TmCl_6_ NCs, and Cs_3_YbCl_6_ NCs after oleic acid passivation is 30.3%, 22.8%, and 59.8%, respectively, attributed to intrinsic STE emission [27,139]. Herein, the white LED based on Cs_3_TmCl_6_ shows a CRI of 87.0, a CCT of 5,283 K (Fig. 14A), and a *T*_50_ of 336 h (Fig. 14B), surpassing the *T*_50_ reported in prior studies [27].

In A_3_LnX_6_, the incorporation of ns^2^-typed Sb^3+^ dopant induces local Jahn–Teller distortions owing to the low-symmetry coordination environment, thereby enhancing STE emission [164]. The extent of distortion depends on the specific B-site host composition. For example, Cs_3_GdCl_6_:Sb^3+^, Cs_3_YbCl_6_:Sb^3+^, and Cs_3_LuCl_6_:Sb^3+^ emits at ~545 nm [22,142], 533 nm [165], and 525 nm [166], respectively, and their PLQY could increase to 96.3%, 66.9%, and 99% (Table 10) [22,165,166]. The white LED based on Cs_3_GdCl_6_:Sb^3+^ exhibits a high CRI of 96.5 (Fig. 14C). Furthermore, excellent thermal quenching resistance enable the long-term operation of white LED (Fig. 14D) [22]. Meanwhile, the white LED based on Cs_3_LuCl_6_:Sb^3+^ exhibits excellent color quality, with a high CRI of 94 and a CCT of 5,565 K, and also shows strong resistance to thermal quenching [166]. Interestingly, Cs_3_YbCl_6_:Sb^3+^ allowed a reversible transformation to Cs_2_YbCl_5_·H_2_O:Sb^3+^, which is analogous to transformation from Cs_3_InCl_6_:Sb^3+^ to Cs_2_InCl_5_·H_2_O:Sb^3+^ [87,165]. The water-driven strategy would be triggered rapidly after exposure to methanol (within 15 s), accompanying with an emission color change from yellow to green (Fig. 14E) [165]. This stimulus-responsive luminescence switching reveals the potential of information encryption as the former turns green, while the latter remains yellow [165]. Moreover, Cs_3_LuCl_6_:Sb^3+^ delivers 7,300 ph/MeV and a LoD of 0.4 μGy/s, with flexible films achieving a spatial resolution of 11.5 lp/mm [167]. A Cs_2_NaLuCl_6_:Sb^3+^/Cs_3_LuCl_6_:Sb^3+^ heterojunction enhances RL to 2.4× BGO and lowers the detection limit to 140 nGy/s and a spatial resolution of 14 lp/mm, while Mn^2+^ incorporation enables trichromatic emission for color x-ray imaging [69]

**Table 10.** The key PL properties of A_3_LnX_6_ (including PL peak, FWHM, Stokes shift, decay time and PLQY)

Compound	Sample	PL peak (nm)	FWHM (nm)	Stokes shift (nm)	Decay time	PLQY at *λ*_ex_	Ref.
Cs_3_TbCl_6_	Powder	547	–	–	6.8 ms	90.8% (*λ*_ex_ = 280 nm)	[83]
Cs_3_DyI_6_	NC	488, 574, 660, 1,540	92	118	–	–	[187]
Cs_3_TmCl_6_	NC	440	88	70	1.5 ns (76.6%)	22.8% (*λ*_ex_ = 370 nm)	[27]
					7.1 ns (23.4%)		
Cs_3_YbCl_6_	NC	429	–	–	1–2 ns (93%)	89.3% (*λ*_ex_ = 355 nm)	[139]
					8–9 ns (7%)		
Cs_3_GdCl_6_:Sb^3+^	NC	550	–	–	4.1 μs	28% (*λ*_ex_ = 320 nm)	[142]
Cs_3_GdCl_6_:Sb^3+^	Powder	540	120	–	2.96 μs	96.3% (*λ*_ex_ = 330 nm)	[22]
Cs_3_TbCl_6_:Sb^3+^	NC	450, 489, 548, 621, 700	8.6	225	7.6 ms	48.18% (*λ*_ex_ = 280 nm)	[16]
Cs_3_Tb_0.1_Ce_0.9_Cl_6_	NC	470, 405, 546	–	–	33.04 ns (405 nm)	86% (*λ*_ex_ = 290 nm)	[171]
Cs_3_DyI_6_:Er^3+^	NC	488, 574, 660, 1,540	–	–	9.7 ns	87.4% (*λ*_ex_ = 370 nm)	[187]
Cs_3_YbCl_6_:Sb^3+^	SC	533	120	206	1.66 μs	66.9% (*λ*_ex_ = 365 nm)	[165]
Cs_3_LuCl_6_:Sb^3+^	NC	525	–	–	2.8 μs	13% (*λ*_ex_ = 320 nm)	[142]
Cs_3_LuCl_6_:Sb^3+^	NC	531	104	191	1.96 μs	92% (*λ*_ex_ = 340 nm)	[167]
Cs_3_LuCl_6_:Sb^3+^	Powder	380, 420	–	30, 70	33.76 ns (380 nm)	82.02% (*λ*_ex_ = 350 nm)	[188]
					34.11 ns (420 nm)		
Cs_3_LuCl_6_:Ce^3+^	Powder	382	57	42	26.6 ns	6% (*λ*_ex_ = 335 nm)	[189]
Cs_3_LuCl_6_:Tb^3+^	Powder	549	12	275	–	62% (*λ*_ex_ = 335 nm)	[189]
Cs_3_LuCl_6_:Ce^3+^, Sm^3+^	Powder	379	26	39	18.7 ns	5% (*λ*_ex_ = 335 nm)	[189]

PL, photoluminescence; SC, single crystal; NC, nanocrystal; PLQY, PL quantum yield; FWHM, full width at half maximum.

**Fig. 15.**
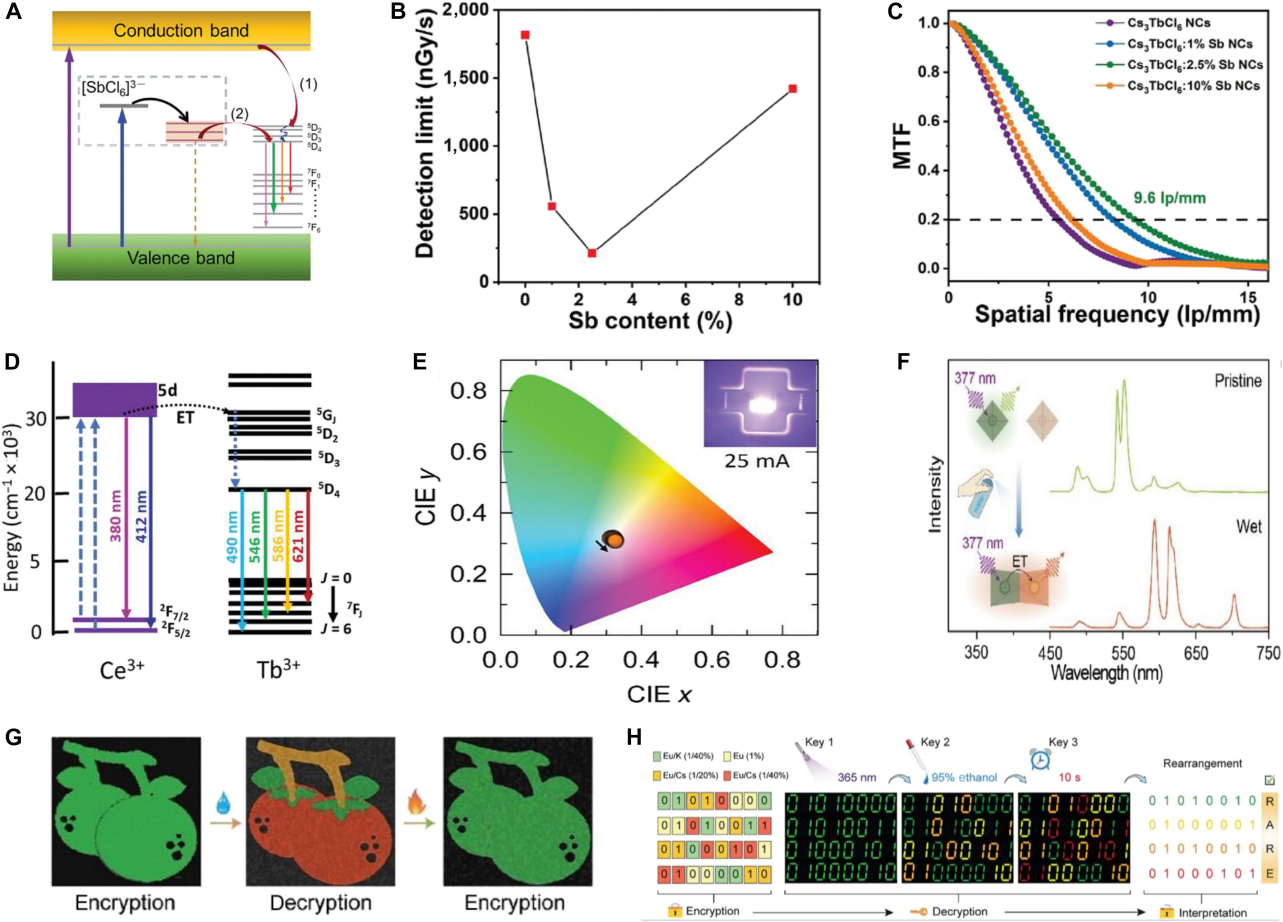
(A) The charge–carrier dynamics model and photophysical processes of Cs_3_TbCl_6_:Sb^3+^ nanocrystals (NCs). (1) Transfer process assisted by Cl^−^-Tb^3+^ charge transition in [TbCl_6_]^3−^; (2) thermally boosting energy transfer (ET) assisted by [SbCl_6_]^3−^-induced self-trapped exciton (STE) state. Adapted with permission [16]. Copyright 2024, published by Wiley-VCH. (B) The variation of detection limit of Cs_3_TbCl_6_:Sb^3+^ NCs as a function of Sb/Tb ratio. Adapted with permission [16]. Copyright 2024, published by Wiley-VCH. (C) Modulation transfer function (MTF) of x-ray images from undoped and Sb^3+^-doped Cs_3_TbCl_6_ NCs. Adapted with permission [16]. Copyright 2024, published by Wiley-VCH. (D) Ce^3+^ to Tb^3+^ ET process in Cs_3_Tb_x_Ce_1-x_Cl_6_ alloy NCs. Adapted with permission [171]. Copyright 2024, published by Wiley-VCH. (E) Commission Internationale de l’Éclairage (CIE) coordinate of white light-emitting diode (LED) based on Cs_3_Tb_0.5_Ce_0.5_Cl_6_. Adapted with permission [142]. Copyright 2024, published by Wiley-VCH. (F) Emission spectra of Cs_3_TbF_6_:Eu^3+^ crystals before and after water treatment. Adapted with permission [169]. Copyright 2022, published by American Chemical Society. (G) Luminescence photographs of the orange pattern under 365-nm ultraviolet (UV) lamp excitation in a wetting–drying cycle. Adapted with permission [169]. Copyright 2022, published by American Chemical Society. (H) The encryption of ACSII binary codes and decryption process. Adapted with permission [168]. Copyright 2024, published by Wiley-VCH.

Beyond the intrinsic luminescence, Ln^3+^ as B-site host and dopant enables the tunable emission with ET process, based on the characteristic f–f or d–f transitions (Fig. 15A) [19,168,169]. Under x-ray excitation, 0.5-in Cs_3_GdCl_6_:Ce^3+^ and Cs_3_GdBr_6_:Ce^3+^ exhibit the L.Y. of 40,000 and 48,000 ph/MeV, respectively (Fig. 14F). When excited by ^137^Cs γ-ray, their L.Y. reaches 24,500 and 47,000 ph/MeV, with E.R. of 4.5% and 4.0%, respectively (Fig. 14G and H). Relatively, Cs_3_GdBr_6_:Ce^3+^ delivers the highest L.Y. and lowest E.R. reported among A_3_BX_6_ MHPs under ^137^Cs γ-ray excitation [32]. Cs_3_TbCl_6_:Sb^3+^ NCs achieved an L.Y. of 20,300 ph/MeV, a reduced detection limit of 212 nGy/s (Fig. 15B), and an improved spatial resolution of 9.6 lp/mm (Fig. 15C). They further enabled high-contrast imaging of circuit features across a wide range of irradiation doses [16].

Although ET process toward Ln^3+^ to Ln^3+^ is environmentally insensitive, it mainly involves quadrupole–quadrupole [170], Cs_3_Tb_0.5_Ce_0.5_Cl_6_ establishes an efficient ET pathway of Ce^3+^ → Tb^3+^ channel (Fig. 15D), suppressing nonradiative recombination and achieving a PLQY of up to 86%. The white LED based on Cs_3_Tb_0.5_Ce_0.5_Cl_6_ exhibited a CRI of ~78 and a light efficiency of ~118 lm/W at 25 mA (Fig. 15E) [171]. In addition to Ce^3+^-sensitized dopant, A_3_TbF_6_:Eu^3+^ (A = Cs and Rb) demonstrates stimulus-responsive luminescence based on humidity-induced phase transitions. Initially, weak orbital coupling between Eu^3+^ and Tb^3+^ results in low ET efficiency, with dominant green emission from Tb^3+^. Upon exposure to a humid environment, the material undergoes a phase transition to A_2_TbF_5_:Eu^3+^, where shortening interionic distance and triggering efficient energy coupling between Tb^3+^ and Eu^3+^ activate the ET process and characteristic Eu^3+^ red emission (Fig. 15F) [168,169]. Screen-printed patterns utilizing differently doped Cs_3_TbF_6_ crystals have been developed for information encryption, where concealed multicolor graphics become visible only under specific stimulus conditions (Fig. 15G) [169]. Furthermore, beyond their kinetically tunable hydrochromic luminescence properties, Rb_3_TbF_6_:Eu^3+^ crystals also exhibit excitation-wavelength-dependent luminescence phenomena, attributed to the unique light absorption of Eu^3+^ at 393 nm. Leveraging this phenomenon, 4 distinct sample sets can generate varying arrays of color combinations depending on the excitation/stimulus conditions. These combinations can serve as identification codes for encryption (Fig. 15H) [168].

**Fig. 16.**
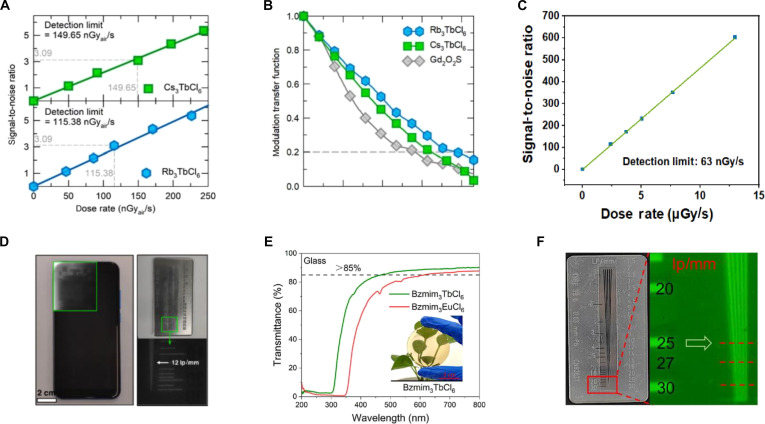
(A) The linear response between radioluminescence (RL) intensity and x-ray dose rate of Cs_3_TbCl_6_ and Rb_3_TbCl_6_ under low-dose-rate excitation. Their detection limits can be defined, as the signal-to-noise ratio (SNR) is 3. Adapted with permission [31]. Copyright 2022, published by American Chemical Society. (B) Spatial resolution measurement of Cs_3_TbCl_6_, Rb_3_TbCl_6_, and Gd_2_O_2_S scintillator films. Adapted with permission [31]. Copyright 2022, published by American Chemical Society. (C) The linear relationship between SNR value of Cs_3_TbCl_6_ and x-ray dose rate. Adapted with permission [83]. Copyright 2025, published by Springer. (D) The photograph of a smartphone (left) the standard line-pair card under the visible light (top) and the corresponding x-ray image of the standard line-pair card (bottom). Adapted with permission [83]. Copyright 2025, published by Springer. (E) Transmission spectra of Bzmim_3_LnCl_6_ glass. Adapted with permission [172]. Copyright 2025, published by Wiley-VCH. (F) Spatial resolution of the Bzmim_3_TbCl_6_ glass scintillation demonstrated using a line pair card. Adapted with permission [172]. Copyright 2025, published by Wiley-VCH.

As one of the high-efficiency scintillator, A_3_TbCl_6_ (A = Cs and Rb) exhibits similar excited-state relaxation pathways under both low- and high-energy excitations [161]. The scintillation properties of A_3_TbCl_6_ are dependent on its structural form (e.g., polycrystalline, microcrystalline, or glass), which, in turn, is dictated by the synthesis route. The performance divergence between polycrystal and microcrystal forms is largely dictated by variations in grain size, orientation, grain boundary density, and film compactness. Benefiting from large grain sizes, Cs_3_TbCl_6_ and Rb_3_TbCl_6_ polycrystals achieve impressive L.Y. of 56,800 and 88,800 ph/MeV, respectively, with LoD of 149.65 and 115.38 nGy/s (Fig. 16A) under x-ray excitation. The spatial resolution of Cs_3_TbCl_6_ and Rb_3_TbCl_6_ polycrystals films reach 3.3 and 3.9 lp/mm, respectively (Fig. 16B) [31]. Although the reduced grain size of RT recrystallized Cs_3_TbCl_6_ microcrystals led to a lower L.Y. of 51,800 ph/MeV, this refinement in microstructure enabled the fabrication of a denser, more uniform film. This resulted in a significantly improved spatial resolution of 12 lp/mm and a lower LoD of 63 nGy/s (Fig. 16C and D) [83]. In addition, the glass scintillator prepared by substituting Cs^+^ with organic groups (e.g., Bzmim^+^), although resulting in a decrease in L.Y., the high transmittance of the glass has achieved a high spatial resolution of 25 lp/mm (Fig. 16E and F) [172]. In general, polycrystals typically achieve high L.Y.s owing to their large grain size; microcrystals facilitate the fabrication of dense films, leading to a low detection limit; and highly transparent glasses offer high spatial resolution. Introducing emissive fluorophores to construct ET channels with Ln^3+^ enhances radiative performance, as exemplified by (BuTPP)_3_TbCl_6_ (BuTPP = butyltriphenylphosphonium) glass achieving 8,100 ph/MeV of L.Y., 0.93 μGy/s of LoD, and 26.8 lp/mm of spatial resolution [173].

## Machine Learning for A_3_BX_6_ MHPs with Enhanced Radiative Transitions

**Fig. 17.**
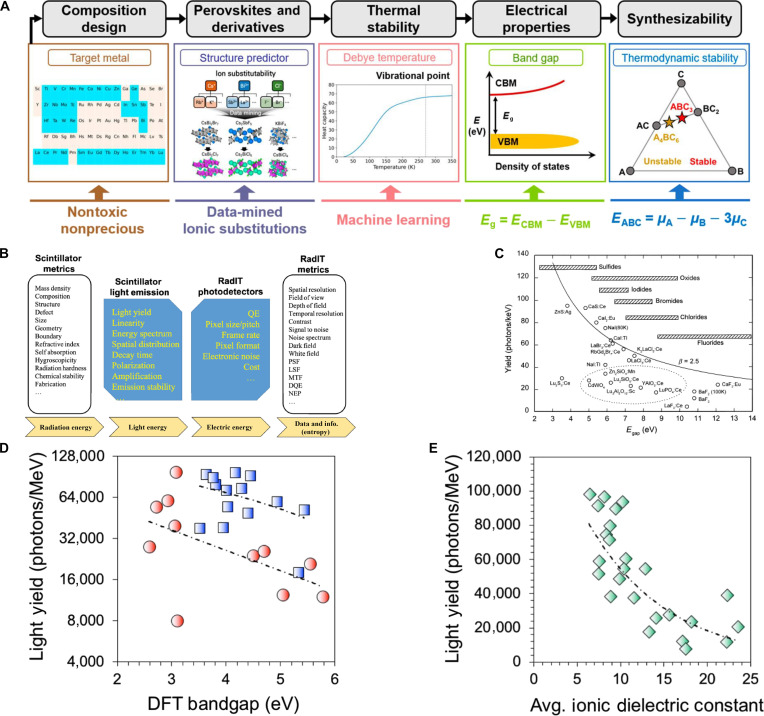
(A) Workflow of computational exploration for inorganic halide perovskite derivatives. Adapted with permission [174]. Copyright 2022, published by American Chemical Society. (B) Radiographic imaging and tomography (RadIT) metrics and scintillator metrics are correlated through the energy and data (information) flows. Adapted with permission [175]. PSF, point spread function; LSF, line spread function; DQE, detective quantum efficiency; NEP, noise equivalent power. Copyright 2023, published by Institute of Electrical and Electronics Engineers Inc. (C) Empirical trend between light yield and material’s bandgap for a range of scintillator compounds. Adapted with permission [184]. Copyright 2002, published by Elsevier. (D) Trends of experimentally reported light yield (L.Y.) with density functional theory (DFT)-computed bandgaps and (E) averaged ionic part of the dielectric constants. Adapted with permission [176]. Copyright 2019, published by Springer.

Exploring the broad compositional landscape of A_3_BX_6_ perovskites remains challenging using conventional approaches, this is mainly reflected in long experimental cycles, high material costs, and limited reproducibility, motivating the introduction of machine learning (ML) as an efficient tool for high-throughput prediction and guided synthesis. Im et al. [174] demonstrated this potential by constructing a 1,703-composition Cs-B-X library and using crystal graph convolutional neural networks to learn structural features, ultimately identifying 40 thermodynamically stable compounds (Fig. 17A). Among them, 27 have been experimentally verified, while several other experimentally confirmed that stable compounds were not captured by the ML predictions.

Such structural stability mapping is particularly relevant for scintillators, as the accessible compositional phase space constrains the formation of thermodynamically stable hosts and helps preselect candidates with favorable electronic and optical properties, such as bandgap width. Building on this foundation, ML can act as a “virtual experimentalist” by learning quantitative correlations between host structures and photophysical properties, enabling the rapid evaluation of thousands of candidate materials prior to synthesis (Fig. 17B) [175].

For scintillators, the fundamental L.Y. can be approximated as the product of 3 constituent processes: absorption, ET, and luminescence Eq. (13) [176]:

$$L.Y.=\frac{E_{\gamma }}{\beta E_{g}}\cdot \eta_{2}\cdot \eta_{3}$$ (13)

where *E*_g_ is the bandgap of scintillators, *E*_γ_ is the energy of the incident radiation, and *η*_2_ and *η*_3_ are the efficiencies of ET and intracenter luminescence, respectively. Herein, the *E*_g_ can be efficiently computed from electronic structure calculations, serving as a physically meaningful descriptor for ML-based prediction of L.Y. (Fig. 17C).

Beyond *E*_g_, the overall L.Y. also depends on the carrier recombination efficiency, which is governed by the interplay between the thermalization length and the Onsager radius (*R*_Ons_). *R*_Ons_ represents the distance at which the Coulomb potential between an electron and hole equals the thermal energy and can be expressed as Eq. (14) [177]:

$$R_{\mathrm{Ons}}=\frac{e^{2}}{4{\pi \varepsilon }_{0}\varepsilon_{r}k_{B}T}$$ (14)

where *e* is the elementary charge, *k*_B_ is the Boltzmann constant, *ε*_0_ is the permittivity of free space, *T* is the absolute temperature, and *ε*_r_ is the relative dielectric constant of the host (including both ionic and electronic contributions). Thermalized carriers separated by a distance *r* recombine with probability 1 − exp(−*R*_Ons_/*r*) or escape with probability exp(−*R*_Ons_/*r*). Therefore, *R*_Ons_ and *ε*_r_ directly influence electron–hole recombination and serve as key descriptors for ML prediction of L.Y. at RT.

Together, Pilania et al. [176] investigated a set of 25 Eu^2+^- and Ce^3+^-doped scintillators by computing bandgaps, densities, and both ionic and electronic contributions to the dielectric constant using DFT. ML models based on Kernel Ridge Regression and AdaBoost were then trained to predict L.Y. and scintillation performance. Their results show that both a smaller *E*_g_ and a lower ionic contribution to *ε*_r_ tend to achieve higher L.Y. (Fig. 17D and E) [176].

Although ML methods have shown great potential in predicting the properties of perovskite materials, their practical application still faces several limitations. On the one hand, compared with datasets in bioinformatics or genomic prediction that often contain tens of thousands of entries, the datasets currently available for perovskite structure and property prediction remain relatively small, and the descriptors used to characterize luminescence performance are still limited. On the other hand, light emission is fundamentally governed by excited-state carrier dynamics and electron–phonon coupling, which cannot be fully captured by ground-state DFT descriptors. Consequently, predicting luminescence properties solely based on ground-state DFT parameters has intrinsic limitations. Notably, recent studies have shown that methods such as time-dependent DFT, GW–Bethe–Salpeter equation method, and surface hopping can more effectively describe the excited-state properties of materials [178–180].

## Conclusion and Prospects

A_3_BX_6_ MHPs feature tunable electronic structures, highly tolerant crystal lattices, and functional charge carriers generated under optical or electrical excitation, which endow them with broad application prospects in optoelectronic and scintillation applications. This review summarizes recent progress in A_3_BX_6_ MHPs (A_3_SbX_6_, A_3_InX_6_, A_3_BiX_6_, A_3_REX_6_, and A_3_LnX_6_) with a focus on B-site dopant engineering and its influence on radiative transition mechanisms. B-site cation engineering effectively modulates the electronic structures and luminescence pathways in these materials. Specifically, ns^2^-typed dopant induce STE emission, while transition-metal and RE dopants activate characteristic d–d, d–f, and f–f transitions, enabling tunable PL and EL. In addition to conventional light emission applications, A_3_BX_6_ MHPs also exhibit excellent performance in x-ray and γ-ray detection, owing to their strong high-energy photon absorption, low self-absorption, and broad spectral response.

Despite their tunable luminescence and enhanced scintillation performance, the practical development of A_3_BX_6_ MHPs continues to face substantial challenges and opportunities, including but not limited to the following:

1. The high CRI of A_3_BX_6_ MHPs is advantageous for white-light LED applications; however, the inherently broad emission spectra limit the achievable color purity for narrowband or monochromatic LEDs. In addition, the relatively low PLQY in the NIR spectral region may constrain their performance in bioimaging, NIR sensing, and other NIR photonic applications.
2. Although the EQE of deep-blue A_3_BX_6_ LEDs reaches 7.9%, which is relatively high for this challenging spectral region, the EQE of green and red LEDs remains below the reported records, indicating that further optimization of charge carrier injection, recombination efficiency, and material stability is required.
3. In the field of x-ray scintillators, RE A_3_BX_6_ MHPs are promising candidates for high-resolution x-ray imaging due to their high L.Y. (maximum L.Y. = 88,800 ph/MeV), low detection limit (minimum LoD = 63 nGy/s), and high spatial resolution (highest spatial frequency = 18.5 lp/mm). However, current studies predominantly report NCs or submillimeter-sized single crystals, which critically influences edge emission, photon escape, suboptimal light coupling, and reduced optical transmittance, thereby limiting the overall scintillation performance. To address this, further advances in crystal growth techniques and scalable strategies for producing centimeter-scale single crystals are essential to obtain the superior scintillation properties.
4. In the field of γ-ray scintillators, RE A_3_BX_6_ MHPs have demonstrated considerable promise owing to their high L.Y. (maximum L.Y. = 47,000 ph/MeV) and low E.R. (minimum E.R. = 4.0%). However, these values remain inferior to those of state-of-the-art scintillators such as LaBr_3_:Ce, which exhibits an L.Y. of 74,196 ph/MeV and an E.R. of ~3% at 662 keV [181]. In general, the high scintillation performance of A_3_BX_6_ MHPs has been mainly achieved by Ce^3+^ doping. However, the characteristic energy levels of Ce^3+^ limit further optimization of L.Y. and decay-time, hindering time-critical applications such as time-of-flight position emission tomography. Future studies should explore multicomponent doping or codoping strategies to require fast response and high photon output.
5. Compared to CsPbX_3_ perovskites, A_3_BX_6_ MHPs are intrinsically lead-free and exhibit good thermal and radiation stability due to their vacancy-ordered framework and strong B–X bonding. However, environmental factors may still affect their long-term performance. Under high-humidity conditions, certain compositions may undergo hydration-induced phase transitions, which introduce structural defects and degrade luminescence performance. To enhance moisture resistance, strategies such as encapsulation, surface passivation, halide substitution, and lattice rigidification via cation engineering can be used to suppress ion migration and structural rearrangement. Under prolonged irradiation, A_3_BX_6_ MHPs generally demonstrate high radiation hardness. For example, Rb_3_InCl_6_:Sb^3+^ exhibits nearly unchanged RL intensity under a cumulative dose of 561.6 Gy, while Cs_2_NaLuCl_6_/Cs_3_LuCl_6_:Sb^3+^ maintains stable RL output up to 1,000 Gy. Nevertheless, sustained irradiation may still induce defect accumulation and halide vacancy formation, thereby increasing nonradiative recombination pathways. Further improvement in radiation tolerance requires minimizing initial defect densities, enhancing crystal quality, and strengthening structural stability through cation engineering.

Overall, the above strategies demonstrate that B-site cation engineering is central to achieving enhanced luminescence and scintillation in A_3_BX_6_ MHPs, providing a foundation for further optimization of synthesis methods, dopant–host compatibility, and interface modification. The incorporation of ns^2^, transition-metal, and RE dopants enables diverse radiative pathways, supporting applications ranging from PL and EL to high-energy radiation detection. Despite the substantial progress achieved, challenges remain in large-scale single-crystal growth, long-term stability, and standardized device evaluation. Future advances may be accelerated by artificial-intelligence-assisted materials discovery, improved crystal engineering, and deeper understanding of carrier dynamics, paving the way for the practical implementation of A_3_BX_6_ MHPs in next-generation optoelectronic and radiation detection technologies.

## References

[1] Ou X, Chen X, Xu X, Xie L, Chen X, Hong Z, Bai H, Liu X, Chen Q, Li L, et al. Recent development in x-ray imaging technology: Future and challenges. *Research*. 2021;**2021**:Article 9892152. 10.34133/2021/9892152 PMC8724686 35028585

[2] Lian H, Zhang W, Zou R, Gu S, Kuang R, Zhu Y, Zhang X, Ma C-G, Wang J, Li Y. Aqueous-based inorganic colloidal halide perovskites customizing liquid scintillators. Adv Mater. 2023;35(51):2304743. 10.1002/adma.202304743 37722107

[3] Zhou Y, Chen J, Bakr OM, Mohammed OF. Metal halide perovskites for x-ray imaging scintillators and detectors. ACS Energy Lett. 2021;6(2):739–768.

[4] Feng SC, Hu XM, Shen Y, Li YQ, Tang JX, Lee ST. Phase regulation of layered perovskites toward high-performance light-emitting diodes. Adv Funct Mater. 2025;35(21):2310220.

[5] Xu Q, Wang J, Shao W, Ouyang X, Wang X, Zhang X, Guo Y, Ouyang X. A solution-processed zero-dimensional all-inorganic perovskite scintillator for high resolution gamma-ray spectroscopy detection. Nanoscale. 2020;12(17):9727–9732. 32323687 10.1039/d0nr00772b

[6] Tourougui S, Alla M, El-assib H, Rouchdi M, Fares B. Next-generation lead-free halide perovskites: CsInX_3_ (X = Br, cl) for high-efficiency solar cells. Computat Conden Matter. 2026;46: Article e01193.

[7] Borges-Martínez M, Saavedra-Torres M, Schott E, Zarate X. Computational design and properties elucidation of new (FAPbI_3_)_1−x-y_(MAPbBr_3_)_y_(CsPbBr_3_)_x_ photoactive systems for their application in perovskite solar cells. Mater Today Commun. 2023;34: Article 105324.

[8] Chen Q, Wu J, Ou X, Huang B, Almutlaq J, Zhumekenov AA, Guan X, Han S, Liang L, Yi Z. All-inorganic perovskite nanocrystal scintillators. Nature. 2018;561(7721):88–93. 30150772 10.1038/s41586-018-0451-1

[9] He Y, Matei L, Jung HJ, McCall KM, Chen M, Stoumpos CC, Liu Z, Peters JA, Chung DY, Wessels BW. High spectral resolution of gamma-rays at room temperature by perovskite CsPbBr_3_ single crystals. Nat Commun. 2018;9(1):1609. 29686385 10.1038/s41467-018-04073-3 PMC5913317

[10] Pu G, Wang R, Tang Y, Song J, Wang J. Cation doping engineering of metal halide perovskite for high-energy x-ray exploration. Mater Chem Front. 2025;9(13):1945–1197.

[11] Xiao Z, Song Z, Yan Y. From lead halide perovskites to lead-free metal halide perovskites and perovskite derivatives. Adv Mater. 2019;31(47):1803792. 10.1002/adma.201803792 30680809

[12] Buñuel MA, Lozano L, Chaminade JP, Moine B, Jacquier B. Optical properties of Tb^3+^-doped Rb_2_KInF_6_ elpasolite. Opt Mater. 1999;13(2):211–223.

[13] Guo Q, Wang L, Yang L, Duan J, Du H, Ji G, Liu N, Zhao X, Chen C, Xu L. Spectra stable deep-blue light-emitting diodes based on cryolite-like cerium(III) halides with nanosecond df emission. Sci Adv. 2022;8(50): Article eabq2148. 36525491 10.1126/sciadv.abq2148 PMC9757739

[14] Yang L, Du H, Li J, Luo Y, Lin X, Pang J, Liu Y, Gao L, He S, Kang J-W. Efficient deep-blue electroluminescence from Ce-based metal halide. Nat Commun. 2024;15(1):6240. 39048553 10.1038/s41467-024-50508-5 PMC11269580

[15] Panchanan S, Dastgeer G, Dutta S, Hu M, Lee S-U, Im J, Seok SI. Cerium-based halide perovskite derivatives: A promising alternative for lead-free narrowband UV photodetection. Matter. 2024;7(11):3949–3969.

[16] Zhou W, Yu Y, Han P, Li C, Wu T, Ding Z, Liu R, Zhang R, Luo C, Li H. Sb-doped Cs_3_TbCl_6_ nanocrystals for highly efficient narrow-band green emission and x-ray imaging. Adv Mater. 2024;36(2):2302140. 10.1002/adma.202302140 37801733

[17] Zhuravleva M, Yang K, Melcher C. Crystal growth and scintillation properties of Cs_3_CeCl_6_ and CsCe_2_Cl_7_. J Cryst Growth. 2011;318(1):809–812.

[18] Yan J, Zhang S, Wei Q, Cao S, Zhao J, Zou B, Zeng R. Stoichiometry-controlled phase engineering of cesium bismuth halides and reversible structure switch. Adv Opt Mater. 2022;10(5):2101406.

[19] Han JH, Samanta T, Cho HB, Jang SW, Viswanath N, Kim YR, Seo JM, Im WB. Intense hydrochromic photon upconversion from lead-free 0D metal halides for water detection and information encryption. Adv Mater. 2023;35(40):2302442. 10.1002/adma.202302442 37399104

[20] Yang M, Ge W, Teranishi T. Unveiling the antithermal quenching behavior in 0D inorganic metal halide Cs_2_InCl_5_ (H_2_O) mediated by upconversion emission. Inorg Chem. 2024;63(51):24400–24409. 39663568 10.1021/acs.inorgchem.4c04557

[21] Gong Z, Zheng W, Huang P, Cheng X, Zhang W, Zhang M, Han S, Chen X. Highly efficient Sb^3+^ emitters in 0D cesium indium chloride nanocrystals with switchable photoluminescence through water-triggered structural transformation. Nano Today. 2022;44: Article 101460.

[22] Liang X, Zhang W, Shi Y, Zhang W, Yang H, Huang P, Li L, Zhang Q, Zheng W, Chen X. Sb^3+^–doped 0D Cs_3_GdCl_6_ microcrystals with a near-unity photoluminescence quantum yield and high thermal quenching resistance for light-emitting application. J Mater Chem C. 2024;12(15):5538–5548.

[23] Li Q, Xu B, Chen Z, Han J, Tan L, Luo Z, Shen P, Quan Z. Excitation-dependent emission color tuning of 0D Cs_2_InBr_5_·H_2_O at high pressure. Adv Funct Mater. 2021;31(38):2104923.

[24] Li J, Sheng Y, Tong G, Zhu H, Tao X, Wu C, Chang Y, Tang Z, Yang J, Zhang S. Anti-solvent synthesis of three-color indium-based halide perovskite microplate/microcrystal phosphors for high color rendering WLEDs. Adv Opt Mater. 2023;11(16):2300100.

[25] Wang Y, Wen X, Shi H, Mihóková E, Kucerkova R, Babin V, Zhu J, Xiao J, Nikl M, OuYang X, et al. Giant scintillation yield enhancement in zero-dimensional halides by exciton confinement manipulation. *Research*. 2026;**9**:Article 1230. 10.34133/research.1230 PMC13077130 41988020

[26] Song J, Li J, Li X, Xu L, Dong Y, Zeng H. Quantum dot light-emitting diodes based on inorganic perovskite cesium lead halides (CsPbX_3_). Adv Mater. 2015;27(44):7162–7167. 26444873 10.1002/adma.201502567

[27] Wang M, Chen X, Zhang F, Ma Z, Ji X, Cheng S, Pan G, Wu D, Li X-J, Zhang Y, et al. Colloidal synthesis of blue-emitting Cs_3_TmCl_6_ nanocrystals via localized Excitonic recombination for down-conversion white light-emitting diodes. ACS Nano. 2024;18(44):30421–30432. 39455431 10.1021/acsnano.4c07641

[28] Zhang B, Wu X, Zhou S, Liang G, Hu Q. Self-trapped exciton emission in inorganic copper(I) metal halides. Front Optoelectron. 2021;14(4):459–472. 36637760 10.1007/s12200-021-1133-4 PMC9743870

[29] Wang T, Zeng G, Yang YM, Yang Z, Wang T, Li H, Han L, Yu X, Xu X, Ouyang X. Advances in metal halide perovskite scintillators for x-ray detection. Nano-Micro Lett. 2025;17(1):275. 10.1007/s40820-025-01772-7 PMC12102060 40407959

[30] Ma W, Su Y, Zhang Q, Deng C, Pasquali L, Zhu W, Tian Y, Ran P, Chen Z, Yang G. Thermally activated delayed fluorescence (TADF) organic molecules for efficient x-ray scintillation and imaging. Nat Mater. 2022;21(2):210–216. 34764429 10.1038/s41563-021-01132-x

[31] Han JH, Samanta T, Park YM, Kim HJ, Manikanta Viswanath NS, Kim HW, Cha BK, Cho SB, Im WB. Highly stable zero-dimensional lead-free metal halides for x-ray imaging. ACS Energy Lett. 2022;8(1):545–552.

[32] Samulon E, Gundiah G, Gascón M, Khodyuk I, Derenzo S, Bizarri G, Bourret-Courchesne E. Luminescence and scintillation properties of Ce^3+^-activated Cs_2_NaGdCl_6_, Cs_3_GdCl_6_, Cs_2_NaGdBr_6_ and Cs_3_GdBr_6_. J Lumin. 2014;153:64–72.

[33] Zhou L, Liao JF, Qin Y, Wang XD, Wei JH, Li M, Kuang DB, He R. Activation of self-trapped emission in stable bismuth-halide perovskite by suppressing strong exciton–phonon coupling. Adv Funct Mater. 2021;31(31):2102654.

[34] Wei H, Zhuravleva M, Tyagi M, Melcher CL. Scintillation properties of Cs_3_LaCl_6_:Ce^3+^ and Cs_3_LaBr_6_:Ce^3+^. IEEE Trans Nucl Sci. 2013;61(1):390–396.

[35] Liu M, Duan C-K, Tanner PA, Ma C-G, Yin M. Rationalizing the photoluminescence of Bi^3+^ and Sb^3+^ in double perovskite halide crystals. J Phys Chem C. 2021;125(48):26670–26678.

[36] Nie J, Zhou B, Fang S, Wang Y, Wang Y, Tian B, Hu H, Zhong H, Li H, Shi Y. Chemical doping of lead-free metal-halide-perovskite related materials for efficient white-light photoluminescence. Mater Today Phys. 2023;31: Article 100992.

[37] Ming J, Chen Y, Miao H, Fan Y, Wang S, Chen Z, Guo Z, Guo Z, Qi L, Wang X, et al. High-brightness transition metal-sensitized lanthanide near-infrared luminescent nanoparticles. Nat Photonics. 2024;18(12):1254–1262.

[38] Ming J, Xie Z, Wu J, Zhang F. Synthesis of transition metal-sensitized lanthanide near-infrared luminescent nanoparticles. Nat Protoc. 2025;21(4):1544–1573. 40908364 10.1038/s41596-025-01245-6

[39] Kong Q, Yang B, Chen J, Zhang R, Liu S, Zheng D, Zhang H, Liu Q, Wang Y, Han K. Phase engineering of cesium manganese bromides nanocrystals with color-tunable emission. Angew Chem Int Ed Engl. 2021;60(36):19653–19659. 34151496 10.1002/anie.202105413

[40] Morad V, Cherniukh I, Pöttschacher L, Shynkarenko Y, Yakunin S, Kovalenko MV. Manganese(II) in tetrahedral halide environment: Factors governing bright green luminescence. Chem Mater. 2019;31(24):10161–10169. 32952294 10.1021/acs.chemmater.9b03782 PMC7493303

[41] Feng X, Zhang L, Feng X, You J, Pi J, Zeng H, Chu D, Xue C, Zhao K, Jia S. Ion migration suppression via doping multivalent cations in perovskite for high thermal stability x-ray detectors. ACS Energy Lett. 2025;10(2):685–695.

[42] Yin H, Song B, Tong Y, Zhan Y, Wei Q, Cai P, Liu Z, Li J, Chen J, Liu J. Ultrafast scintillator based on zirconium-doped cesium zinc chloride single crystals and their charge carrier dynamics. Laser Photonics Rev. 2024;18(12):2400394.

[43] Lu M, Zhang X, Bai X, Wu H, Shen X, Zhang Y, Zhang W, Zheng W, Song H, Yu WW. Spontaneous silver doping and surface passivation of CsPbI_3_ perovskite active layer enable light-emitting devices with an external quantum efficiency of 11.2%. ACS Energy Lett. 2018;3(7):1571–1577. 30505950 10.1021/acsenergylett.8b00835 PMC6269143

[44] Ji F, Huang Y, Wang F, Kobera L, Xie F, Klarbring J, Abbrent S, Brus J, Yin C, Simak SI. Near-infrared light-responsive Cu-doped Cs_2_AgBiBr_6_. Adv Funct Mater. 2020;30(51):2005521.

[45] Jiang C, Liu Q, Li L, Li K, Feng Y, Fu Y, Li Y, Qian X, Wei B, Du P. Tailoring of visible-NIR-II luminescence in Pt^4+^/Er^3+^-codoped Cs_2_ZrCl_6_ double perovskite phosphors via energy transfer engineering for diversified applications. Laser Photonics Rev. 2025;19(13):2401940.

[46] Serna-Gallén P, Beltrán-Mir H, Cordoncillo E, West AR, Balda R, Fernández J. Site-selective symmetries of Eu^3+^-doped BaTiO_3_ ceramics: A structural elucidation by optical spectroscopy. J Mater Chem C. 2019;7(44):13976–13985.

[47] Zhang X, Wang Y, Wu X, Wang F, Ou Q, Zhang S. A comprehensive review on mechanisms and applications of rare-earth based perovskite nanocrystals. Chin J Chem. 2024;42(9):1032–1056.

[48] Liu Y, Rong X, Li M, Molokeev MS, Zhao J, Xia Z. Incorporating rare-earth terbium(III) ions into Cs_2_AgInCl_6_: Bi nanocrystals toward tunable photoluminescence. Angew Chem Int Ed. 2020;59(28):11634–11640. 10.1002/anie.202004562 32329132

[49] Li G, Tian Y, Zhao Y, Lin J. Recent progress in luminescence tuning of Ce^3+^ and Eu^2+^-activated phosphors for pc-WLEDs. Chem Soc Rev. 2015;44(23):8688–8713. 26421319 10.1039/c4cs00446a

[50] Li H, Zhang M, Li Y, Fu X, Feng J, Zhang H. Efficient multi-luminescence covering the visible to near-infrared range in antimony and lanthanide co-doped indium-based zero-dimensional perovskites nanocrystals. Adv Opt Mater. 2023;11(17):2300429.

[51] Stefanski M, Bondzior B, Basinski A, Ptak M, Lou B, Ma C-G. Synthesis and characterization of a CsPbCl_3_ perovskite doped with Nd^3+^: Structural, optical, and energy transfer properties. Inorg Chem Front. 2024;11(9):2626–2633.

[52] Zhou B, Muhammad Y, Ding D, Liu Z, Hu H, Wang Y, Zhong H, Shi Y, Li H. Dexter energy transfer in zero-dimensional inorganic metal halides for obtaining near-unity PLQY via Sb^3+^/Mn^2+^ codoping. J Phys Chem C. 2024;128(1):571–579.

[53] Olaya-Castro A, Scholes GD. Energy transfer from Förster–Dexter theory to quantum coherent light-harvesting. Int Rev Phys Chem. 2011;30(1):49–77.

[54] Vyazovkin S, Burnham AK, Favergeon L, Koga N, Moukhina E, Pérez-Maqueda LA, Sbirrazzuoli N. ICTAC kinetics committee recommendations for analysis of multi-step kinetics. Thermochim Acta. 2020;689: Article 178597.

[55] Bünzli J-CG, Eliseeva SV. Basics of lanthanide photophysics. In: Hänninen P, Härmä H, editors. Lanthanide luminescence: Photophysical, analytical and biological aspects. Berlin (Heidelberg): Springer; 2011. p. 1–45.

[56] Dorenbos P. Thermal quenching of lanthanide luminescence via charge transfer states in inorganic materials. J Mater Chem C. 2023;11(24):8129–8145.

[57] Saidaminov MI, Mohammed OF, Bakr OM. Low-dimensional-networked metal halide perovskites: The next big thing. ACS Energy Lett. 2017;2(4):889–896.

[58] Gundiah G, Brennan K, Yan Z, Samulon EC, Wu G, Bizarri GA, Derenzo SE, Bourret-Courchesne ED. Structure and scintillation properties of Ce^3+^-activated Cs_2_NaLaCl_6_, Cs_3_LaCl_6_, Cs_2_NaLaBr_6_, Cs_3_LaBr_6_, Cs_2_NaLaI_6_ and Cs_3_LaI_6_. J Lumin. 2014;149:374–384.

[59] Zhang C, Feng X, Song Q, Zhou C, Peng L, Chen J, Liu X, Chen H, Lin J, Chen X. Blue-violet emission with near-unity photoluminescence quantum yield from Cu(I)-doped Rb_3_InCl_6_ single crystals. J Phys Chem Lett. 2021;12(33):7928–7934. 34387495 10.1021/acs.jpclett.1c01751

[60] Kachhap S, Singh S, Singh AK, Singh SK. Lanthanide-doped inorganic halide perovskites (CsPbX_3_): Novel properties and emerging applications. J Mater Chem C. 2022;10(10):3647–3676.

[61] Locardi F, Cirignano M, Baranov D, Dang Z, Prato M, Drago F, Ferretti M, Pinchetti V, Fanciulli M, Brovelli S, et al. Colloidal synthesis of double perovskite Cs_2_AgInCl_6_ and Mn-doped Cs_2_AgInCl_6_ nanocrystals. J Am Chem Soc. 2018;140(40):12989–12995. 30198712 10.1021/jacs.8b07983 PMC6284204

[62] Senden T, van Dijk-Moes RJA, Meijerink A. Quenching of the red Mn^4+^ luminescence in Mn^4+^-doped fluoride LED phosphors. Light Sci Appl. 2018;7(1):8. 30839606 10.1038/s41377-018-0013-1 PMC6106983

[63] Sharma AA, Pradhan PP, Prasad KAKD, Rakshita M, Pembarthi R, Haranath D. Multimodal luminescence and energy transfer mechanism in a narrowband UVB emitting phosphor system towards futuristic phototherapeutic devices. Mater Adv. 2025;6(22):8400–8413.

[64] Venkatramu V, Babu P, Martín IR, Lavín V, Muñoz-Santiuste JE, Tröster T, Sievers W, Wortmann G, Jayasankar CK. Role of the local structure and the energy trap centers in the quenching of luminescence of the Tb^3+^ ions in fluoroborate glasses: A high pressure study. J Chem Phys. 2010;132(11): Article 114505. 20331303 10.1063/1.3352631

[65] Zhu D, Zaffalon ML, Zito J, Cova F, Meinardi F, De Trizio L, Infante I, Brovelli S, Manna L. Sb-doped metal halide nanocrystals: A 0D versus 3D comparison. ACS Energy Lett. 2021;6(6):2283–2292. 34307878 10.1021/acsenergylett.1c00789 PMC8294020

[66] Fu P, Hu S, Tang J, Xiao Z. Material exploration via designing spatial arrangement of octahedral units: A case study of lead halide perovskites. Front Optoelectron. 2021;14(2):252–259. 36637668 10.1007/s12200-021-1227-z PMC9743903

[67] Zhang B, Ru Y, Zhou J, Jia J, Song H, Liu Z, Zhang L, Liu X, Zhong G-M, Yong X, et al. A robust anti-thermal-quenching phosphor based on zero-dimensional metal halide Rb_3_InCl_6_:xSb^3+^. J Am Chem Soc. 2024;146(11):7658–7667. 38452365 10.1021/jacs.3c14137

[68] Karluk AA, Nematulloev S, Thomas S, Hasanov BE, Yorov K, Naphade R, Gutiérrez-Arzaluz L, Mohammad B, Abulikemu M, Mahmood J, et al. Scalable synthesis of Sb-doped Rb_3_InCl_6_ nanocrystals for high-resolution x-ray imaging screen. Cell Rep Phys Sci. 2025;6(2): Article 102427.

[69] Yang Z, Cui J, Sun Y, Yao J, Yang S, Song J. Na-alloy tailored 3D/0D metal halide heterostructures enabling efficient charge transfer for color-integrated white scintillators. Nat Commun. 2025;16(1):6909. 40715131 10.1038/s41467-025-62195-x PMC12297683

[70] Saidaminov MI, Almutlaq J, Sarmah S, Dursun I, Zhumekenov AA, Begum R, Pan J, Cho N, Mohammed OF, Bakr OM. Pure Cs_4_PbBr_6_: Highly luminescent zero-dimensional perovskite solids. ACS Energy Lett. 2016;1(4):840–845.

[71] Zhang F, Ji X, Liang W, Li Y, Ma Z, Wang M, Wang Y, Wu D, Chen X, Yang D. Room-temperature synthesis of blue-emissive zero-dimensional cesium indium halide quantum dots for temperature-stable down-conversion white light-emitting diodes with a half-lifetime of 186 h. Mater Horiz. 2021;8(12):3432–3442. 34700333 10.1039/d1mh01370j

[72] Li H, Tian L, Yang Q, Wang N, Fu X, Zhou L, Feng J, Zhang H. Sb^3+^ doped Cs_3_YCl_6_ metal halides with highly efficient broad-band green emission for light-emitting diode and x-ray imaging. SCIENCE CHINA Chem. 2025;68(6):2459–2466.

[73] Wang L, Guo Q, Duan J, Xie W, Ji G, Li S, Chen C, Li J, Yang L, Tan Z. Exploration of nontoxic Cs_3_CeBr_6_ for violet light-emitting diodes. ACS Energy Lett. 2021;6(12):4245–4254.

[74] Hou C, Liu X, Wan C, Li B, Lu T, Ge C, Song Y, Wang A, Kang Y, Dong Q. Designing guanidine-based antimony halides luminescence perovskite crystals toward near-unity quantum yield. Chem Mater. 2023;35(24):10635–10644.

[75] Yu L, Wang Y, Xing X, Xu Z, Shang M. Enhancing the thermal stability of the NIR emitting A_2_InCl_5_·H_2_O:Cr^3+^ phosphor based on A site regulation. J Mater Chem C. 2024;12(33):13021–13028.

[76] Cao Y, Wang N, Tian H, Guo J, Wei Y, Chen H, Miao Y, Zou W, Pan K, He Y. Perovskite light-emitting diodes based on spontaneously formed submicrometre-scale structures. Nature. 2018;562(7726):249–253. 30305742 10.1038/s41586-018-0576-2

[77] Singh K, Singh H, Sharma G, Gerward L, Khanna A, Kumar R, Nathuram R, Sahota HS. Gamma-ray shielding properties of CaO–SrO–B2O_3_ glasses. Radiat Phys Chem. 2005;72(2-3):225–228.

[78] Attix FH (Ed). Introduction to radiological physics and radiation dosimetry. Hoboken (NJ): John Wiley & Sons; 2008.

[79] Knoll GF (Ed). Radiation detection and measurement. Hoboken (NJ): John Wiley & Sons; 2010.

[80] Yuge Zhang GM, Wang P, Bao Z, Ouyang X, Liu L, Ouyang X. Light yield measurement technology and optimization methods of x-ray imaging scintillator. Chin J Lumin. 2025;46(4):630–641.

[81] Miller SR, Gaysinskiy V, Shestakova I, Nagarkar VV. Recent advances in columnar CsI(Tl) scintillator screens. In: *Penetrating radiation systems and applications VII*. Bellingham (WA): SPIE; 2005. vol. 5923. p. 99–108.

[82] Krupčík J, Májek P, Gorovenko R, Blaško J, Kubinec R, Sandra P. Considerations on the determination of the limit of detection and the limit of quantification in one-dimensional and comprehensive two-dimensional gas chromatography. J Chromatogr A. 2015;1396:117–130. 25907667 10.1016/j.chroma.2015.03.084

[83] Li H, Li K, Li Z, Fu X, Yang Q, Wang N, Wang X, Feng J, Song S, Zhang H. Lanthanide-based metal halides prepared at room temperature by recrystallization method for x-ray imaging. Light Sci Appl. 2025;14(1):195. 40368931 10.1038/s41377-025-01839-5 PMC12078671

[84] Kasap S, Frey JB, Belev G, Tousignant O, Mani H, Greenspan J, Laperriere L, Bubon O, Reznik A, DeCrescenzo G, et al. Amorphous and polycrystalline photoconductors for direct conversion flat panel x-ray image sensors. Sensors. 2011;11(5):5112–5157. 22163893 10.3390/s110505112 PMC3231396

[85] Hafızoğlu N. Efficiency and energy resolution of gamma spectrometry system with HPGe detector depending on variable source-to-detector distances. Eur Phys J Plus. 2024;139(2):134.

[86] Zhou L, Liao J-F, Huang Z-G, Wei J-H, Wang X-D, Li W-G, Chen H-Y, Kuang D-B, Su C-Y. A highly red-emissive lead-free indium-based perovskite single crystal for sensitive water detection. Angew Chem Int Ed Engl. 2019;58(16):5277–5281. 30788885 10.1002/anie.201814564

[87] Han P, Luo C, Yang S, Yang Y, Deng W, Han K. All-inorganic l0ead-free 0D perovskites by a doping strategy to achieve a PLQY boost from <2 % to 90 %. Angew Chem Int Ed Engl. 2020;59(31):12709–12713. 32337797 10.1002/anie.202003234

[88] Li X, Wang Z, Sun H, Bai F, Xu S, Wang C. Solvent stimuli-responsive off-on fluorescence induced by synergistic effect of doping and phase transformation for Te^4+^ doped indium halide perovskite: Giving printable and colorless ink for information encryption and decryption. J Colloid Interface Sci. 2023;633:808–816. 36493745 10.1016/j.jcis.2022.11.132

[89] Huang J, Chang T, Zeng R, Yan J, Wei Q, Zhou W, Cao S, Zou B. Controlled structural transformation in Sb-doped indium halides A_3_InCl_6_ and A_2_InCl_5_·H_2_O yields reversible green-to-yellow emission switch. Adv Opt Mater. 2021;9(13):2002267.

[90] Zhang F, Yang D, Shi Z, Qin C, Cui M, Ma Z, Wang L, Wang M, Ji X, Chen X, et al. Stable zero-dimensional cesium indium bromide hollow nanocrystals emitting blue light from self-trapped excitons. Nano Today. 2021;38: Article 101153.

[91] Meng W, Wang X, Xiao Z, Wang J, Mitzi DB, Yan Y. Parity-forbidden transitions and their impact on the optical absorption properties of lead-free metal halide perovskites and double perovskites. J Phys Chem Lett. 2017;8(13):2999–3007. 28604009 10.1021/acs.jpclett.7b01042

[92] Song H, Yuan J, Mu D, Han L, Xu X, Zhang P. Te^4+^ doped zero-dimensional perovskites for dual-mode thermometry in electronic devices. J Mater Chem C. 2025;13(7):3465–3474.

[93] Chen S, Li X, Zhang J, Pan D, Kong Y. Cation-substitution-induced high spectral tunability and thermosensitivity in double halide perovskite Cs_2_InCl_5_·H_2_O. Opt Mater. 2024;156: Article 115974.

[94] Zhou B, Fang F, Liu Z, Zhong H, Zhou K, Hu H, Min J, Zheng F, Fang S, Nie J, et al. Self-trapped Exciton emission in highly polar 0D hybrid ammonium/hydronium-based perovskites triggered by antimony doping. J Am Chem Soc. 2024;146(22):15198–15208. 38743271 10.1021/jacs.4c02108

[95] Liu Z, Zhou B, Fang S, Nie J, Zhong H, Hu H, Li H, Shi Y. Modulation of the excitation states in all-inorganic halide perovskites via Sb^3+^ and Bi^3+^ codoping. J Phys Chem Lett. 2023;14(4):1022–1028. 36693161 10.1021/acs.jpclett.2c03658

[96] Zhou B, Liu Z, Fang S, Nie J, Zhong H, Hu H, Li H, Shi Y. Emission mechanism of self-trapped excitons in Sb^3+^-doped all-inorganic metal-halide perovskites. J Phys Chem Lett. 2022;13(39):9140–9147. 36165781 10.1021/acs.jpclett.2c02759

[97] Zhang G, Dang P, Lian H, Li K, Tian L, Yang W, Cheng Z, Lin J. Assembling two self-trapped exciton emissions in 0D metal halides with near-unity quantum yield and zero thermal-quenching photoluminescence. Laser Photonics Rev. 2024;18(1):2300599.

[98] Hu B, Dong G, Li X, Zhang J, Chen C, Zhou J, Kong D, Wu W. Rb_2_InCl_5_·H_2_O doped with Te^4+^ for fluorescence thermometry and coordinated water-related structural phase transformation. Opt Lett. 2024;49(21):6205–6208. 39485448 10.1364/OL.541142

[99] Wei J-H, Luo J-B, Liao J-F, Ou W-T, Kuang D-B. Te^4+^-doped Cs_2_InCl_5_·H_2_O single crystals for remote optical thermometry. Sci China-Mater. 2022;65(3):764–772.

[100] Chen D, Wu C, Li H, Zhou L, Chen P, Pang Q, Zhang X, Zhang JZ. Near-infrared emission, energy transfer, and mechanisms of Mn^2+^ and Cr^3+^ Codoped lead-free Cs 2 AgInCl 6 double perovskites. J Mater Chem C. 2023;11(37):12649–12657.

[101] Liu Z, Qin X, Liu X. Luminescence enrichment in perovskite-lanthanide composites: Complexity and complementarity. In: *Handbook on the physics and chemistry of rare earths*. Amsterdam (Netherlands): Elsevier; 2022. vol. 61. p. 1–29.

[102] Sheng Y, Chen P, Gao Y, He Y, Li J, Muhammad, Xie X, Cheng C, Yang JT, Chang YJ, et al. Tuneable efficient white emission of Sb^3+^/Mn^2+^ co-doped lead-free perovskites for single-component white light-emitting diodes. ACS Appl Mater Interfaces. 2024;16(15):19175–19183. 38573052 10.1021/acsami.4c00745

[103] Jing L, Cen Q, Pang Q, Zhang JZ. Enhancing photoluminescence and stability of Mn-doped Cs_2_InCl_5_·H_2_O microcrystals with introduced Bi^3+^ ion. J Phys Chem C. 2023;127(5):2448–2455.

[104] Shao H, Wu X, Zhu J, Xu W, Xu L, Dong B, Hu J, Dong B, Bai X, Cui H, et al. Mn^2+^ ions doped lead-free zero-dimensional K_3_SbCl_6_ perovskite nanocrystals towards white light emitting diodes. Chem Eng J. 2021;413: Article 127415.

[105] Fan Q, Xu H, Zhu Z-K, Zhao Z, Rong H, Zhu P, Guo W, Tang L, Zhang J, Luo J, et al. 3D lead-free double perovskite via anchoring A-site cation for ultralow dose and stable x-ray detection. Adv Funct Mater. 2025;35(37):2505546.

[106] Mabate TP, Maqunga NP, Ntshibongo S, Maumela M, Bingwa N. Metal oxides and their roles in heterogeneous catalysis: Special emphasis on synthesis protocols, intrinsic properties, and their influence in transfer hydrogenation reactions. SN Appl Sci. 2023;5(7):196.

[107] Sahoo K, Kumar S, Mohapatra A, Dubey NK, Naik R, Goswami C, Bhaumik S. Highly stable and luminescent formamidinium-based perovskite nanocrystal probe for temperature and mercury sensors and in vitro imaging in live cells. J Mater Chem C. 2024;12(42):17315–17327.

[108] Li M, Wang S, Wood A, Yeager JD, Stepanoff SP, Adler JC, Shi Z, Wang J, Li Z, Wolfe DE. Defect repairing in lead bromide perovskite single crystals with biasing and bromine for x-ray photon-counting detectors. Nat Mater. 2025;24(12):1993–2000. 40750832 10.1038/s41563-025-02310-x PMC12450060

[109] Mo Q, Yu J, Chen C, Cai W, Zhao S, Li H, Zang Z. Highly efficient and ultra-broadband yellow emission of lead-free antimony halide toward white light-emitting diodes and visible light communication. Laser Photonics Rev. 2022;16(10):2100600.

[110] Morad V, Yakunin S, Kovalenko MV. Supramolecular approach for fine-tuning of the bright luminescence from zero-dimensional antimony(III) halides. ACS Materi Lett. 2020;2(7):845–852. 10.1021/acsmaterialslett.0c00174 PMC7493224 32954358

[111] Song Y, Li L, Bi W, Hao M, Kang Y, Wang A, Wang Z, Li H, Li X, Fang Y, et al. Atomistic surface passivation of CH_3_NH_3_PbI_3_ perovskite single crystals for highly sensitive coplanar-structure x-ray detectors. Research. 2020;2020: Article 5958243. 33043296 10.34133/2020/5958243 PMC7528034

[112] Chen D, Dai F, Hao S, Zhou G, Liu Q, Wolverton C, Zhao J, Xia Z. Crystal structure and luminescence properties of lead-free metal halides (C_6_H_5_CH_2_NH_3_)_3_MBr_6_ (M = Bi and Sb). J Mater Chem C. 2020;8(22):7322–7329.

[113] Zhizhuan Zhang JJ, Gong L, Kezhao D, Huang X. Two new antimony (III) chloride hybrids composed of mononuclear SbCl_6_. ^3−^ unit and ionic liquid cations with different length of alkyl chain. Chinese Journal of Structural Chemistry. 2021.

[114] Mirochnik AG, Petrochenkova N, Storozhuk TV, Lifar LI. Synthesis, structure, and luminescent properties of antimony(III) complexes with *N*,*N*-diphenylguanidine. Russ J Coord Chem. 2001;27:783–785.

[115] Peng Y-C, Zhang Z-Z, Lin Y-P, Jin J-C, Zhuang T-H, Gong L-K, Wang Z-P, Du K-Z, Huang X-Y. A deep-red-emission antimony(III) chloride with dual-cations: Extremely large stokes shift due to high SbCl_6_ distortion. Chem Commun. 2021;57(100):13784–13787. 10.1039/d1cc05648d 34860224

[116] Zhao J-Q, Shi H-S, Zeng L-R, Ge H, Hou Y-H, Wu X-M, Yue C-Y, Lei X-W. Highly emissive zero-dimensional antimony halide for anti-counterfeiting and confidential information encryption-decryption. Chem Eng J. 2022;431: Article 134336.

[117] Wang Z, Huang X. Stepwise structural transformation in hybrid antimony chloride for time-resolved and multi-stage informational encryption and anti-counterfeiting. Inorg Chem Front. 2025;12(12):3997–4006.

[118] Wang Z, Zhang Z, Tao L, Shen N, Hu B, Gong L, Li J, Chen X, Huang X. Hybrid chloroantimonates(III): Thermally induced triple-mode reversible luminescent switching and laser-printable rewritable luminescent paper. Angew Chem Int Ed Engl. 2019;58(29):9974–9978. 31070295 10.1002/anie.201903945

[119] Wang Y, Wang Y, Zhan X, Shi H, Zhu W, Chen B, Liu S, Xu X, Zhao Q. Off-stoichiometry synthesis of 0D metal halide polycrystals for high-performance x-ray imaging. Adv Opt Mater. 2024;12(27):2401092.

[120] Li H, He Y, Li W, Lu T, Tan M, Wei W, Yang B, Wei H. Perovskite dimensional evolution through cations engineering to tailor the detection limit in hard x-ray response. Small. 2022;18(42):2203884. 10.1002/smll.202203884 36117116

[121] Zaffalon ML, Wu Y, Cova F, Gironi L, Li X, Pinchetti V, Liu Y, Imran M, Cemmi A, Di Sarcina I, et al. Zero-dimensional Gua_3_SbCl_6_ crystals as intrinsically reabsorption-free scintillators for radiation detection. Adv Funct Mater. 2023;33(48):2305564.

[122] Yang H, Cai T, Liu E, Hills-Kimball K, Gao J, Chen O. Synthesis and transformation of zero-dimensional Cs_3_BiX_6_ (X = cl, Br) perovskite-analogue nanocrystals. Nano Res. 2020;13(1):282–291.

[123] Tang Y, Liang M, Chang B, Sun H, Zheng K, Pullerits T, Chi Q. Lead-free double halide perovskite Cs_3_BiBr_6_ with well-defined crystal structure and high thermal stability for optoelectronics. J Mater Chem C. 2019;7(11):3369–3374.

[124] Cao F, Yan T, Li Z, Wu L, Fang X. Dual-band perovskite bulk heterojunction self-powered photodetector for encrypted communication and imaging. Adv Opt Mater. 2022;10(18):2200786.

[125] Shimizu M, Koshimizu M, Fujimoto Y, Yanagida T, Ono S, Asai K. Luminescence and scintillation properties of Cs_3_BiCl_6_ crystals. Opt Mater. 2016;61:115–118.

[126] Tang Y, Gomez L, van der Laan M, Timmerman D, Sebastian V, Huang C-C, Gregorkiewicz T, Schall P. Room temperature synthesis and characterization of novel lead-free double perovskite nanocrystals with a stable and broadband emission. J Mater Chem C. 2021;9(1):158–163.

[127] Cao F, Li Z, Liu X, Shi Z, Fang X. Air induced formation of Cs_3_Bi_2_Br_9_/Cs_3_BiBr_6_ bulk heterojunction and its dual-band photodetection abilities for light communication. Adv Funct Mater. 2022;32(46):2206151.

[128] Ma J, Zhang F, Xing Y, Jiang H, Tian Y, Ji H, Chen X, Wu D, Zeng L, Li X, et al. Self-powered UV dual-band photodetector based on Cs_3_BiCl_6_/GaN heterojunction for logical operation and encrypted photo-communication. Adv Sci. 2025;12(28):2503498. 10.1002/advs.202503498 PMC12302572 40349180

[129] Wang G, Jin Z, Chen Y, Chen F, Chen Q. Solvent modulated different stacking of cobalt chelates and their molecular magnetic behaviors. J Mol Struct. 2025;1336: Article 142047.

[130] Jia Z, Gong P, Zhao J, Chen M, Wang Y, Wang Z, Dong Y, Xia M. Antimony-doped enhanced photoluminescence quantum yield in zero-dimensional lead-free metal halide Rb_2_CsBiCl_6_ crystals. Inorg Chem Front. 2022;9(23):6299–6304.

[131] Chen R, Wang S, Lin F, Zheng Y, Zhang W, Wang J, Guo F. Stable broadband bright green emission of self-trapped excitons triggered by Sb^3+^ doping in zero-dimensional Cs_3_BiCl_6_. Eur J Inorg Chem. 2024;27(1): Article e202300366.

[132] Jia Z, Gong P, Chen M, Wang Z, Li X, Song Y, Zhang S, Zhang N, Xia M. Antimony doping enabled photoluminescence quantum yield enhancement in 0D inorganic bismuth halide crystals. Inorg Chem. 2023;62(48):19690–19697. 38044827 10.1021/acs.inorgchem.3c03039

[133] Wu J, Zhang S, Yan J, Zou B, Zeng R. A new zero-dimensional (CsK_2_)BiCl_6_ metal halide: Boosting emission via B-site Mn-doping. Crystals. 2022;12(11):1681.

[134] Guo T, Ning X, Deng Z, Lin J, Zhou D, Wen M, Wang Q, Wen Y, Qiu J. Sb^3+^-doping induced quasi-2D single crystal growth from 0D Cs_3_BiCl_6_: Modulation of site-specific Cr^3+^ near-infrared emission. Adv Opt Mater. 2026;14(6): Article e02778.

[135] Takahashi K, Arai M, Koshimizu M, Fujimoto Y, Yanagida T, Asai K. Luminescence and scintillation characteristics of Cs_3_ScCl_6_:Ce crystals. Ceram Int. 2020;46(16):26346–26349.

[136] Wei H, Zhuravleva M, Meng F, Melcher CL. Temperature dependence spectroscopic study of Ce-doped Cs_3_LaCl_6_ and Cs_3_LaBr_6_ scintillators. J Lumin. 2015;160:64–70.

[137] Kim D, Yun T, An S, Lee C-L. How to improve the structural stabilities of halide perovskite quantum dots: Review of various strategies to enhance the structural stabilities of halide perovskite quantum dots. Nano Convergence. 2024;11(1):4. 38279984 10.1186/s40580-024-00412-x PMC10821855

[138] Zhang L, Sun C, He T, Jiang Y, Wei J, Huang Y, Yuan M. High-performance quasi-2D perovskite light-emitting diodes: From materials to devices. Light Sci Appl. 2021;10(1):61. 33741895 10.1038/s41377-021-00501-0 PMC7979804

[139] Lee M, Lee DHD, Hong SV, Woo HY, Chae J-Y, Lee DW, Han MJ, Paik T. Highly luminescent and multifunctional zero-dimensional cesium lanthanide chloride (Cs_3_LnCl_6_) colloidal nanocrystals. Adv Opt Mater. 2022;10(9):2102727.

[140] Zhang R, Xu X, Mao X, Wang Z, Wang P, Yang Y, Chen J, Lu R, Deng W, Han K. Excitation-dependent emission in all-inorganic lead-free Cs_2_ScCl_5_·H_2_O perovskite crystals. Laser Photonics Rev. 2022;16(5):2100689.

[141] Huang Y, Pan Y, Peng C, Ding Y, Lian H, Li L, Lin J. Orange/cyan emissive sensors of Sb^3+^ for probing water via reversible phase transformation in rare-earth-based perovskite crystals. Inorg Chem Front. 2023;10(3):991–1000.

[142] Samanta T, Viswanath NSM, Kim HW, Jang SW, Han JH, Cho SB, Im WB. Demystifying role of local distortion in emission colors tuning of lead-free zero-dimensional metal halide nanocrystals. Chem Eng J. 2024;484: Article 149697.

[143] Samanta T, Viswanath NSM, Jang SW, Min JW, Cho HB, Han JH, Im WB. Thermally stable self-trapped assisted single-component white light from lead-free zero-dimensional metal halide nanocrystals. Adv Opt Mater. 2023;11(9):2202744.

[144] Xie W, Wang T, Xu H, Liang H, Shen H, Xue H, Wang X, Xu F, Zhang J, Sun L, et al. A record efficiency of 1070 nm near-infrared light-emitting diodes based on Cs_3_La_1−x_Nd_x_X_6_ perovskite nanocrystals. Adv Funct Mater. 2026; Article e31915.

[145] Mao X, Wang Z, Zhang F, Yin H, Xu X, Chen J, Chen Z, Luo J, Han K, Zhang R. All-inorganic zero-dimensional Sb^3+^-doped Rb_2_ScCl_5_(H_2_O) perovskite single crystals: Efficient self-trapped exciton emission and x-ray detection. J Phys Chem Lett. 2023;14(6):1521–1527. 36745062 10.1021/acs.jpclett.2c03912

[146] Combes CM, Dorenbos P, van Eijk CWE, Krämer KW, Güdel HU. Optical and scintillation properties of pure and Ce^3+^-doped Cs_2_LiYCl_6_ and Li_3_YCl_6_:Ce^3+^ crystals. J Lumin. 1999;82(4):299–305.

[147] Lee M, Chung H, Hong SV, Woo HY, Chae J-Y, Yoon TY, Diroll BT, Paik T. Dynamically tunable multicolor emissions from zero-dimensional Cs_3_LnCl_6_ (ln: Europium and terbium) nanocrystals with wide color gamut. Nanoscale. 2023;15(4):1513–1521. 36472217 10.1039/d2nr04771c

[148] Yin H, Zhou X, Song B, Wang R, Li B, Sun J, Wei Q, Cai P, Chen Z, Yang F, et al. The scintillating dynamics of self-trapped exciton endowed/unendowed by thermally activated delayed fluorescence. Laser Photonics Rev. 2025;19(6):2401508.

[149] Liang X, Xia H-L, Xiang J, Wang F, Ma J, Zhou X, Wang H, Liu X-Y, Zhu Q, Lin H, et al. Facile tailoring of metal-organic frameworks for Förster resonance energy transfer-driven enhancement in perovskite photovoltaics. Adv Sci. 2024;11(18):2307476. 10.1002/advs.202307476 PMC11095144 38445968

[150] Sun L, Dong B, Sun J, Wang Y, Wang Y, Hu S, Zhou B, Bai X, Xu L, Zhou D. Efficient and stable multicolor emissions of the coumarin-modified Cs_3_LnCl_6_ Lead-free perovskite nanocrystals and LED application. Adv Mater. 2024;36(18):2310065. 10.1002/adma.202310065 38290534

[151] Wu Y, Shi H, Chakoumakos BC, Zhuravleva M, Du M-H, Melcher CL. Crystal structure, electronic structure, temperature-dependent optical and scintillation properties of CsCe_2_Br_7_. J Mater Chem C. 2015;3(43):11366–11376.

[152] Xie W, Hu F, Gong S, Peng L. Study the optical properties of Cs_3_CeI_6_: First-principles calculations. AIP Adv. 2024;14(1): Article 015062.

[153] Butman MF, Motalov VB, Kudin LS, Rycerz L, Gaune-Escard M. Thermodynamic characterization of the congruently melting Cs_3_CeCl_6_ compound. J Chem Eng Data. 2008;53(10):2346–2350.

[154] Madarász J, Leskelä T, Pokol G, Niinistö L. Thermally induced changes in the oxidation state of cerium(IV). J Therm Anal. 1997;49(3):1347–1355.

[155] Min JW, Samanta T, Lee AY, Jung YK, Viswanath NSM, Kim YR, Cho HB, Moon JY, Jang SH, Kim JH. Highly emissive lanthanide-based 0D metal halide nanocrystals for efficient ultraviolet photodetector. Small. 2024;20(43):2402951. 10.1002/smll.202402951 38923817

[156] Bai X, Liao J, Lou B, Zhang Y, Zhao H, Zi Y, Li Y, Tang W, Mandl GA, Huang A, et al. Reversible photochromism and multicolor luminescence modulation for x-ray detection and secure information encryption. Adv Funct Mater. 2026;36(4): Article e06583.

[157] Xie H, Zheng C, Lei Y, Peng G, Wu Y, Li Z, Wang Q, Jin Z. Single-source evaporated pixelated Cs_3_CeBr_6_ crystals based scintillator film for high spatial resolution x-ray imaging. Adv Funct Mater. 2026; Article e29234.

[158] Chai Z, Wang Z, Ke D, Zeng M, Li G, Yang L, Peng X, Li X, Li Y. Thermally robust 0D Cs_3_EuCl_6_ microcrystals for x-ray imaging. Small. 2025;21(38): Article e05594. 40761157 10.1002/smll.202505594

[159] Zhao C, Wang Y, Bao S, Zang Y, Liu X, Huang W. High-performance hybrid organic-inorganic lanthanide halide glass scintillators enabled by dehydration for efficient x-ray imaging. Adv Mater. 2025;37(21):2500925. 10.1002/adma.202500925 40166804

[160] Lindsey AC, Zhuravleva M, Melcher CL. Growth of CsCe_2_Cl_7_ and Cs_3_CeCl_6_ utilizing the Bridgman method. In: *2013 IEEE nuclear science symposium and medical imaging conference (2013 NSS/MIC)*. Piscataway (NJ): IEEE; 2013. p. 1–5.

[161] Samanta T, Han JH, Lee HU, Cha BK, Park YM, Viswanath NSM, Cho HB, Kim HW, Cho SB, Im WB. Large-scale mechanochemical synthesis of cesium lanthanide chloride for radioluminescence. Inorg Chem. 2024;63(35):16483–16490. 39171850 10.1021/acs.inorgchem.4c02766

[162] Sun H, Yang X, Li P, Bai Y, Meng Q, Zhao H, Wang Q, Wen Z, Huang L, Huang D, et al. Solution synthesis and light-emitting applications of one-dimensional lead-free cerium(III) metal halides. Nano Lett. 2024;24(33):10355–10361. 39119944 10.1021/acs.nanolett.4c03019

[163] Fujimoto Y, Nakauchi D, Yanagida T, Koshimizu M, Asai K. New intrinsic fast scintillator: Cesium praseodymium chloride. Sens Mater. 2022;34(2):629–636.

[164] Oomen EWJL, Smit WMA, Blasse G. Jahn-Teller effect in the Sb^3+^ emission in zircon-structured phosphates. Chem Phys Lett. 1984;112(6):547–550.

[165] Guo X-X, Chen J-H, Luo J-B, Wei J-H, Zhang Z-Z, He Z-L, Peng Q-P, Kuang D-B. All-inorganic Cs_2_YbCl_5_·H_2_O perovskite with luminescence response to methanol for anti-counterfeiting. Adv Opt Mater. 2024;12(22):2400681.

[166] Ma W, Xu D, Zhao L, Xuan T, Wang X, Xie R-J, Wang X. Sb^3+^-activated 0D Cs_3_LuCl_6_: A cyan–green phosphor with near-unity quantum yield for ultrahigh-CRI full-spectrum white LEDs. Adv Opt Mater. 2025;13(36): Article e03160.

[167] Gao R, Xiang J, Ding X, Chen C, Chang J, Chen R, Yang Z, Guo C. Sb-doped Cs_3_LuCl_6_ nanocrystals for efficient x-ray imaging. Chin Opt Lett. 2026;24(2): Article 021601.

[168] Chen J, Guo Y, Chen B, Zheng W, Zhang X, Wei X, Cao Y, Suo H, Wang F. Kinetics-tunable hydrochromic luminescence switching in Rb_3_TbF_6_:Eu^3+^ perovskite. Adv Opt Mater. 2024;12(18):2400147.

[169] Chen J, Guo Y, Chen B, Zheng W, Wang F. Ultrafast and multicolor luminescence switching in a lanthanide-based hydrochromic perovskite. J Am Chem Soc. 2022;144(48):22295–22301. 36417793 10.1021/jacs.2c10809

[170] Xiong H, Dong J, Yang J, Liu Y, Song H, Gan S. Facile hydrothermal synthesis and multicolor-tunable luminescence of YPO 4: Ln^3+^(Ln= Eu, Tb). RSC Adv. 2016;6(100):98208–98215.

[171] Samanta T, Yadav AN, Han JH, Kim M, Jang SW, Viswanath NSM, Im WB. Cerium-sensitized highly emissive 0D cesium cerium terbium chloride alloy nanocrystals for white light emission. Adv Opt Mater. 2024;12(23):2400909.

[172] Wang T-C, He Z-L, Luo J-B, Peng Q-P, Wei J-H, Chen K-L, Chen J-H, Guo X-X, Kuang D-B. Organic–inorganic hybrid rare earth halide glasses for tunable multicolor x-ray scintillation. Angew Chem Int Ed Engl. 2025;64(23): Article e202504658. 40192634 10.1002/anie.202504658

[173] Lian L, Xiong D, Fan Y, Liang T, Ai M, Zhang J, Jia M, Liu Y, Ma Z, Chen X, et al. Water-assisted rapid evaporation synthesis of transparent and multicolor luminescent lanthanide-based metal halide glasses for high-resolution x-ray imaging. Adv Funct Mater. 2025; Article e23727.

[174] Kim HW, Han JH, Ko H, Samanta T, Lee DG, Jeon DW, Kim W, Chung Y-C, Im WB, Cho SB. High-throughput screening on halide perovskite derivatives and rational design of Cs_3_LuCl_6_. ACS Energy Lett. 2023;8(8):3621–3630.

[175] Wang Z, Dujardin C, Freeman MS, Gehring AE, Hunter JF, Lecoq P, Liu W, Melcher CL, Morris CL, Nikl M, et al. Needs, trends, and advances in scintillators for radiographic imaging and tomography. IEEE Trans Nucl Sci. 2023;70(7):1244–1280.

[176] Pilania G, Liu X-Y, Wang Z. Data-enabled structure–property mappings for lanthanide-activated inorganic scintillators. J Mater Sci. 2019;54(11):8361–8380.

[177] Belsky A, Ivanovskikh K, A. Vasil’ev, M.-F. Joubert, C. Dujardin. Estimation of the electron thermalization length in ionic materials. J Phys Chem Lett. 2013;4(20):3534–3538.

[178] Luo J, Wang X, Li S, Liu J, Guo Y, Niu G, Yao L, Fu Y, Gao L, Dong Q, et al. Efficient and stable emission of warm-white light from lead-free halide double perovskites. Nature. 2018;563(7732):541–545. 30405238 10.1038/s41586-018-0691-0

[179] Marques MA, Gross EK. Time-dependent density functional theory. Annu Rev Phys Chem. 2004;55:427–455. 15117259 10.1146/annurev.physchem.55.091602.094449

[180] Tully JC. Molecular dynamics with electronic transitions. J Chem Phys. 1990;93(2):1061–1071.

[181] Feng P-Y, Sun X-L, An Z-H, Deng Y, Wang C-E, Jiang H, Li J-J, Zhang D-L, Li X-Q, Xiong S-L, et al. The energy response of LaBr_3_(Ce), LaBr_3_(Ce,Sr), and NaI(Tl) crystals for GECAM. Nucl Sci Tech. 2024;35(2):23.

[182] Zhao J-Q, Han M-F, Zhao X-J, Ma Y-Y, Jing C-Q, Pan H-M, Li D-Y, Yue C-Y, Lei X-W. Structural dimensionality modulation toward enhanced photoluminescence efficiencies of hybrid lead-free antimony halides. Adv Opt Mater. 2021;9(19):2100556.

[183] Lin F, Wang H, Liu W, Li J. Zero-dimensional ionic antimony halide inorganic–organic hybrid with strong greenish yellow emission. J Mater Chem C. 2020;8(22):7300–7303.

[184] Dorenbos P. Light output and energy resolution of Ce^3+^-doped scintillators. Nucl Instrum Methods Phys Res A. 2002;486(1):208–213.

[185] Dutta S, Yoo JH, Kwon SB, Dastgeer G, Yoon DH. Harnessing dual violet emission in cerium-based perovskite derivatives for solution-processed next-generation lighting. ACS Appl Opt Mater. 2025;3(5):1070–1077.

[186] Du H, Yang L, Pang J, Shen Z, Li J, Dong X, Luo Y, Luo J, Tang J. Vacuum-deposited Rb_3_CeI_6_ for deep-blue-light-emitting diodes. Opt Lett. 2023;48(11):2777–2780. 37262208 10.1364/OL.486168

[187] Wang T, Zhou D, Wang R, Wang Y, Li W, Liang J, Song H. Breaking efficiency barrier: Dual-channel energy transfer enables record 1540 nm NIR LEDs from Er^3+^-doped Cs_3_DyI_6_ nanocrystals. Adv Mater. 2026;38(1): Article e12712. 40952075 10.1002/adma.202512712

[188] Li X, Cui H, Zhong Y, Zhou X, Xu S, Liu S, Wang C. Lead-free Ce-doped perovskite scintillators with high figure of merit. J Energy Chem. 2024;99:74–82.

[189] Yang Z, Cui J, Gu L, Gao R, Wang T, Song J. Leveraging structural rigidity for thermal robust scintillation in rare-earth halide perovskites. Adv Powder Mater. 2026;5(2): Article 100371.

[190] Hawrami R, Ariesanti E, Farsoni A, Szydel D, Sabet H. Growth and evaluation of improved CsI:Tl and NaI:Tl scintillators. Crystals. 2022;12(11):1517.

[191] Taranyuk V, Gektin A, Kisil I, Kolesnikov A. NaI(Tl) and CsI(Tl) scintillation crystal growth by skull method. J Cryst Growth. 2011;318(1):820–822.

[192] Drozdowski W, Dorenbos P, Bos AJJ, Owens A, Quarati FGA. Gamma ray induced radiation damage in ∅1in×1in LaBr_3_:5%Ce. Radiat Meas. 2008;43(2):497–501.

[193] Shah KS, Glodo J, Klugerman M, Moses WW, Derenzo SE, Weber MJ. LaBr_3_:Ce scintillators for gamma-ray spectroscopy. IEEE Trans Nucl Sci. 2003;50(6):2410–2413.

[194] Yoshikawa A, Shoji Y, Yokota Y, Kurosawa S, Hayasaka S, Chani VI, Ito T, Kamada K, Ohashi Y, Kochurikhin V. Growth of 2 inch Eu-doped SrI_2_ single crystals for scintillator applications. J Cryst Growth. 2016;452:73–80.

[195] Alekhin MS, Khodyuk IV, de Haas JTM, Dorenbos P. Nonproportional response and energy resolution of pure SrI_2_ and SrI_2_:5%Eu scintillators. IEEE Trans Nucl Sci 2012;59(3):665–670

[196] Lecoq P. Development of new scintillators for medical applications. Nucl Instrum Methods Phys Res, Sect A. 2016;809:130–139.

[197] Xu K, Hu P, Zhang Q, He Q, Liu Q, Wang Y, Huang F, Leng J, Lin X, Shi Y, et al. Effect of annealing on lattice engineering and luminescence in LYSO:Ce scintillators. CrystEngComm. 2026.

[198] Lee D, Kim M, Woo H-Y, Chae J, Lee D, Jeon S, Oh SJ, Paik T. Heating-up synthesis of cesium bismuth bromide perovskite nanocrystals with tailored composition, morphology, and optical properties. RSC Adv. 2020;10(12):7126–7133. 35493861 10.1039/c9ra10106c PMC9049756

[199] Shi L, Li J, Liu Z, Chen P, Jiang Y. Achieving near-unity photoluminescence quantum yield in lead-free cerium(III) halides Cs_3_CeX_6_ (X = Cl, Br, I) for multifunctional applications. Adv Opt Mater. 2026;14(2): Article e01624.

