# Supplementary figures and images for: Recent Advances in Cation-Engineered A3BX6 Metal Halide Perovskite for Enhanced Radiative Transition
Source: Research (Wash D C). 2026 May 18;9:1281. doi: 10.34133/research.1281 (PMC13181171; doi:10.34133/research.1281)

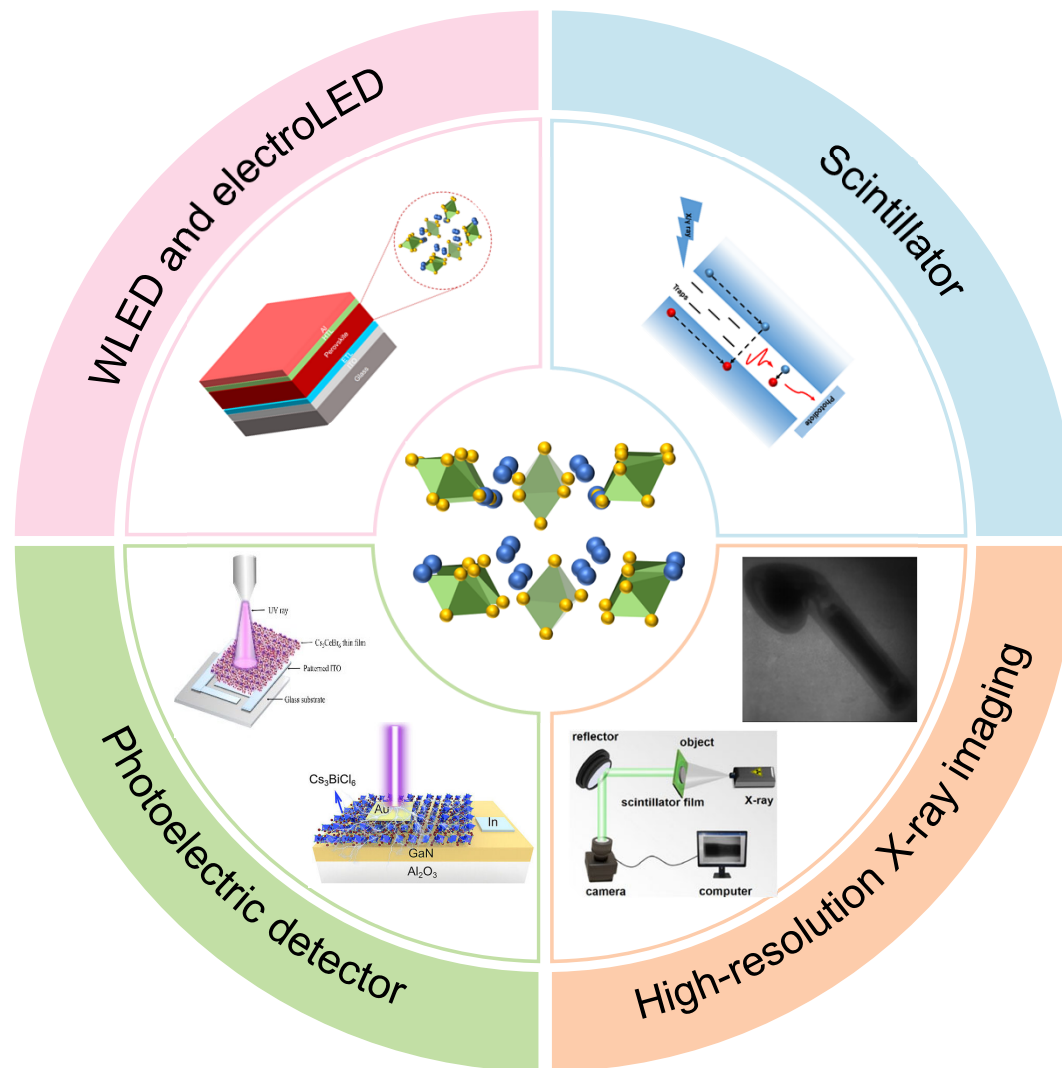

Supplement: Supplementary 1 — Fig. S1 [file research.1281.f1.pdf]
